# Magnetoelectric nanodiscs enable wireless transgene-free neuromodulation

**DOI:** 10.1038/s41565-024-01798-9

**Published:** 2024-10-11

**Authors:** Ye Ji Kim, Noah Kent, Emmanuel Vargas Paniagua, Nicolette Driscoll, Anthony Tabet, Florian Koehler, Elian Malkin, Ethan Frey, Marie Manthey, Atharva Sahasrabudhe, Taylor M. Cannon, Keisuke Nagao, David Mankus, Margaret Bisher, Giovanni de Nola, Abigail Lytton-Jean, Lorenzo Signorelli, Danijela Gregurec, Polina Anikeeva

**Affiliations:** 1 Department of Materials Science and Engineering, Massachusetts Institute of Technology, Cambridge, MA USA; 2 Research Laboratory of Electronics, Massachusetts Institute of Technology, Cambridge, MA USA; 3 McGovern Institute for Brain Research, Massachusetts Institute of Technology, Cambridge, MA USA; 4 Department of Electrical Engineering and Computer Science, Massachusetts Institute of Technology, Cambridge, MA USA; 5 Department Engineering in Computation and Cognition, Massachusetts Institute of Technology, Cambridge, MA USA; 6 Department of Chemistry, Massachusetts Institute of Technology, Cambridge, MA USA; 7 Koch Institute for Integrative Cancer Research, Massachusetts Institute of Technology, Cambridge, MA USA; 8 Department of Chemistry and Pharmacy, Friedrich-Alexander University of Erlangen–Nuremberg, Erlangen, Germany; 9 Department of Brain and Cognitive Sciences, Massachusetts Institute of Technology, Cambridge, MA USA

**Keywords:** Nanobiotechnology, Nanobiotechnology, Magnetic properties and materials

## Abstract

Deep brain stimulation with implanted electrodes has transformed neuroscience studies and treatment of neurological and psychiatric conditions. Discovering less invasive alternatives to deep brain stimulation could expand its clinical and research applications. Nanomaterial-mediated transduction of magnetic fields into electric potentials has been explored as a means for remote neuromodulation. Here we synthesize magnetoelectric nanodiscs (MENDs) with a core–double-shell Fe_3_O_4_–CoFe_2_O_4_–BaTiO_3_ architecture (250 nm diameter and 50 nm thickness) with efficient magnetoelectric coupling. We find robust responses to magnetic field stimulation in neurons decorated with MENDs at a density of 1 µg mm^−2^ despite individual-particle potentials below the neuronal excitation threshold. We propose a model for repetitive subthreshold depolarization that, combined with cable theory, supports our observations in vitro and informs magnetoelectric stimulation in vivo. Injected into the ventral tegmental area or the subthalamic nucleus of genetically intact mice at concentrations of 1 mg ml^−1^, MENDs enable remote control of reward or motor behaviours, respectively. These findings set the stage for mechanistic optimization of magnetoelectric neuromodulation towards applications in neuroscience research.

## Abstract (web summary)

In this study, the authors present magnetoelectric nanodiscs that enable minimally invasive, remote magnetic neuromodulation with subsecond precision to drive reward and motor behaviours in genetically intact mice.

## Main

Deep brain stimulation (DBS) via surgically implanted electrodes is a powerful therapeutic tool for neurological and psychiatric conditions^1^. Less invasive neuromodulation alternatives have been developed on the basis of transcranial magnetic stimulation^2^, temporal interference electrical stimulation^3,4^, focused ultrasound^5–7^ and optogenetics with external light sources^8,9^. In addition, weak magnetic fields (MFs) have been leveraged to deliver signals to deep brain structures owing to the low conductivity and magnetic permeability of biological matter^10^. MFs have been transduced into mechanical torque^11,12^, heat^13–16^ and chemical release^17,18^ enabling modulation of cells expressing mechano-, thermo- and chemo-receptors, respectively. Although genetic sensitization of identifiable cells to specified stimuli empowers fundamental neuroscience research, the need for transgenes impeded the implementation of these methods in translational and clinical studies.

To eliminate the need for transgenes and recapitulate the effects of clinical DBS, MFs can be converted directly into electrical signals via magnetoelectric (ME) transducers^19–21^. ME transduction at the millimetre scale has been applied to power miniature electrical stimulators^21–23^. To enable ME neuromodulation at the single-cell level, MF must be converted into electrical polarization at the nanoscale. ME nanoparticles (MENPs) of CoFe_2_O_4_–BaTiO_3_ were shown to generate single-particle voltages of ~8 µV, with the theoretical limit of ~1 mV in physiologically safe MF conditions^20,24^. These values are two to three orders of magnitude lower than the changes in membrane voltage necessary to trigger action potentials in neurons. Consequently, MENPs have been employed as concentrated microscale aggregates^20^.

**Fig. 1:**
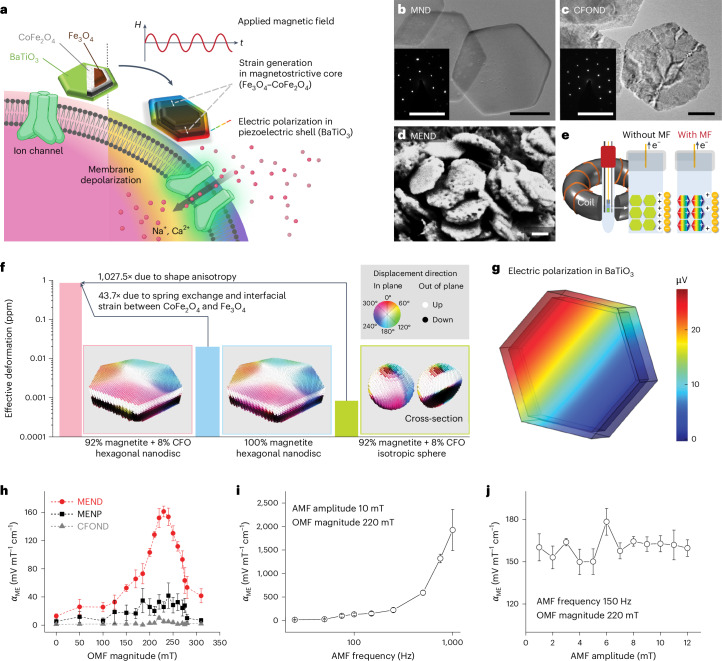
MENDs for neuromodulation. **a**, An illustration of neuromodulation mediated by MENDs. **b**,**c**, TEM images of Fe_3_O_4_ MNDs, which form the core of MENDs (**b**), and CFONDs (**c**). Scale bars, 100 nm. The insets show selected area electron diffraction patterns. Scale bars, 10 nm^−1^. **d**, SEM images of core–double-shell Fe_3_O_4_–CoFe_2_O_4_–BaTiO_3_ MENDs. Scale bar, 100 nm. **e**, An illustration of the electrochemical measurement apparatus that employs surface charge variation of MENDs in response to applied MF to determine the ME coefficient (${\alpha }_{{{\mathrm{ME}}}}$). **f**, Simulated magnetostriction constant (bars) and magnetostrictive displacement maps (framed insets) for hexagonal CFONDs (pink), hexagonal MNDs (blue) and Fe_3_O_4_–CoFe_2_O_4_ core–shell spherical nanoparticles (green). Inset: colour index for direction in magnetostrictive displacement maps. **g**, Simulated electric polarization generated in BaTiO_3_ shells deposited onto CFONDs upon exposure to an OMF of *H*_OMF_ = 220 mT and an AMF with an amplitude *H*_AMF_ = 10 mT. **h**, ${\alpha }_{{{\mathrm{ME}}}}$ at an AMF with a frequency ƒ_AMF_ = 150 Hz and *H*_AMF_ = 10 mT measured at varying *H*_OMF_ for MENDs (red), isotropic MENPs (black) and CFONDs (grey). **i**, ${\alpha }_{{{\mathrm{ME}}}}$ for MENDs as a function of AMF frequency at *H*_AMF_ = 10 mT and *H*_OMF_ = 220 mT. **j**, ${\alpha }_{{{\mathrm{ME}}}}$ for MENDs as a function of AMF amplitude for ƒ_AMF_ = 150 Hz and *H*_OMF_ = 220 mT. In **h**–**j**, the points and error bars indicate the mean and s.d. for *n* = 3 samples. Source data

Here, we hypothesized that enhancing potential generated by individual MENPs would enable robust DBS at low concentrations of synthetic materials. To test this hypothesis, we synthesized anisotropic core–double-shell Fe_3_O_4_–CoFe_2_O_4_–BaTiO_3_ hexagonal nanodiscs (ME nanodiscs, MENDs) and tested their ability to mediate neuronal responses to MF stimuli (Fig. 1a). Through mechanistic studies of MEND-mediated neuronal depolarization, we derived design rules for ME neuromodulation in vivo and demonstrated wireless control of reward and motor behaviours in mice without transgenes at MEND concentrations 100 times lower than the nanoparticle concentrations used in prior ME neuromodulation studies.

## Results

### Synthesis and characterization of MENDs

MENDs were synthesized from magnetite nanodisc (MND) templates produced through reduction of hexagonal haematite (Fe_2_O_3_) (Methods, Fig. 1b and Supplementary Figs. 1 and 2). MNDs with a diameter of 231 ± 29 nm and thickness of 31 ± 9 nm were coated with a CoFe_2_O_4_ shell via an organometallic method, resulting in Fe_3_O_4_–CoFe_2_O_4_ core–shell nanodiscs (CFONDs, 240 ± 42 nm in diameter; Methods, Fig. 1c and Supplementary Figs. 2–5). Piezoelectric BaTiO_3_ shells were then grown onto the CFONDs yielding core–double-shell Fe_3_O_4_–CoFe_2_O_4_–BaTiO_3_ MENDs (250 ± 41 nm diameter; Methods, Fig. 1d and Supplementary Figs. 2, 4 and 5).

The addition of the CoFe_2_O_4_ shell was motivated by the greater magnetocrystalline anisotropy compared with Fe_3_O_4_ (250 ppm for CoFe_2_O_4_ versus 25 ppm for Fe_3_O_4_) and the magnetic interactions at the interface between the materials were expected to further increase the magnetostrictive response of the core–shell particles^16,25,26^. To test this, the ME coefficient (*α*_ME_) of MENDs was first numerically predicted and then determined experimentally (Fig. 1e and Supplementary Fig. 6).

Using micromagnetic simulation (Methods), the increase in shape anisotropy in CFONDs was predicted to yield 1,028 times enhancement in effective deformation over spherical nanoparticles (0.83026 ppm versus 0.000808 ppm) (Fig. 1f). Based on experimental observation, the CFONDs were assumed to be either in a magnetic vortex or an in-plane macrospin remnant state (Supplementary Fig. 7). The spring exchange coupling^16,25^ and surface magnetostriction^26^ at the Fe_3_O_4_ and CoFe_2_O_4_ interface in CFONDs yielded 43.7 times enhancement in effective deformation as compared with Fe_3_O_4_ MNDs with the same volume (0.01898 ppm; Fig. 1f). The micromagnetic simulation results were then introduced into a finite element model of the piezoelectric response of the BaTiO_3_ shell, demonstrating the scaling of the ME coefficient (*α*_ME_) with the magnetostrictive deformation (Fig. 1g and Supplementary Fig. 8).

ME coefficient was then experimentally determined through electrochemical means (Fig. 1e and Supplementary Fig. 6). The nanoparticles were simultaneously exposed to a constant offset MF (OMF), which magnetized them to near saturation where the change in slope of the magnetization as a function of field amplitude is maximized^27^, and an alternating MF (AMF), which drove magnetization change to induce magnetostriction. The peak *α*_ME_ of MENDs was measured to be 150 mV mT^−1^ cm^−1^, which is four times higher than that for isotropic CoFe_2_O_4_–BaTiO_3_ core–shell MENPs (Fig. 1h and Supplementary Fig. 9). The discrepancy between the simulated >1,000 times increase in the strain response (Fig. 1f) and the experimentally observed four times increase in *α*_ME_ can probably be attributed to the compensation of the electric polarization in a multi-domain piezoelectric shell (Supplementary Fig. 10) and the strain relaxation at the interface between the cubic CoFe_2_O_4_ and the tetragonal BaTiO_3_, which highlights the opportunity for further materials optimization.

The ME coefficient peaked at 220 mT of OMF, which is near the saturation field of the MENDs recorded via vibrating sample magnetometry^27^ (Supplementary Fig. 11). Fixing the amplitude of the OMF at 220 mT, the *α*_ME_ was then found to increase with increasing AMF frequency (Fig. 1i and Supplementary Fig. 12a), which is expected for frequencies below the piezoelectric resonance in the megahertz range^28–30^. The AMF amplitude did not have an impact on the *α*_ME_ (Fig. 1j and Supplementary Fig. 12b). The ME measurements were independent of the identity or concentration of ions in the electrolyte, as similar values of *α*_ME_ were found in normal and ten times concentrated phosphate-buffered saline (PBS) and in Tyrode’s solution (Supplementary Fig. 12c) despite the expected differences in voltammograms (Supplementary Fig. 12d).

### MEND-mediated neuromodulation in vitro

**Fig. 2:**
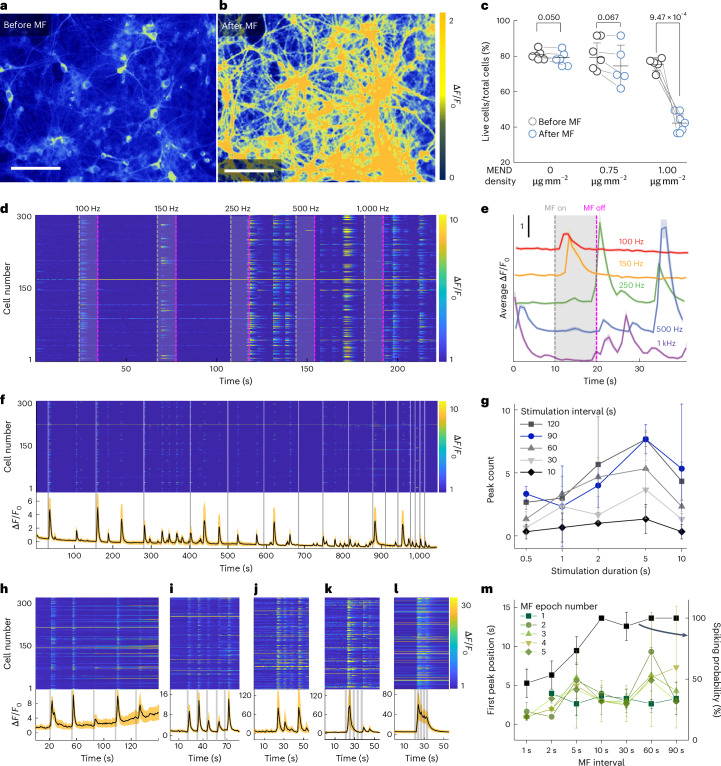
MEND-mediated neuronal stimulation in vitro. **a**,**b**, The relative GCaMP6s fluorescence change (∆*F*/*F*_0_) in hippocampal neurons decorated with MENDs before (**a**) and after (**b**) MF application (10 s, OMF 220 mT; AMF 1 kHz, 10 mT). Scale bars, 150 µm. **c**, The change in live cell ratio (counted from a live–dead assay in neurons normalized to the total number of cells marked by Hoechst staining) following three cycles of MF for neurons decorated with different MEND densities (0 µg mm^−2^, 0.75 µg mm^−2^, and 1 µg mm^−2^). Statistical significance was tested via one-way ANOVA and Tukey’s multiple comparison tests (*n* = 5 plates per condition, *P* = 3.79 × 10^–7^ for 1 µg mm^−2^; *P* = 0.79 for 0.75 µg mm^−2^; *P* = 0.998 for 0 µg mm^−2^; ****P ≤ 0.0001, n.s. *P* > 0.05). The error bars indicate s.d. **d**,**e**, Individual (**d**) and average (**e**) traces of GCaMP6s ∆*F*/*F*_0_ in 300 hippocampal neurons decorated with MENDs in response to 10 mT AMF with frequencies 100, 150, 250, 500 and 1,000 Hz (*H*_OMF_ = 220 mT). The dashed grey and magenta lines indicate the beginning and end of MF stimulation, respectively. **f**, Individual cell (top) and mean (bottom) GCaMP6s fluorescence changes in 300 neurons in response to 2 s MF epochs applied at varying intervals (OMF 220 mT; AMF 150 Hz, 10 mT). **g**, The number of GCaMP6s fluorescence peaks as a function of stimulation epoch length for rest intervals of 10, 30, 60, 90 and 120 s (OMF 220 mT; AMF 150 Hz, 10 mT). **h**–**l**, Individual cell (top) and mean (bottom) GCaMP6s fluorescence changes in response to 2 s MF (OMF 220 mT; AMF 100 Hz, 10 mT) epochs at 30 s (**h**), 10 s (**i**), 5 s (**j**), 2 s (**k**) and 1 s (**l**) intervals for 0.75 µg mm^−2^ MEND density. In **f** and **h**–**l** bottom panels, the lines and shaded areas represent the mean and s.e.m., respectively. **m**, The position of the first GCaMP6s peak from the MF onset and spiking probability equal to the fraction of trials triggering GCaMP6s transients across five MF epochs. The error bars indicate s.d. (*n* = 3 plates per condition). Source data

The efficacy of ME modulation was first assessed in primary hippocampal neurons expressing fluorescent calcium indicator GCaMP6s and decorated with MENDs at a density of 1 µg mm^−2^. The exposure to a single 10 s epoch of combined MF 220 mT OMF and 10 mT, 1 kHz AMF yielded a robust increase in GCaMP6s fluorescence (Fig. 2a,b, Supplementary Fig. 13 and Supplementary Video 1); however, the effect had a latency of 11.3 ± 4.0 s and diminished following three 10 s stimulation epochs separated by 30 s rest periods. The diminished response to the third MF epoch could be attributed to the excitotoxicity of the stimulation at 1 µg mm^−2^ MEND density (Fig. 2c and Supplementary Fig. 14). Reducing the MEND density to 0.75 µg mm^−2^ was sufficient to ensure neuronal viability indistinguishable from control cultures without MENDs, even after the MF application. The addition of MENDs to neurons had no effect on viability. These findings motivated the use of MEND concentrations ≤0.75 µg mm^−2^ to achieve repeatable and safe ME neuromodulation.

The latency of the ME stimulation was then interrogated as a function of AMF frequency. Although *α*_ME_ scales with the AMF frequency, resulting in larger fluctuations of the membrane potential, high-frequency (>150 Hz) stimulation yields neuronal conduction block^31,32^. Indeed, for AMF frequencies >150 Hz, the neurons appeared silenced during MF (220 mT OMF and 10 mT AMF) exposure, and rebound activity was observed upon MF removal (Fig. 2d,e, Supplementary Figs. 13 and 15, and Supplementary Videos 1 and 2). In contrast, at frequencies of 100 Hz and 150 Hz, GCaMP6s fluorescence transients emerged during the stimulation (Supplementary Fig. 15). At frequencies <100 Hz, the probability of observing GCaMP6s transients in response to MF stimuli was reduced (Supplementary Fig. 16), which could in part be due to the reduced *α*_ME_ (Fig. 1i). Therefore, 100 and 150 Hz were chosen as the AMF frequencies for the following experiments in vitro and in vivo.

We further assessed the role of stimulation duration and interval between field epochs. Stimulation epochs (220 mT OMF; 10 mT, 150 Hz AMF) longer than 2 s produced a greater number of GCaMP6s transients than 1 s and 0.5 s stimulation epochs regardless of the rest epoch length (Fig. 2f,g, Supplementary Fig. 16 and Supplementary Video 3). However, unlike short stimulation epochs that produced 2.0 ± 1.6 GCaMP6s fluorescence transients per epoch, longer stimulation epochs yielded unsynchronized responses (3.9 ± 3.09 GCaMP6s transients; Fig. 2g and Supplementary Fig. 16).

Fixing the duration of MF epoch to 2 s and reducing the AMF frequency to 100 Hz, we further investigated the influence of the inter-epoch interval (1 s, 2 s, 5 s, 10 s, 30 s, 60 s and 90 s) and the number of prior MF epochs on the latency and probability of neuronal response to stimulation (Fig. 2h–m and Supplementary Fig. 17). The number of prior stimulation epochs did not have a measurable effect on neuronal responsiveness or the average latency of 3.9 ± 2.9 s, regardless of the interval (Fig. 2m). In trials with inter-epoch intervals below the average latency (1 s and 2 s), the GCaMP6s transient triggered by a given MF epoch could appear during the subsequent epoch, manifesting in reduced latency and reduced spiking probability (Fig. 2m). The decrease in the spiking probability at inter-epoch intervals ≤5 s indicates the potential need for recovery time of ≥5 s to evoke synchronized MEND-mediated Ca^2+^ influxes in cultured neurons.

We also tested if the extent of neuronal response to the 2 s MF stimuli (220 mT OMF; 10 mT, 150 Hz AMF) scaled with the MEND density. At MEND densities of 0.75 µg mm^−2^, 0.5 µg mm^−2^ and 0.25 µg mm^−2^, 74.1 ± 23.6%, 46.3 ± 27.6% and 27.6 ± 23.3% of neurons were responsive to MF, respectively (Supplementary Fig. 18a–c). In the absence of MENDs, no notable response to identical MF conditions was observed (Supplementary Fig. 18d), which implies the combined quasi-magnetostatic field alone is not sufficient to generate inductive effects on neuronal activity.

The MF conditions employed for ME neuromodulation (220 mT OMF; 10 mT, 100–150 Hz AMF) did not evoke mechanical or thermal effects on neurons even in the presence of specialized mechano- and thermoreceptors owing to the inability of MNDs to oscillate in the presence of a near-saturation OMF or dissipate observable heat at AMF frequencies below the kilohertz range (Supplementary Fig. 19).

### Mechanistic investigation of MEND-mediated neuromodulation

**Fig. 3:**
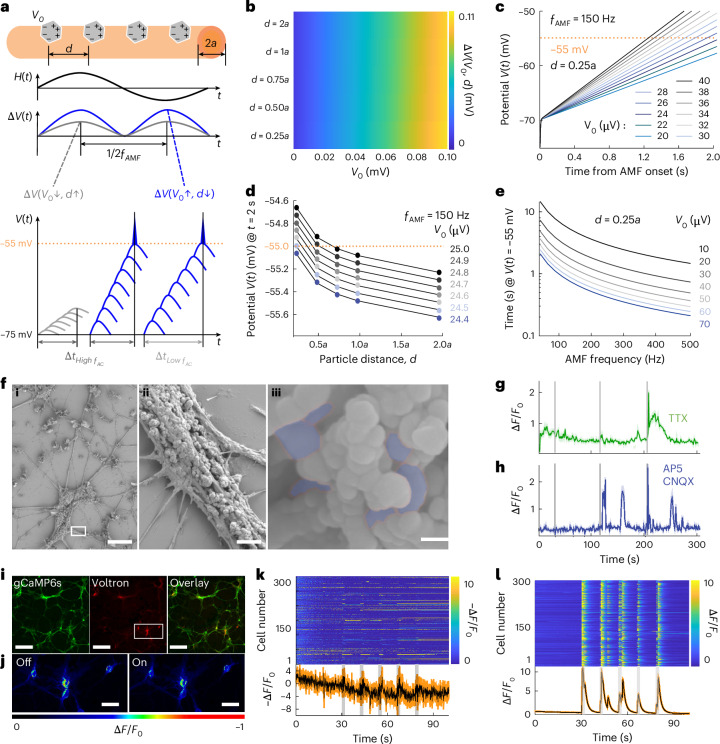
Mechanistic study of MEND-mediated neuromodulation. **a**, An illustration of stimulation mechanism, where *d* is spacing between MEND particles, $a$ is cell radius, $\Delta V$ is the change in membrane potential per half-period of an AMF, and ${V}_{0}$ is the voltage generated by a single MEND. **b**, The calculated $\Delta V$ as a function of ${V}_{0}$ and $d$. **c**, Simulated membrane potential $V(t)$ as a function of time from AMF onset for varying ${V}_{0}$ values and $d=0.25a$. The threshold for action potential firing, –55 mV, is indicated with a dashed line. **d**, $V(t)$ at a time *t* = 2 s after AMF onset as a function of $d$, for varying ${V}_{0}$ values. **e**, Time to reach threshold membrane potential (–55 mV) from the resting potential (–75 mV) as a function of AMF frequency ƒ_AMF_ for varying ${V}_{0}$ values. **f**, (i–iii) SEM images showing MENDs decorating cultured hippocampal neurons. (ii) A higher-magnification image of the area marked by a box in panel i. (iii) MENDs on the neuron surface shaded in blue. Scale bars: 20 µm (i), 5 µm (ii) and 100 nm (iii). **g**,**h**, GCaMP6s fluorescence change in neurons decorated with MEND following 2 s stimulations (OMF 220 mT; AMF 150 Hz, 10 mT, marked by vertical grey bars) in the presence of TTX, 1 µM (**g**) or a cocktail of AP5, 100 µM and CNQX, 20 µM (**h**). **i**, Fluorescent images of primary hippocampal neurons co-transfected with Voltron 2.0 (labelled with JF585) and GCaMP6s. Scale bars, 150 µm. **j**, The fluorescence change of JF585-labelled Voltron 2.0 in neurons decorated with MENDs before (left) and after (right) 2 s MF application (10 mT, 100 Hz AMF; 220 mT OMF). Scale bars, 40 µm. **k**,**l**, Individual (top) and average (bottom) traces of negative relative fluorescence change (−∆*F*/*F*_0_) of JF585-labelled Voltron 2.0 (**k**) and GCaMP6s ∆*F*/*F*_0_ traces from MEND-decorated neurons subjected to five 2 s epochs of combined MF (10 mT, 100 Hz AMF; 220 mT OMF) separated by 10 s intervals (**l**). In **g** and **h** and in bottom panels in **k** and **l**, the lines and shaded areas represent the mean and s.e.m. Source data

Despite their individual potentials of ~37.5 µV, MENDs were shown to mediate neuronal excitation, which typically requires membrane depolarization between 15–30 mV (ref. ^33^). We hypothesized that MENDs exert repetitive subthreshold stimulation on neuronal membranes, which is then integrated across multiple periods of the AMF, resulting in depolarization facilitation (Fig. 3a, Supplementary Note 1 and Supplementary Fig. 20).

The integrated change in the membrane potential over a single period of AMF, Δ*V*, scales linearly with a single MEND potential *V*_0_, while only modest increases are afforded by a reduction in the interparticle distance, *d* (Fig. 3b,c). The integration of the subthreshold events evoked by each half-period of AMF (Fig. 3a) then yields a monotonic increase of membrane potential over a stimulation epoch (Fig. 3c). Neuronal potential (initially set to −70 mV) can reach a threshold value of –55 mV in 2 s if the MENDs are spaced by 0.25*a*, where *a* is axonal radius, and each generate *V*_0_ > 24.5 µV on the membrane, which is consistent with our experimental observations (Fig. 2d–f). For MENDs with *V*_0_ < 24.5 µV, the threshold potential cannot be reached within 2 s regardless of *d* (Fig. 3d). The latency to reach the threshold potential could be further reduced by increasing *V*_0_ or AMF frequency (due to increased ${\alpha }_{{{\mathrm{ME}}}}$) (Fig. 3e). However, by design, our model did not recapitulate the neuronal conduction block at AMFs with frequencies >150 Hz, which contrasted with our experimental observations.

Our model also assumed a uniform distribution of MENDs and their direct attachment to neuronal membranes, given that MEND potentials could undergo ionic screening in physiological environments^34,35^. Scanning electron microscopy (SEM) imaging on the MEND-decorated neurons (Fig. 3f and Supplementary Figs. 21 and 22) revealed the presence of individual MENDs as well as few-particle aggregates on the cell membranes. The average interparticle distance was consistent with the estimation of neuronal and particle densities in our experiments (0.75 μg mm^−2^ of MENDs and 526 cells mm^−2^; Supplementary Note 2).

Neuronal depolarization was diminished, but not entirely eliminated, in the presence of the voltage-gated sodium channel blocker tetrodotoxin (TTX, 1 µM), as the threshold potential could be reached regardless of the state of the sodium channels (Fig. 3g, Supplementary Fig. 23a and Supplementary Video 4). Moreover, MEND-mediated excitation did not exclusively rely on synaptic transmission within the neuronal network, as GCaMP6s transients were observed in the presence of a cocktail of the α-amino-3-hydroxy-5-methyl-4-isoxazolepropionic acid receptor antagonist ((2*R*)-amino-5-phosphonovaleric acid (AP5), 100 µM) and the non-*N*-methyl-d-aspartate receptor antagonist (6-cyano-7-nitroquinoxaline-2,3-dione (CNQX), 20 µM) (Fig. 3h, Supplementary Fig. 23b and Supplementary Video 5). MEND-mediated GCaMP6s transients were also observed in low-density neuronal cultures (105 cells mm^−2^), a condition that mimicked neuronal isolation (Supplementary Fig. 24).

Membrane depolarization during MEND-mediated stimulation was further observed via fluorescence voltage imaging with Voltron 2.0 (Fig. 3i–k and Supplementary Fig. 25). In the absence of MENDs, MF application did not yield changes in Voltron 2.0 fluorescence (Supplementary Fig. 26). GCaMP6s imaging was also repeated in the same samples at a rate of 10 fps to verify that the imaged neurons remained viable (Fig. 3i,j). The latency from the MF onset for the GCaMP6s fluorescence maxima was found to be 857 ± 69 ms (Fig. 3k). Using a Ca^2+^ indicator with faster kinetics (GCaMP6f) delivered a similar latency of 827 ± 477 ms (Supplementary Fig. 27). At an imaging rate of 10 fps, the sum of the average and standard deviation of the latency is ≥1.5 s, which is consistent with the data acquired at 1 fps rate showing less reliable responses to MF epochs duration ≤2 s (Fig. 2f,g and Supplementary Fig. 16).

### MEND-mediated neuromodulation in vivo

**Fig. 4:**
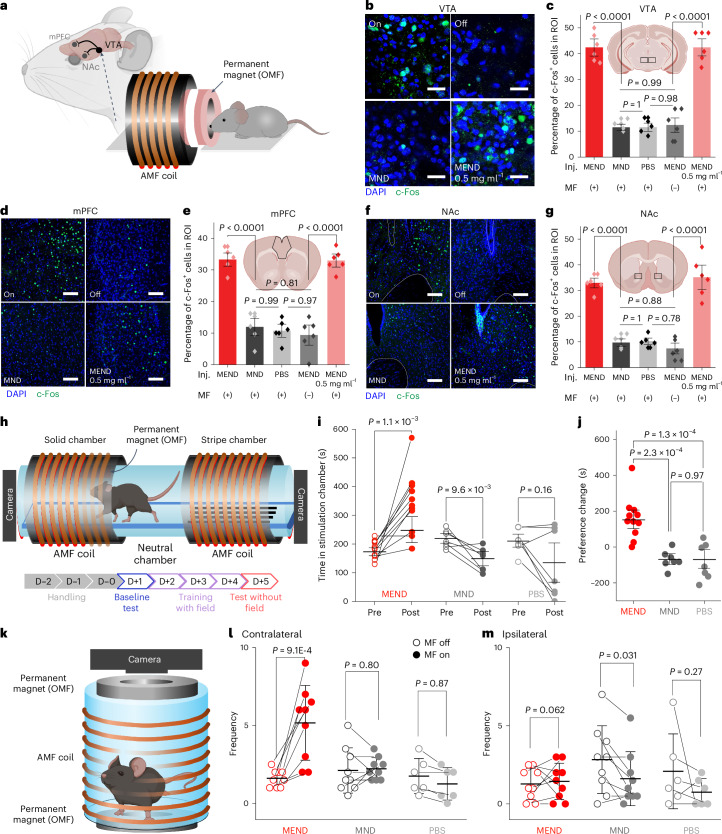
MEND-mediated neuronal stimulation in mice. **a**, A schematic of mice injected with MENDs in VTA and placed inside a permanent magnet field providing OMF and a surrounding solenoid providing AMF. **b**, Confocal images of c-Fos-expressing neurons among DAPI-marked cells in the VTA. Top left: MENDs (1.5 mg ml^−1^) with (+) magnetic stimulation. Top right: MEND particles (1.5 mg ml^−1^) without (−) magnetic stimulation. Bottom left: control MNDs (1.5 mg ml^−1^) + magnetic stimulation. Bottom right: MENDs (0.5 mg ml^−1^) + magnetic stimulation. Scale bars, 25 µm. **c**, Quantification of c-Fos-expressing neurons for the conditions shown in **b** and in the subjects injected with PBS and exposed to MF. **d**–**g**, Confocal images (**d** and **f**) and quantification (**e** and **g**) of c-Fos-expressing neurons in the mPFC (**d** and **e**) and NAc (**f** and **g**) for the same conditions as in **b** and **c**. Scale bars, 100 µm (**d** and **f**). In **c**, **e** and **g**, statistical significance was tested via one-way ANOVA and Tukey’s multiple comparison tests (*n* = 6). **h**, A schematic of the place preference arena (top) and experimental timeline (bottom). **i**, Time spent in the stimulation chamber out of a total assay time of 600 s, for pre-learning (day 1, open markers) and post-learning (day 5, solid markers). Paired *t*-test was performed for MEND (*n* = 11) and MND (*n* = 7) groups, and Wilcoxon signed-rank test was performed for PBS (*n* = 7) group because the data did not follow normal distribution. **j**, The change in time spent in the stimulation chamber between day 1 and day 5. *P* values were calculated by one-way ANOVA with Tukey’s post-hoc comparison test. **k**, A schematic illustration of the cylindrical arena. **l**,**m**, The numbers of contralateral (**l**) and ipsilateral (**m**) rotations during a baseline 3 min session (MF off) and during a 3 min stimulation assay consisting of 5 s MF epochs separated by 25 s intervals. Wilcoxon signed-rank test was performed to compare ipsilateral rotations of the MND group MF on and off. Other groups followed a normal distribution, and paired *t*-test was performed to calculate *P* values. In **c**, **e**, **g**, **i**, **j**, **l** and **m**, the lines and error bars indicate the mean and s.d. Inj., injection. Source data

We next evaluated whether MENDs could mediate ME neuromodulation in deep brain structures in mice (Fig. 4a). MENDs (1.5 µl volume, 1.5 mg ml^−1^ or a control solution) were injected into the left ventral tegmental area (VTA), a predominantly dopaminergic structure critical to reward processing^36,37^, in wild-type C57BL/6J (WT) mice. Following a 1-week recovery period, the percentage of activated neurons, as marked by expression of an immediate early gene *c-Fos*, was significantly higher in the VTA of mice injected with MENDs and exposed to the three cycles of MF in comparison with controls injected with PBS, MNDs without the piezoelectric BaTiO_3_ shell, or mice injected with MENDs and not exposed to the MF (Fig. 4b,c and Supplementary Fig. 28). The expression of c-Fos in response to MF stimulation was also significantly higher in the nucleus accumbens (NAc) and the medial prefrontal cortex (mPFC), the excitatory targets of the VTA^36,37^, in mice injected with MENDs than in controls (Fig. 4d–g and Supplementary Figs. 29 and 30). Notably, reducing the MEND concentration to values comparable to the experiments in vitro (1.5 µl at 0.5 mg ml^−1^), resulted in c-Fos expression levels in the VTA, NAc and mPFC similar to those found with higher particle concentrations (Fig. 4b–g and Supplementary Figs. 28–30).

Based on prior work on dopamine-dependent reward circuits^37^, we tested whether ME stimulation in the VTA could induce a conditioned place preference in a three-chamber assay in mice (Fig. 4h and Supplementary Fig. 31). During learning days 2–4, mice injected with MENDs in the VTA exhibited an increased tendency towards the magnetic stimulation chamber, as compared with the mice injected with control solutions (Supplementary Fig. 32 and Supplementary Video 6). On day 5 (test), mice injected with MENDs demonstrated a conditioned preference to the stimulation chamber (as compared with day 1, baseline), while mice injected with MNDs and PBS, respectively, exhibited or trended towards aversion for the stimulation chamber (Fig. 4i,j). This aversion could probably be attributed to the minor vibrations and noise associated with electromagnets operating at 150 Hz (refs. ^38,39^). These results show that MEND-based neuromodulation can affect reward behaviour in untethered mice without the need for transgenes.

To assess the broader potential of MEND-mediated neuromodulation, we applied this approach to DBS in the subthalamic nucleus (STN), a common clinical target for treatment of movement disorders^40^. As the unilateral STN DBS in rodents has been shown to induce rotational behaviour in cylindrical arenas compatible with MF coils^41^, we conducted a similar assay (Fig. 4k and Supplementary Fig. 33). MF exposure yielded increased contralateral rotations in mice injected with MENDs, while no significant differences in number of contralateral rotations were found in MND-injected or naive control groups (Fig. 4l and Supplementary Video 7). The MEND-mediated stimulation did not significantly increase the number of ipsilateral rotations (Fig. 4m and Supplementary Video 7). The behavioural observations were accompanied with significantly greater expression of c-Fos in the motor-control centres, red nucleus and primary motor cortex^42^ in the left hemisphere of the MEND-injected mice as compared with controls (Supplementary Fig. 34).

### Dynamics and longitudinal stability of MEND neuromodulation

**Fig. 5:**
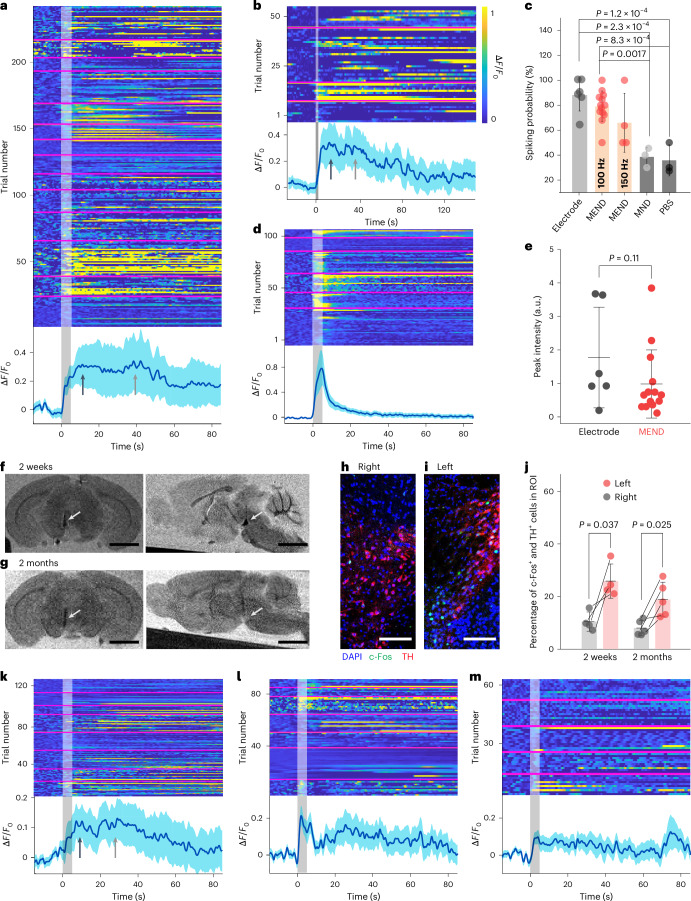
Biocompatibility and stability of MENDs. **a**,**b**, Fibre photometry recordings of GCaMP6s ∆*F*/*F*_0_ in the VTA of anaesthetized mice at 2 weeks following MEND injections in the same brain region with 5 s MF epochs of 100 Hz 10 mT AMF, 200 mT OMF (**a**) and 2 s MF epochs of 150 Hz 10 mT AMF, 200 mT OMF (**b**). **c**, The fraction of trials exhibiting a GCaMP6s fluorescence maximum (peak) within 20 s from the MF or current onset 2 weeks after the injection or implantation surgery. **d**, GCaMP6s ∆*F*/*F*_0_ recorded in mice implanted with a fibre and electrodes in the VTA and stimulated with 5 s current epochs. **e**, The mean value of GCaMP6s fluorescence peak per animal in **a** and **d**. The error bars represent s.d. **f**,**g**, MRI images (coronal view (left), scale bars 2 mm; sagittal view (right), scale bars 3 mm) of the brains isolated from mice at 2 weeks (**f**) and 2 months (**g**) following unilateral injections of MENDs into the left VTA. The arrows indicate the MEND bolus. **h**,**i**, Fluorescent images of right (**h**) and left (**i**) VTA immunostained for TH, c-Fos and DAPI. Scale bar, 100 µm. The area including MENDs in the left VTA is darker than the surroundings. **j**, The percentage of c-Fos-expressing cells among the TH-expressing cells in the left and right VTA at 2 weeks and 2 months following unilateral MEND injection. **k**–**m**, Fibre photometry traces in response to MF (5 s, OMF 220 mT, AMF 10 mT, 100 Hz) at 1 month (**k**), 2 months (**l**) and 3 months (**m**) following the MEND injection and fibre implantation surgery. In **a**, **b**, **d** and **k**–**m**, individual trial ∆*F*/*F*_0_ is shown (top) and the lines and shaded areas represent mean and s.e.m. across trials shown above (bottom) (**a**, *n* = 14; **b**, *n* = 4; **d**, *n* = 6; **k**, *n* = 8; **l**, *n* = 7; **m**, *n* = 5 mice). The grey rectangles indicate MF epochs, and the magenta horizontal lines delineate data from individual mice. In **c**, one-way ANOVA followed by Tukey’s post-hoc comparison test was applied for statistical analysis. *P* > 0.05 is not indicated. In **e**, two-sample *t*-test was performed, and in **j**, paired *t*-test was performed as the data are normally distributed. Source data

Neural activity during ME modulation in the VTA was recorded with fibre photometry (Supplementary Fig. 35). Following an adenoviral delivery and a 2-week incubation period (Methods), GCaMP6s expression was found in cell bodies in the VTA, and in axons projecting to NAc and mPFC (Supplementary Fig. 36). GCaMP6s fluorescence was then recorded in response to MF stimuli with AMF frequencies of 100 and 150 Hz (Fig. 5a,b), while OMF and AMF magnitudes were maintained at 220 mT and 10 mT, respectively. To reach a comparable stimulation dose, the MF epoch duration was set to 5 s for 100 Hz and 2 s for 150 Hz AMF conditions. In mice injected with MENDs GCaMP6s fluorescence increase was observed in 79.1 ± 12.3% of trials with 5 s epochs of 100 Hz AMF (14 mice, 267 trials; Fig. 5a,c) and in 65.9 ± 23.6% of trials with 2 s epochs of 150 Hz AMF (4 mice, 53 trials; Fig. 5b,c). A notably lower fraction of trials, commensurate with baseline activity, exhibited GCaMP6s fluorescence increase in mice injected with MNDs or PBS (Supplementary Fig. 37).

To compare the dynamics of MEND-mediated stimulation to electrical DBS (Fig. 5c–e), we applied sinusoidal currents ranging between 2 and 10 µA in amplitude with a frequency of 100 Hz via implanted electrodes (Supplementary Fig. 38 and Supplementary Note 3). These currents elicited responses in 87.9 ± 13.3% trials and, as expected, did not evoke extended increase in ∆*F*/*F*_0_ (Fig. 5c,d). These differences between MEND and electrode DBS probably stem from multiple factors. First, electrodes locally deliver current to neurons within their 100–200 µm vicinity^43^. MENDs can diffuse ~500 µm from the injection site (Supplementary Fig. 39) creating interfaces with a greater volume of cells but individually delivering weaker stimuli, which in turn may result in a slower return to baseline membrane potential via calcium-mediated repolarization mechanisms^44–46^. Second, MENDs may differentially affect non-neuronal cell populations in the brain, resulting in a sustained elevation of neuronal calcium. The examination of GCaMP6s dynamics for individual trials suggests the presence of primary and secondary ∆*F*/*F*_0_ transients (Methods and Supplementary Figs. 40 and 41). While the primary transient could be attributed to neuronal excitation, the secondary transient decays over ~80 s and could potentially be due to MENDs effects on glia and their subsequent influence on neurons^46–49^. Our preliminary examination in neuronal cultures without glial suppression indicates the presence of GCaMP6s responses with delayed kinetics, supporting the idea that glia contribute to extended MEND-mediated stimulation (Supplementary Fig. 42).

To assess the longitudinal stability of MEND-mediated neuromodulation, enhanced T2-weighted contrast in magnetic resonance imaging (MRI) in the VTA was observed 2 weeks following unilateral MEND injection (1.5 µl, 1.5 mg ml^−1^) and appeared somewhat diminished at 2 months (Fig. 5f,g and Supplementary Fig. 43). The MENDs mediated neuronal excitation with MF (220 mT OMF, 10 mT 100 Hz AMF) as marked by c-Fos expression 2 weeks and, to a reduced degree, 2 months post-injection. Notably, consistent with broad MEND targeting, c-Fos expression was observed in both dopamine neurons and non-dopamine cells (Fig. 5h–j and Supplementary Fig. 44). The MF activation of the VTA dopamine neurons 2 weeks and 2 months following MEND delivery was accompanied with increased c-Fos expression in mPFC and NAc (Supplementary Fig. 45).

The probabilities of observing MF-evoked GCaMP6s transients in the VTA were 68.3 ± 18.4%, 66.3 ± 21.2% and 59.8 ± 25.8% at 4 weeks, 2 months and 3 months following MEND injection, respectively (Fig. 5k–m). The reduction in efficacy of MEND-mediated neuromodulation over time was probably due to diffusion and cellular uptake of MENDs, rather than differential dissolution of MENDs (Supplementary Fig. 46). Transmission electron microscopy (TEM) images of the brain slices prepared 2 weeks following MEND injection indicate that the particles could be endocytosed (Supplementary Fig. 47).

Finally, we assessed the biocompatibility of MEND-mediated neuromodulation. We quantified microglial activation marker (ionized calcium-binding adaptor molecule 1, Iba1), astrocytic glial fibrillary acidic protein (GFAP) and macrophage activation marker (cluster of differentiation 68, CD68) surrounding MENDs relative to PBS injection controls and to 300 µm stainless-steel microwire implants commonly used for electrical neuromodulation in mice. We observed negligible increases in Iba1, GFAP and CD68 expression in mice injected with MENDs compared with those injected with PBS. The expression of these markers also did not increase significantly with MF exposure. By contrast, all markers were significantly upregulated near the microwire implants relative to MEND injections (Supplementary Figs. 48 and 49). Notably, in mice injected unilaterally with MENDs, expression of Iba1 and CD68 near the injection site was not significantly different from the opposite (naive) hemisphere 2 months post-surgery (Supplementary Fig. 50).

## Conclusion

We colloidally synthesized anisotropic MENDs with a core–double-shell architecture (Fe_3_O_4_–CoFe_2_O_4_–BaTiO_3_) that exhibit an enhanced ME coefficient as compared with previously reported isotropic CoFe_2_O_4_–BaTiO_3_ nanoparticles. When decorated onto neuronal membranes in vitro at densities 0.5–1 µg mm^−2^, MENDs mediated robust and rapid excitation with combined OMF (220 mT) and AMF (10 mT) at frequencies 100–150 Hz.

To investigate biophysical mechanisms underlying ME neuromodulation, we proposed a model of repeated subthreshold depolarization, where voltages generated by individual MENDs could be integrated over AMF periods and across multiple particles. Although our model offered one potential mechanism of MEND-mediated neural excitation, it probably underestimated the magnitude of the effect as it did not consider the spatial summation of the membrane potential fluctuations^50^ or the diffuse-charge accumulation of ions around MENDs^51,52^. In addition, our model did not consider the current injection from the MENDs. Including the kinetics and distribution of specific ion channels, neuronal geometry, external excitatory and inhibitory inputs, and the effects of the MEND polarization on the surrounding ions will most likely yield a closer quantitative agreement with experiments.

Despite being qualitative, our model suggested routes for optimizing MEND-mediated neuromodulation. It indicated that the potentials produced by individual MENDs have a stronger effect on overall depolarization as compared with the particle density. Therefore, the development of materials with higher ME coefficients is necessary to achieve the shorter stimulation latencies commensurate with millisecond neuronal dynamics.

Based on insights from our numerical and in vitro studies, MEND-mediated stimulation was deployed in the deep brain of genetically intact mice where it achieved remote neuronal excitation at particle concentrations as low as 0.5 mg ml^−1^ (1.5 µl injections). MEND-mediated neural excitation in the VTA enabled wireless magnetic control of reward behaviour. Similarly, unilateral ME neuromodulation in the STN drove rotational behaviour akin to electrical stimulation, indicating the future utility of this approach as an alternative to DBS with implanted electrodes. This approach could potentially be scaled to translational animal models or human patients by employing helmet-style rather than arena-integrated magnetic instrumentation (Supplementary Fig. 51).

We anticipate that the materials and biophysical insights developed in this work will set the stage for future innovation in multiferroic nanomaterials and their applications in fundamental and translational neuroscience.

## Methods

### Materials and methods

#### Synthesis of MENDs

Synthetic procedures for MENDs were reproduced across two institutions (Massachusetts Institute of Technology and Friedrich-Alexander University of Erlangen–Nuremberg). The Fe_3_O_4_ MNDs were synthesized by reducing haematite nanodiscs synthesized via previously established protocols^11,53,54^. Haematite nanodiscs were first produced by heating a uniform mixture of 0.273 g of FeCl_3_·6H_2_O (Fluka), 10 ml ethanol and 600 μl of deionized (DI) water in a sealed Teflon-lined steel vessel at 180 °C for 18 h. After washing the red haematite nanodiscs with DI water and ethanol three to five times, the dried haematite was dispersed in 20 ml of trioctyl-amine (Sigma-Aldrich) and 1 g of oleic acid (Alfa Aesar/Thermo Fisher Scientific). For the reduction of haematite to magnetite, the mixture was transferred into a three-neck flask connected to a Schlenk line, evacuated for 20 min at room temperature and then heated to 370 °C (20 °C min^−1^) in H_2_ (5%) and N_2_ (95%) atmosphere for 30 min.

The CFONDs were formed by nucleation and growth of a CoFe_2_O_4_ layer on the surface of MNDs. For this procedure, 120 mg of MNDs (cores) were dispersed uniformly in a precursor solution of 20 ml diphenyl ether (Aldrich), 1.90 ml oleic acid (Sigma-Aldrich), 1.97 ml oleylamine (Aldrich), 257 mg cobalt acetylacetonate (Co(acac)_2_, Aldrich) and 706 mg iron acetylacetonate (Fe(acac)_3_, Aldrich). A three-neck flask including the solution of MND cores and the shell precursors was connected to a Schlenk line. The solution was evacuated and then heated to 100 °C (7 °C min^−1^) for 30 min in an N_2_ atmosphere while magnetically stirring at 400 rpm. After closing the N_2_ line, the temperature was increased to 200 °C (7 °C min^−1^), maintained for 30 min and then increased to 230 °C (7 °C min^−1^) and maintained for 30 min. The solution was cooled to room temperature (~30 min), and the resulting CFONDs were washed with ethanol and *n*-hexane and subjected to centrifugation at 6,869 rcf for 8 min; the washing process was repeated two to three times. The thickness of the CoFe_2_O_4_ layer is controlled by repeating the organometallic synthesis and washing steps described above. To obtain a 5 nm CoFe_2_O_4_ layer, the synthesis was repeated three times.

The Fe_3_O_4_–CoFe_2_O_4_–BaTiO_3_ MENDs were made by formation of BaTiO_3_ shell on the surface of CFOND via the sol–gel method. A mixture comprising 16 mg of CFONDs dispersed in *n*-hexane, 30 ml of DI water, 6 ml of ethanol and 2 g of poly(vinylpyrrolidone) (Sigma-Aldrich) was sonicated for 20 min, which led to segregation of the oil phase. The oil phase and other insoluble solids were removed with a spatula. The hydrophilic CFOND dispersions were then transferred to a three-neck flask connected to a Schlenk line, and then dried in vacuum at 80 °C until amber-coloured gel was formed on the bottom of the flask. The gel was redispersed in the BaTiO_3_ shell precursor solution that was prepared by mixing 0.5 g citric acid (Sigma-Aldrich) and 24 µl titanium isopropoxide (Aldrich) dissolved in 15 ml of ethanol and 0.1 g citric acid and 0.0158 g barium carbonate (Aldrich) dissolved in DI water. The solution of CFONDs and BaTiO_3_ precursors were moved to the three-neck flask connected to the vacuum line and kept at 80 °C for 12–14 h. The powders were then moved to a clean ceramic container and heated at 600 °C for 2 h, 700 °C for 2 h, then 800 °C for 1 h, sequentially. To prevent breaking the BaTiO_3_ shell, the furnace door was kept closed until the temperature slowly cooled down to room temperature. The MENDs were dispersed in Tyrode and PBS before being used for in vitro and in vivo experiments.

#### Structural and magnetic characterization of magnetic nanomaterials

Structural imaging of MNDs, CFONDs and MENDs and energy-dispersive X-ray spectroscopy mapping on MEND-including neurons was performed via SEM (Zeiss Merlin). TEM imaging and single-particle electron diffraction analysis was performed using a FEI Tecnai G2 Spirit TWIN TEM. The diameter and thickness of MNDs, CFONDs and MENDs were estimated from the ensemble averages of particles in TEM and SEM images. Powder X-ray diffraction patterns of as-synthesized MNDs, CFONDs and MENDs were collected by a three-circle diffractometer coupled to a Bruker-AXS Smart Apex charge-coupled device detector with graphite-monochromated Mo K_α_ radiation ($\lambda =0.71073\,{\text{\AA }}$), and the data were processed with PANalytical HighScore Plus software. Room-temperature hysteresis curves were generated using the combined superconducting quantum interference device and vibrating sample magnetometer mode of a Quantum Design MPMS-3 at 300 K. An Agilent 5100 inductively coupled plasma-optical emission spectrometer was used to quantify the elemental concentration for the calculation of saturation magnetization. For inductively coupled plasma-optical emission spectrometry analysis, nanoparticles were digested in 37% v/v HCL (Sigma-Aldrich) overnight and diluted in 2 wt% HNO_3_ (Sigma-Aldrich).

#### Micromagnetic simulations

Full magnetoelastic simulations were performed with MuMax3 magnetoelastic extension using literature values of magnetic and elastic constants for the materials (Supplementary Table 1) and a cell size of 2 × 2 × 2 nm^3^. The simulation was performed at discrete MFs corresponding to the maxima of an oscillating field with an amplitude of 10 mT around an offset at 220 mT. Increasing the strength of the elastic damping constant, a steady-state elastic displacement could be achieved within a simulation time of 72–168 h running on a GeForce RTX 3060 GPU. The steady state emerged after ~35 ns of simulation time following the initial field application, and 10 ns was required for subsequent field steps. Hence, all simulations were performed with the initial field value for 40 ns, and each subsequent field value for 15 ns. To quantify the normalized displacement of each particle at the maxima of an oscillating field with an amplitude of 10 mT around an offset at 220 mT, we averaged root-mean-square displacement normalized to the solid diagonal of each simulation cell. To calculate the deformation of each particle under the oscillating field with an amplitude of 10 mT around an offset at 220 mT, we averaged the difference in normalized displacements at two sequent maxima. Note that, due to limits of MuMax3 Magnetoelastic extension, we had to use the same sign in the anisotropy of the Fe_3_O_4_ and CoFe_2_O_4_ for the core–shell particles. This is an acceptable approximation as a lower bound of the strain in the composite particle. With a difference in anisotropy sign, the very large magnetostriction could not be simulated with our existing computational infrastructure as the resultant pressure waves took microseconds to dissipate.

#### Design and fabrication of electromagnets

For ME coupling coefficient measurements and Ca imaging in vitro, TEMCo 14 AWG copper magnet wire was wound around a C-shaped magnetic core with an 8 mm gap. The coil was connected to a power supply (Crown DC-300A Series II) and signal generator (Picoscope 2204A) to generate a MF combining a static OMF (magnitude 0–320 mT) and an AMF (frequency 0–1,000 Hz, amplitude 0–14 mT). For in vivo experiments, we separated the apparatuses that generated the OMF and AMF to generate a uniform, time-varying, MF over a larger volume. To generate the AMF (10 mT, 150 Hz) for c-Fos expression and fibre photometry experiments, we used a TEMCo 14 AWG copper magnet wire solenoid with a 10 cm inner diameter connected to the power supply and the signal generator. To generate the d.c. MF (220 mT), we used a ring-shaped permanent magnet (3.81 cm outer diameter × 1.905 cm inner diameter × 0.3175 cm thick, K&J Magnetics, RX8C2) that was positioned in the centre of the AMF coil. To apply MF during behaviour experiments, we designed a custom arena composed of two-connected cylindrical chambers (FixtureDisplays clear acrylic tube, 75 mm diameter, 2 mm wall thickness and 205 mm length) that was divided into two stimulation chambers (11 cm) separated by a neutral area (19 cm) that contained the animal entry port. The entry port was covered with 3D-printed acrylic curved plate after the entrance of mouse. To generate OMF during behavioural experiments, two rectangular permanent magnets (7.62 cm × 7.62 cm × 1.27 cm thick, K&J Magnetics, BZ0Z08-N52) were placed at the top and bottom of each stimulation chamber. To generate AMFs during behavioural experiments, solenoids (TEMCo 14 AWG copper magnet wire) were wound around each stimulation chamber and connected to the power supply and the signal generator.

#### ME coupling coefficient measurements

To experimentally evaluate ${\alpha }_{{{\mathrm{ME}}}}$ of the MENDs, we expanded on the prior work that leveraged electrochemical means to determine ME coupling from nanoparticles dispersed in an electrolyte^55^ and applied this approach to MEND films^56–59^. A three-electrode electrochemical cell was used to measure the potential required to maintain the surface charge, which fluctuated according to the electric polarization of the MENDs in the presence of a MF. To fit within the 8 mm gap of the C-shaped electromagnet described above, a nuclear magnetic resonance tube (8 mm outer diameter, 0.5 mm thickness) was used as the electrochemical cell. Within the cell, the Ag/AgCl reference electrode, Pt counter electrode and working electrode were immersed in Tyrode’s solution, PBS 1× or PBS× 10. The working electrode was prepared by drop-casting MEND solution onto a 0.3 × 1.2 cm^2^ indium tin oxide glass substrate and connecting it to a copper wire using conductive silver epoxy (MG Chemicals, 842AR-15ML). The electrical connection was then sealed with epoxy (H.B. Fuller, 10010217) for insulation. The thickness of the MEND film on the indium tin oxide substrate was measured via a profilometer (Bruker Dektak DXT-A Stylus). All electrochemical measurements were performed using a potentiostat (Gamry Interface 1010E).

#### Fluorescent imaging in cultured hippocampal neurons

All animal procedures were approved by the Massachusetts Institute of Technology Committee on Animal Care (protocol #2305000529). Hippocampal neurons were extracted from neonatal rat (Sprague-Dawley, 001) pups (P1) and dissociated with Papain (Worthington Biochemical). The cells were then seeded on glass slides (5 mm diameter, Bellco Glass 1943-00005) in 24-well plates at a density of 112,500 cells ml^−1^. Before seeding, the glass slides were cleaned by evaporating ethanol with an alcohol lamp and then coated with Matrigel (Corning). The cells were maintained in 1 ml Neurobasal medium (Invitrogen). Glial inhibition was conducted with 5-fluoro-2′-deoxyuridine (F0503 Sigma) 3 days after seeding; this step was omitted for experiments evaluating the effects of glia on neuronal responses to MEND-mediated modulation. Four days following seeding, the neurons were transduced with 1 µl of an adeno-associated virus serotype 9 (AAV9) carrying a fluorescent calcium ion indicator GCaMP6s under a pan-neuronal human synapsin (hSyn) promoter (AAV9-hSyn::GCaMP6s, Addgene viral prep #100843-AAV9, >1 × 10^13^ IU ml^−1^). For simultaneous voltage and calcium imaging, AAV9-CAG::Voltron2, home-packaged, >5 × 10^11^ IU ml^−1^) and AAV9-CAG::GCaMP6s, (Addgene viral prep #100844-AAV9, >1 × 10^13^ IU ml^−1^). After 5 days of incubation, calcium (Ca^2+^) imaging with GCaMP6s was performed at 1 fps imaging speed. Voltage imaging with Voltron 2.0^60,61^ combined with Janelia Fluor 585 (Promega HT1040) was performed at 10 fps; these experiments were followed by GCaMP6s imaging of the same region and with the same frame speed. To confirm the latency using Ca^2+^ indicator with faster kinetics, calcium imaging at 10 fps was performed again with pAAV-Syn::GCaMP6f (Addgene viral prep #100837-AAV9, >1 × 10^13^ IU ml^−1^)^62^

ME neuromodulation with MENDs was expected to be most effective when the particles were in direct contact with neuronal membranes. Consequently, all experiments in vitro were conducted following a 1 h incubation period to allow the particles to precipitate onto the cells. The MEND density on the cell membranes was measured by quantifying their mass on each sample and calculating the area occupied by the neuronal network. For Ca^2+^ imaging, the hippocampal neurons were washed once with Tyrode’s solution and then immersed into 0.1 mg ml^−1^ MEND solution within a well of a 24-well plate for 1 h to obtain ~0.75 g_MEND_ mm^−2^ density of the particles on the neuronal surfaces. To change the MEND density on neuronal surfaces, we varied the concentration and volume of the MEND incubation solution. The hippocampal neurons on the glass coverslips were then transferred into a custom sample holder containing 200 µl Tyrode’s solution, which was introduced into an electromagnet as described above. The fluorescence changes in GCaMP6s were recorded at a rate of 1 fps. The fluorescence intensity of each cell was analysed with ImageJ software, and *F*_0_ values were determined as the average fluorescence intensity during 30 s before the initial application of the AMF. Averaged Δ*F*/*F*_0_ was estimated from 300 randomly selected neurons from 3 plates. The number of the peaks in $\Delta$F/F_0_ was counted when the peak height is larger than the half of mean standard deviation of Δ*F*/*F*_0_ for the overall imaging time of each video. The cells were defined as responsive if the Δ*F*/*F*_0_ value exceeded three times the standard deviation (3*σ*) of the baseline (collected over 30 s before the initial application of the AMF) within 15 s from MF onset; hence, the responsiveness was defined as the fraction of responsive cells per MF epoch. The ratio of MF epoch number inducing average Δ*F*/*F*_0_ ≥ 3*σ* of average Δ*F*/*F*_0_ baseline to the overall number of MF epoch application in an individual video was defined to be the spiking probability. The latency was defined to be the time taken from the MF onset to the highest intensity point of the spikes.

To examine the cell viability in the presence of MENDs following MF stimuli, we performed analysis with a Live/Dead Viability/Cytotoxicity Kit (Invitrogen, L3224). The live and dead cells were indicated with Calcein AM (green) and ethidium homodimer-1 (red). The proportion of viable cells was calculated by normalizing the viable cell number to the total number of cell nuclei based on Hoechst staining (Thermo Scientific, Hoechst 33342 Solution (20 mM)). The neurons were incubated with the assay reagents for 20 min in Tyrode’s solution at 37 °C and then imaged before and after three cycles of MF field via a fluorescent microscope (Olympus IX73, 20× objective lens).

#### Stereotactic surgeries

All animal procedures were approved by the Massachusetts Institute of Technology Committee on Animal Care (protocol #2208000413). Surgeries were conducted under aseptic conditions within a stereotaxic frame (David Kopf Instruments) with 6–8-week-old WT mice. Approximately equal numbers of male and female mice were used (c-Fos expression analysis: three females and three males in each group; place preference assays: five females and six males for MENDs, four females and three males for MNDs, three females and four males for PBS; cylinder test: five females and four males for the MEND and MND groups, three females and three males for the PBS group; for photometry, Fig. 5a, seven females and seven males, Fig. 5b, two females and two males, Fig. 5d, three females and three males, Fig. 5k, four females and four males, Fig. 5l, four females and three males, Fig. 5m, four females and one male). Mice were anaesthetized under isoflurane (0.5–2.5% in O_2_) using an anaesthesia machine (VET EQUIP). During the surgery, the eyes were covered with ophthalmic ointment, and a heat pad was used to maintain the animals’ core temperature. Mice were provided with subcutaneous injections of 0.6 ml of sterile Ringer’s solution for hydration and extended-release buprenorphine (1 mg kg^−1^) for analgesia at the start of the procedure. The head was fixed into position using ear bars, then the fur on the top of the head was removed using depilatory cream. Following sterilization of the skin with betadine and ethanol, a midline incision was made along the scalp. Coordinates for the injection/implantation site were established on the basis of the Mouse Brain Atlas^63^. A small craniotomy was drilled through the skull using a rotary tool (Dremel Micro 8050) and a carbon steel burr (Heisinger, 19007-05), and the dura was gently removed. Particles were injected into the VTA (anterior–posterior (AP) −2.9 mm, medial–lateral (ML) −0.5 mm, dorsal–ventral (DV) −4.5 mm) or STN (AP −2.06 mm, ML −1.50 mm, DV −4.50) using a microinjection apparatus (10 µl Hamilton syringe #80308, UMP-3 syringe pump and Micro4 controller; all from World Precision Instruments).

For c-Fos expression analysis, behavioural assays, MRI imaging and TEM analysis, 1.5 µl of MEND particles, MND control particles or PBS were injected at a rate of 500 nl min^−1^. For MENDs and MND nanoparticle solutions, we used 1.5 mg ml^−1^ concentration for all experiments, except for an additional 0.5 mg ml^−1^ low concentration of MENDs tested in the c-Fos expression experiments. For PBS controls, the same volume of sterile PBS was injected instead of particles. After injection, the syringe was lifted up by 0.1 mm from the initial DV coordinate and left in place for 10 min before slowly withdrawing (0.5 mm min^−1^), and then the skin was sutured (Ethicon, Ethilon 661H, polyamide 6, 19 mm needle length).

For the mice used for fibre photometry experiments, 300 nl of AAV9 hSyn::GCaMP6s, Addgene viral prep #100843-AAV9, >1 × 10^13^ IU µl^−1^) was injected along with the 1.3 µl particles or PBS. The AAV solution was loaded into the syringe tip after the particles or control PBS, such that it was injected first and immediately followed by the particle or PBS injection into the brain. Following the injection, the syringes were lifted as described above. At 0.1 mm above the injection coordinates, a 200-µm-diameter silica fibre (Thorlabs FT200EMT) with a 2.5-mm-diameter stainless-steel ferrule (Thorlabs SF230-10) or the new fibre described in the following section was implanted and cemented in place with adhesive acrylic (C&B-Metabond, Parkell) followed with dental cement (Jet Set-4).

Following surgery, a subcutaneous injection of carprofen (5 mg kg^−1^) was provided as an anti-inflammatory and analgesia agent, and the animals were placed in a clean recovery cage, part of which was positioned on a heating pad with ad libitum access to water and diet gel wet food.

#### Fibre photometry

Fibre photometry recordings were performed using a Neurophotometrics fibre photometry system (FP3002, Neurophotometrics). This system utilized a blue (peak wavelength *λ* = 470 nm) light-emitting device (LED) to excite GCaMP6s and a violet (peak *λ* = 415 nm) LED as an isosbestic wavelength control, with a fluorescence light path that includes a dichroic mirror to pass emitted green fluorescence (passband 495–530 nm) onto a complementary metal-oxide semiconductor camera (FLIR BlackFly). The 470 nm and 415 nm LEDs were each calibrated to provide 75 µW of optical power out of the tip of a 200 µm silica fibre matching the type implanted into the animals (Thorlabs FT200EMT). The system was coupled to a low-autofluorescence branching bundle patch cord (400 µm core, 0.57 numerical aperture, Doric), which was connected to the animal’s implant using a ceramic split mating sleeve (Thorlabs ADAF1). All photometry recordings were performed under isoflurane anaesthesia (0.5–2.5% in O_2_, VET EQUIP) using a nose cone. After induction, the patch cord was connected to the animal’s fibre implant, and the animal’s head was placed in the centre of the custom magnetic apparatus described above: 10-cm-diameter solenoid AMF coil with a ring-shaped static magnet inside to provide OMF, whose centres are aligned. Fibre photometry recordings were performed at 130 Hz sampling rate.

After recording a 5 min baseline to account for rapid photobleaching at the start of the experiment, each animal received 10–20 total pulses of magnetic stimulation. Data were collected in Bonsai software and exported to MATLAB (MathworksR2022a) for analysis. Photometry data were analysed as follows: data were low-pass filtered below 25 Hz (second-order Butterworth filter), both isosbestic (405 nm excitation wavelength) and Ca^2+^-sensitive (470 nm excitation wavelength) signals were fitted with MATLAB exp2 fitting function, and then the fitting functions were subtracted from the original signals to correct for photobleaching. To remove the motion artifacts, the baseline-corrected isosbestic signal was subtracted from the baseline-corrected 470 nm signal. For the experiments using 2 s pulses of MF (OMF 220 mT, AMF 150 Hz, 10 mT), a 200 µm silica fibre was implanted in the mice and MF epochs were applied every 180s. The fluorescence average of 30 s before every stimulation epoch was taken as *F*_0_, and Δ*F*/*F*_0_ was segmented corresponding to each stimulation epoch (30 s pre-stimulation and 150 s post-stimulation).

Experiments comparing MEND-mediated stimulation and electrode DBS as well as repeated recordings 2 weeks, 4 weeks, 2 months and 3 months after the surgery were performed with the fibre described in Supplementary Note 3 and Supplementary Fig. 38. The *F*_0_ was calculated from 15 s before stimulation, and Δ*F*/*F*_0_ was segmented corresponding to each stimulation epoch (15 s pre-stimulation and 85 s post-stimulation). In these experiments, the DBS current was between 2 and 10 μA with a frequency of 100 Hz. MF stimuli consisted of 5 s epochs of OMF 220 mT combined with AMF at 100 Hz, 10 mT. For all GCaMP6s traces, we quantified spiking probability, peak intensity, peak width and peak position. When a MF or DBS triggered ∆*F*/*F*_0_ ≥ 2*σ* (where *σ* is a standard deviation of baseline) within 20 s from the onset of the stimulation, the trial was classified as responsive. The fraction of responsive trials in each animal was defined as spiking probability. The peak intensity and position refer the maximum value of ∆*F*/*F*_0_ transient and its position with respect to stimulation onset, respectively. The peak width is the interval between the times when the ∆*F*/*F*_0_ = *σ*/2 before and after the maximum.

#### Immunohistochemical quantification of biomarkers

For the c-Fos expression experiments and biocompatibility assessment, mice were anaesthetized via an intraperitoneal injection of ketamine (100 mg kg^−1^) and xylazine (10 mg kg^−1^) mixture. The heads of the anaesthetized mice were placed into the centre of a custom-built apparatus with a 10-cm-diameter solenoid a.c. coil outside and ring-shaped static magnet inside, whose centres are aligned. Each mouse received three 2 s pulses of magnetic stimulation (220 mT OMF; 150 Hz, 10 mT AMF) separated by 90 min rest epochs before killing. Following the 90 min c-Fos induction period, the anaesthetized mice were killed by a lethal intraperitoneal injection of a sodium pentobarbital (Fatal-plus, 50 mg ml^−1^, dose 100 mg kg^−1^). The mice were then transcardially perfused with PBS and 4% paraformaldehyde, and their brains were extracted and kept in 4% paraformaldehyde overnight at 4 °C. After moving the fixed tissue to PBS and storing at 4 °C for 24 h, the brains were sectioned into 50 µm coronal slices with vibrating blade microtome (Leica VT1000S). The permeabilization was done on the slices for 30 min in the dark at room temperature in a 0.3% v/v Triton X-100 solution in PBS, and then the blocking was done for 1 h with 0.3% v/v Triton X-100 and normal donkey serum 5% v/v blocking serum solution in PBS with an orbital shaker. Following PBS washing three times, the brain slices were incubated in the first antibody solution overnight at 4 °C. After three washes with PBS, the brain slices were immersed in a secondary antibody solution for 2 h at room temperature on the orbital shaker in a dark room. After another three washes with PBS, the brain slices were stained with 4′,6-diamidino-2-phenylindole (DAPI), washed and then transferred onto glass slides with mounting medium (Fluoromount G, Southern Biotech). Rabbit anti-c-Fos (1:500, Cell Signaling Technology, 2250s) primary antibodies and donkey anti-rabbit Alexa Fluor 488 (1:1,000, Invitrogen, A-21206) secondary antibodies were used for the c-Fos expression analysis. For tyrosine hydroxylase (TH) staining, sheep-anti-tyrosine hydroxylase antibody (1:500, Novus Biologicals NB300-110SS) and donkey anti-sheep IgG (H + L), Alexa Fluor 568 (1:1,000, Thermo Scientific A-21099) were used as primary and secondary antibodies. Three pairs of primary (goat anti-Iba1 antibody (1:500, Abcam ab107159), goat anti-GFAP antibody (1:1,000, Abcam ab53554), rabbit anti-CD68 antibody (1:250, Abcam ab125212)) and secondary (donkey anti-goat IgG (H + L), Alexa Fluor 633 (1:1,000, Fisher Scientific A-21082) and donkey anti-rabbit Alexa Fluor 488 (1:1,000, Invitrogen, A-21206)) antibodies were used for the toxicity assessment.

#### Behavioural assays

For behavioural experiments, the mice were tested during the light phase of the 12 h light/dark cycle.

For the place preference assay, a 71-mm-inner-diameter acrylic cylinder was divided into three chambers: two 11-cm-long stimulation chambers (solid and stripe-patterned bottom) separated by a 19-cm-long neutral chamber (purple-coloured bottom). The stimulation chambers were equipped with custom-made electromagnets and static magnets outside the stimulation chambers, as shown in Fig. 4h and Supplementary Fig. 30. For 3 days (day −2 to day 0; Fig. 4h and Supplementary Fig. 30) before the baseline place preference test, the mice were habituated daily for 15 min in the setup and to the researchers. On day 1, the mice were placed into the chamber for 5 min habituation during which they could explore the chambers freely without any stimulation, and their place preference was tested for the following 10 min without any magnetic stimulation. From day 2 to day 4, following a 5 min habituation in the setup, the mice were stimulated with the MF (220 mT OMF, 10 mT, 150 Hz AMF, 2 s epochs separated by 90 s rest periods) when they entered the less preferred area for 10 min daily. On test day 5, the mice explored the entire arena in the absence of MF stimuli in either chamber. The mouse location in the arena was recorded with two cameras (Logitech HD Pro Webcam C920) positioned at the ends of each stimulation chamber and Logitech Capture Bio Recording and Streaming Software (2.08.11) was used to record the videos. Analysis of the place preference assay was done manually, where the observer was blinded to the subject type and marked the time spent in each chamber. Mice that showed more than 500 s (total test time is 600 s) preference to a chamber during pre-test or stayed 0 s at the stimulated chamber on day 2 were eliminated from the subsequent analyses.

For the rotational behaviour assay, an acrylic cylinder with an inner diameter of 71 mm and a height of 8 cm height was used as an arena. The AMF was applied via by the custom-made electromagnet surrounding the chamber, and the OMF was provided by two O-shaped permanent magnets (7.5 mm outer diameter, 4 mm inner diameter) attached to the top and bottom of the cylinder (Supplementary Fig. 33). A 3 min baseline video was first acquired. Then, another video was recorded over a 3 min period, during which AMF was applied in 5 s epochs separated by 25 s intervals. The videos were scored manually by a researcher blinded to subject group, and the number of rotations was compared with the baseline.

#### MRI

MRI was performed on a 7 T magnetic resonance imager operated by Bruker AV4 NeoBioSpec70-20USR console, equipped with a 114 mm 660 mT m^−1^ actively shielded gradient and a QSN075/040 RF coil (Bruker BioSpin). T2-weighted images were obtained using the TurboRARE protocol with (Repetition Timer)/(Time to Echo) = 3,400/35 ms, echo spacing 11.667 ms, number of averages 4 and RARE factor 8. Both axial and coronal datasets were obtained with the geometric parameters of 196 × 196 matrix, field of view (FOV) of 18 mm × 18 mm, and interleaved slice thickness of 0.3 mm with no gap. The number of slices was adjusted to ensure the entire sample was covered.

#### Statistical analyses

OriginPro 2019 was used for assessing the statistical significance of all comparisons in this study except the post-hoc analysis for non-parametric data, which was performed with Matlab2023b. Although sample sizes were not determined with the power analysis, the group sizes for immunohistochemistry and behaviour tests were decided to be similar with previous research performed in the same brain circuit. Shapiro–Wilk test was performed to test normality of data distribution. For c-Fos expression analysis, the data distribution was tested for normality, and then analysed with analysis of variance (ANOVA) followed by Tukey’s post-hoc comparison test (*P < 0.05, **P < 0.01, ***P < 0.001). For the immunohistochemistry analyses relevant to the toxicity assessment, unpaired *t*-test was used to assess the differences between two groups, where significance threshold was indicated with non-significant (n.s.) *P* > 0.05, *P < 0.05, **P < 0.01, ***P < 0.001. In comparisons between c-Fos, Iba1, CD68 and TH expression in right and left hemispheres, paired *t*-test and Wilcoxon signed-rank test were used for parametric and non-parametric datasets, respectively. For place preference behavioural experiments, a Kruskal–Wallis test followed by a Tukey’s post-hoc comparison test was applied to compare three groups simultaneously with thresholds of n.s. *P* > 0.05, *P < 0.05, **P < 0.01, ***P < 0.001, ****P < 0.0001. For the comparison of pre- and post-learning days within each group, a paired *t*-test was performed when the data distribution was found normal. For non-normal distribution, Wilcoxon signed-rank test was performed. For the preference change data comparing all three groups, the data followed normality and, thus, one-way ANOVA was used followed by Tukey’s post-hoc comparison test. The same method was applied to compare the number of the ipsilateral and contralateral rotations in 3 min assays with and without MF.

For comparison of spiking probability in the photometry across mice injected with MENDs, MNDs, PBS or implanted with electrodes, one-way ANOVA followed by Tukey’s post-hoc comparison test employed applied to compare five groups simultaneously (***P ≤ 0.001, **P ≤ 0.01, *P ≤ 0.05, *P* > 0.05 is not indicated). For the peak intensity comparison between the MEND and electrode stimulations, a two-sample *t*-test for MEND data was performed. The peak width and position in the MEND group did not follow normal distribution, and a Mann–Whitney test was performed. The comparison of biomarkers in the brains of mice subjected to MF stimulation 2 weeks and 2 months after the MEND injections was performed via paired *t*-test as all data followed the normal distribution. ***P ≤ 0.001, **P ≤ 0.01, *P ≤ 0.05, n.s. *P* > 0.05.

### Reporting summary

Further information on research design is available in the Nature Portfolio Reporting Summary linked to this article.

## Online content

Any methods, additional references, Nature Portfolio reporting summaries, source data, extended data, supplementary information, acknowledgements, peer review information; details of author contributions and competing interests; and statements of data and code availability are available at 10.1038/s41565-024-01798-9.

## Supplementary information

Supplementary Information Supplementary Notes 1–4, Supplementary Tables 1–4, Supplementary Figs. 1–51, captions for Supplementary Videos 1–7 and Supplementary References.

Reporting Summary

Supplementary Video 1 GCaMP6s fluorescence changes (10× speed) in primary hippocampal neurons decorated with MENDs in response to 10 s pulses of combined OMF 220 mT and AMF 10 mT, 150 Hz.

Supplementary Video 2 GCaMP6s fluorescence changes (real time) in primary hippocampal neurons decorated with MENDs in response to 10 s MF epochs with the variation in the AMF frequency, while maintaining its amplitude (10 mT) and the magnitude of OMF (220 mT).

Supplementary Video 3 GCaMP6s fluorescence changes (real time) in primary hippocampal neurons decorated with MENDs in response to 2 s epochs of combined 220 mT OMF and 10 mT, 150 Hz AMF. Separation between stimulation epochs was 120 s, 90 s, 60 s, 30 s and 10 s, and each stimulation sequence was repeated three times.

Supplementary Video 4 GCaMP6s fluorescence changes (real time) in primary hippocampal neurons decorated with MENDs in response to 2 s epochs of 220 mT OMF and 10 mT, 150 Hz AMF in the presence of 1 µM TTX.

Supplementary Video 5 GCaMP6s fluorescence changes (real time) in primary hippocampal neurons decorated with MENDs in response to 2 s epochs of 220 mT OMF and 10 mT, 150 Hz AMF in the presence of 20 µM CNQX and 100 µM AP5.

Supplementary Video 6 Representative videos of a place preference assay with mice injected with MENDs and MNDs (1.5 µl at 1.5 mg ml^–1^ unilaterally in the left VTA) recorded from the side of the arena opposite to the stimulation chamber (220 mT OMF and 10 mT, 150 Hz AMF).

Supplementary Video 7 Representative 3 min videos of the cylinder test for a naive mouse and a mouse unilaterally injected with MENDs in the left STN (1.5 µl at 1.5 mg ml^–1^) during MF stimulation (5 s epochs, 25 s intervals, 220 mT OMF and 10 mT, 150 Hz AMF).

## Source data

Source Data Fig. 1 Statistical source data.

Source Data Fig. 2 Statistical source data.

Source Data Fig. 3 Statistical source data.

Source Data Fig. 4 Statistical source data.

Source Data Fig. 5 Statistical source data.

## Data Availability

All of the data supporting this study are available via the Figshare public repository at 10.6084/m9.figshare.26771680 (ref. ^64^). Source data are provided with this paper.

## Code availability

The code for neuronal simulation and fluorescence image analysis are available via the Figshare public repository at 10.6084/m9.figshare.26778310 (ref. ^65^).
