# Supplementary material for: Magnetoelectric nanodiscs enable wireless transgene-free neuromodulation
Source: Nat Nanotechnol. 2024 Oct 11;20(1):121–31. doi: 10.1038/s41565-024-01798-9 (PMC11750723; doi:10.1038/s41565-024-01798-9)
Supplement: Supplementary file 1 — Supplementary Notes 1–4, Supplementary Tables 1–4, Supplementary Figs. 1–51, captions for Supplementary Videos 1–7 and Supplementary References. [file 41565_2024_1798_MOESM1_ESM.pdf]

nature nanotechnology

Supplementary information https://doi.org/10.1038/s41565-024-01798-9

Magnetoelectric nanodiscs enable wireless transgene-free neuromodulation

In the format provided by the authors and unedited

Table of Contents

Supplementary Table 1………………………………………………………………... p. 2
Supplementary Table 2………………………………………………………………... p. 6
Supplementary Table 3………………………………………………………………... p. 6
Supplementary Table 4………………………………………………………………... p. 19
Supplementary Note 1……………………………………………………………… p. 3
Supplementary Note 2……………………………………………………………… p. 7
Supplementary Note 3……………………………………………………………… p. 9
Supplementary Note 4……………………………………………………………… p. 10
Supplementary Figures 1 – 51………………………………………………... p.11-56
Captions for Supplementary Videos 1-7…………………………………………… p. 57
Supplementary References………………………………………………………… p. 58

Supplementary Table 1. Materials parameters employed in micromagnetic simulations of magnetostriction.

**Constant**	**Fe_3O_4**	**$CoFe_2O_4$**
Saturation Magnetization	480×10^3 A/m [19,20]	445×10^3 A/m [21]
Exchange Coupling Strength	1.3×10^{-12} J/m [22]	1.7×10^{-11} J/m [21]
Interlayer exchange coupling	1×10^{-12} J/m to Co [23]	1×10^{-12} J/m to Fe [23]
Crystalline Anisotropy Axis	(1, 1, 1) [22]	(1, 1, 1) [24]
Crystalline Anisotropy constant	13500 J/m^3 [22,25]	240000 J/m^3 [21,25]
Density	5110 kg/m^3	5230 kg/m^3
Magnetoelastic coupling constant	1.6×10^7 J/m^3 [22]	3.2×10^7 J/m^3 [26]
C11, C12, C44	240×10^9, 135×10^9, 87×10^9 N/m^2 [22]	244×10^9, 142×10^9, 75×10^9 N/m^2 [26]

Supplementary Note 1

The magnetoelectric coefficient, α_{ME}, of the core-double shell Fe_3O_4-$CoFe_2O_4$-$BaTiO_3$ magnetoelectric nanodiscs (MENDs) was measured to be 150 mV mT^{-1} cm^{-1} at the following magnetic field (MF) conditions: offset magnetic field (OMF) 220 mT and alternating magnetic field (AMF) with a frequency f_{AC}=150 Hz and amplitude 10 mT (**Fig. 1h-j**). Using the particle diameter of 250 nm and the AMF magnitude of 10 mT, the potential generated by an individual MEND at these MF conditions can be calculated as $\frac{150\ mV}{mT \cdot cm} \times 250\ nm\ \times 10\ mT\ = 37.5\ \mu V$, which is significantly below the threshold for neuronal activation and action potential firing (15-30 mV). To investigate the mechanism underlying the MEND-mediated neuronal modulation we developed a model based on: (1) the instantaneous change in membrane potential, ΔV, for each pulse of the AMF resulting from the integration of voltages generated by individual MENDs distributed with a spacing d on the cell membrane and (2) the dynamic change in membrane potential $V(t)$ resulting from the temporal summation of ΔV across subsequent periods of the AMF.

To model our system, we leverage the solutions to the three-dimensional cable equation, where neuron processes are approximated as cylindrical bodies, with radius a[2]. This framework enables the calculation of the change in membrane potential in the presence of multiple point sources of current or voltage (e.g. microelectrodes, or in our case MENDs) distributed across the membrane surface. As the classic cable model is limited to two microelectrodes, we have expanded it to include a larger number of sources for the membrane potential fluctuations. This allowed us to calculate the change in membrane potential ΔV as a function of the distance between MENDs, d, and the potential generated by a single MEND, V_0.

We modeled a neuron receiving stimulation via MENDs on its membrane as a cylindrical cell receiving current injection from a series of microelectrodes with spacing d and zero circumferential angle ($\theta = 0$) aligned along the cell surface (**Supplementary Fig. 16**). For two microelectrodes separated by the distance d that supply current i_0, the change in membrane potential, ΔV, according to the steady state solution of the cable model is[2]:

$$\Delta V(d, i_0) = 0.5 r_i i_0 a\ (L(d) + S(d)) \qquad Eq.1$$

Where r_i is the resistance of a unit length of the interior of the cell against longitudinal current flow, a is the radius of the cell, and L and S represent the spatial decay of potential caused, respectively, by the one-dimensional[2]. $L(d)$ is defined as:

$$L(d) = \frac{\lambda}{a} e^{-d/\lambda} \qquad Eq.2$$

Where λ is the neuron length constant, defined as the length over which the membrane potential decays to V_0/e . Values of $S(d)$ have been empirically determined, and we use values from the literature tabulated for varying values of d, shown in Supplementary Table 2[3].

MENDs on the neuron membrane can be represented as multiple microelectrodes spaced at a distance d, which we assume to be much smaller than the overall length of the neuron, l. Thus, the change in membrane potential during one stimulation pulse integrated across all microelectrodes becomes:

$$\Delta V(d, i_0) = \left| \int_d^l \frac{dV}{dx} dx \right| = |V(l) - V(d)| \\ = |0.5 r_i i_0 a\, (L(l) - L(d) + S(l) - S(d))| \qquad Eq.3$$

In the approximation that the potential change at a large distance from the point source approaches zero, we can assume that $L(l) \approx 0$ and $S(l) \approx 0$, which leads to a simplification of Eq. 3 to:

$$\Delta V(d, i_0) \approx |0.5 r_i i_0 a(-L(d) - S(d))| \qquad Eq.4$$

Given that MEND-mediated modulation effectively relies on voltage application rather than current injection, we applied a steady-state solution from the cable model to calculate current i_0 from the MEND potential V_0[4]:

$$i_o = \frac{2V_0}{r_i \lambda} \qquad Eq.5$$

where λ is the neuron length constant (defined above) determined by r_i and the membrane resistance per unit length. Hence, the integrated membrane potential change for a single pulse of the AMF can be rewritten in terms of d and V_0 as:

$$\Delta V(d, V_0) = |0.5 r_i i_0 a(-L(d) - S(d))| = \left| 0.5 r_i \frac{2V_0}{r_i \lambda} a \left[-\frac{\lambda}{a} e^{-\frac{d}{\lambda}} - S(d) \right] \right| \qquad Eq.6$$

The values for a, λ, and r_i taken from the literature are tabulated in Supplementary Table 3[3]. This equation implies that the total change in membrane potential $\Delta V(d, V_0)$ increases with decreasing spacing between the individual particles, d, and with increasing voltage generated from individual particles, V_0.

Using these insights about membrane potential change stemming from the integrated inputs from spatially distributed MENDs, we develop a framework to determine how the membrane potential varies in time across multiple cycles of AMF. Application of AMF to a neuron decorated with MENDs is anticipated to yield temporal summation of the subthreshold potentials generated by the MENDs during each half-period of the AMF. (Note that since the ME effect does not depend on the AMF sign, the frequency of voltage fluctuations in MENDs is $2 \times f_{AC}$, where f_{AC} is the AMF frequency). This temporal summation of a series of subthreshold potentials is known as neuronal facilitation[5]. Note that the assumption of neuronal facilitation is motivated by the similarity between the AMF half-period (3.33 ms at 150 Hz, 5 ms at 100 Hz) and the neuronal time constant (1-20 ms)[6,7].

To simulate neuronal facilitation during magnetoelectric stimulation with MENDs, we adopt a stochastic model of the repetitive activity of neurons[3], and we combine this with the three-dimensional cable model of the voltage distribution derived above (Eq. 6). When a neuron is subjected to a series of potential pulses with a given time interval between them, the membrane potential, $V(t)$, is the sum of two components: $N(t)$, a noise term that accounts for stochastic fluctuations in membrane potential, and $D(t)$, a function describing the reestablishment of resting membrane potential following each applied potential pulse. Since $N(t)$ is a noise term with a mean of 0 mV, on average, $V(t)$ reaches the threshold for neuron spiking when $D(t)$ does so. $D(t)$ is dependent on the state of the membrane potential at the onset of the applied potential pulse, and it is defined as:

$$D(t) = D_F + (D_I - D_F)e^{\frac{-(t-R)}{k}} \qquad Eq.7$$

Where D_I is the membrane potential at the onset of the applied potential, D_F is the membrane potential in the fully-recovered state dependent on the level of excitatory input, R is the absolute refractory period of the neuron spiking (we use $R = 0.7\ s$), and k is the time constant of decay of the after-potential (we fix $k = 9\ ms$ in this simulation). If we consider the sub-threshold voltage

delivered by MENDs to be akin to excitatory input to the neuron, then D_F depends on $\Delta V(d, V_0)$, as defined in Eq. 6. D_I varies depending on history, and it is defined as one-half of the difference between –90 mV and the value of $D(t)$ at the onset of the applied potential [3]. Thus, for each period of the applied AMF, $D_F' = D_{F,initial} + \Delta V$ and $D_I' = (D_F' - 90\ mV)\ /\ 2$, with these values getting updated for every cycle of the AMF.

According to this model, subthreshold potentials of the MENDs give rise to a potential change $\Delta V(d, V_0)$ for each period of the AMF, which decays according to $D(t)$ (Eq. 7). With an applied AMF of 150 Hz, subsequent voltage impulses delivered by the MENDs summate over time until the critical threshold is crossed and the neuron fires. Using this model, we find that neurons with a resting membrane potential of –70 mV decorated with MENDs and exposed to 150 Hz AMF will be depolarized to the critical threshold for action potential firing, –55 mV, after ≥ 2s, for a single MEND potential $V_0 > 24\ \mu V$ when inter-MEND spacing d is $0.25a$ (**Fig. 3e-g**).

Supplementary Table 2. $S(d)$ at different values of MEND spacing d in fractions of the axonal radius a.[3]

$\boldsymbol{d}$	$\boldsymbol{S(d)}$
0.25a	3.202
0.5a	1.212
0.75a	0.598
1a	0.327
2a	0.042

Supplementary Table 3. Parameters used to model MEND-mediated neuronal excitation with AMF.

**Parameter**	**Value**
Axial resistivity, R_i	150 Ωcm [8]
Axial resistance, r_i	$R_i/\pi a^2$
Axonal radius, a	2 µm [9]
Length constant, λ	258 µm [10,11]

Supplementary Note 2

The number of MENDs on the neurons was estimated by normalizing the cultured primary hippocampal neurons' density on the coverslips used for fluorescent imaging with GCaMP6s, GCaMP6f, and Voltron 2.0. As the cultured primary hippocampal neurons do not divide[12], we assumed the density of neurons per unit area (1 mm^2) to be constant and determined during seeding. We used 526 cells mm^{-2} as a commonly employed density in primary hippocampal neuronal cultures.

When 0.75 μg mm^{-2} of MENDs were introduced into a culture well and allowed to settle to the surface, on average each neuron became decorated with 1.43 ng (= 0.75 μg mm^{-2}/526 cells mm^{-2}) of MENDs. Dividing this number by the mass of a single MEND (M_{MEND}), we obtained an average number of MENDs on neuronal surfaces. M_{MEND} was calculated by assuming MNDs, CFONDs, and MENDs to possess the respective diameters of 230, 240, and 250 nm and thicknesses of 30, 40, and 50 nm. As MEND consists of a MND (Fe_3O_4) core and $CoFe_2O_4$ - $BaTiO_3$ double shell, the mass MEND one particle is the sum of mass of the core and the two shells ($M_{Fe_3O_4}, M_{CoFe_2O_4}, M_{BaTiO_3}$):

$$M_{Fe_3O_4} = V_{MND} \cdot \rho_{Fe_3O_4} = 1.37 \times 10^6\ nm^3 \times 5.17 \times 10^{-21}\ g\ nm^{-3} = 7.10 \times 10^{-15}\ g$$

$$M_{CoFe_2O_4} = (V_{CFOND} - V_{MND}) \cdot \rho_{CoFe_2O_4} = (2.00 - 1.37) \times 10^6\ nm^3 \times 5.29 \times 10^{-21}\ g\ nm^{-3}$$

$$= 3.33 \times 10^{-15}\ g$$

$$M_{BaTiO_3} = (V_{MEND} - V_{CFOND}) \cdot \rho_{BaTiO_3} = (2.71 - 2.00) \times 10^6\ nm^3 \times 6.02 \times 10^{-21}\ g\ nm^{-3}$$

$$= 4.27 \times 10^{-15}\ g$$

$$M_{MEND} = M_{Fe_3O_4} + M_{CoFe_2O_4} + M_{BaTiO_3} = 1.47 \times 10^{-14}\ g = 1.47 \times 10^{-5}\ \text{ng}$$

Based on this calculation, on average every neuron is decorated with ~9.73×10^4 number of MENDs. This allows for estimation of the average distance between MENDs: the MEND density 0.75 μg mm^{-2} translates into 5.10×10^7 MENDs per 1 mm^2 (0.75 μg mm^{-2} / 7.34×10^{-6} ng), which yields 140 nm ($1/\sqrt{5.10 \times 10^7 mm^{-2}}$) average distance between MENDs.

Based on the MEND size and a calculated average inter-particle distance, we estimated upper boundary of current that could be injected by an individual MENDs. Particles under time-

varying field are expected to generate current proportional to the rate of changing electric potential: $j = c \times dV/dt$ (j is current, c is the capacitance per unit area, V is potential, t is time). We assumed the maximum MEND potential of 35 μV, the rise time for 100 Hz is 2.5 ms (1/4 period), and the MEND capacitance of 2 μF cm^{-2} as determined by the outermost $BaTiO_3$ shell[13]. Then the upper boundary of MEND-generated current is $j \times (MEND\ surface\ area) = 28\ \mathrm{nA} \cdot \mathrm{cm}^{-2} \times \left(\frac{125}{\sqrt{3}}\ nm \times 125\ nm \times 6 \times 2 + \frac{125}{\sqrt{3}}\ nm \times 50\ nm \times 6\right) = 3.64 \times 10^{-8} nA$. Given that this current is orders of magnitude below the currents used for electrical stimulation (50 - 5000 μA)[14-17] we did not include current effects in our model.

Supplementary Note 3

Fabrication of a Custom Fiber for Electrical Stimulation and Fiber Photometry

The fiber for simultaneous photometry and electrical stimulation was produced through thermal drawing. Two polycarbonate (PC) layers (McMaster, 8574K45) were machined using a CNC mill. The first layer had dimensions of 10.7 × 16.7 mm^2 and a 9.5 × 11.9 mm^2 channel for placing an optical fiber post-draw. The second layer had dimensions of 6 × 16.7 mm^2 and two 4.7 × 4.7 mm^2 channels for converging wires during the draw. The PC layers were consolidated with a hot press at 185 °C and 5 psi for 60 minutes, using Teflon spacers to keep the channels open. The optical fiber channel was filled with styrene-ethylene-butadiene-styrene (SEBS) to maintain the geometry of the channel during the drawing process. SEBS pellets (Kraton, G1657) were molded using an 11 × 11.9 mm^2 aluminum channel in a vacuum oven set to 200 °C for 10 hours under continuous vacuum. The resulting SEBS slab was placed into the optical fiber channel of the PC preform and consolidated using a hot press at 130 °C and 1.5 psi for 15 minutes, using Teflon spacers to hold open the wire convergence channels. After the spacers were removed, the fiber was drawn in a vertical tower with a 3-zone furnace to attain 45-55 ratio of size reduction. A stable draw was achieved with temperatures of 140, 260, and 80 °C for the top, middle, and bottom furnaces, respectively. During the draw, spooled 100 µm stainless steel (SS) wires (Amazon, B0CB6C1X5Y) were fed into preform and converged into the fiber. The drawn fiber was cut into sections, electrical connections were made to the SS wires, and a silica optical fiber (Thorlabs, FT200UMT) was added. The SEBS in the channel was manually removed under a stereoscope, and on one end of the fiber, the polycarbonate was dissolved in dichloromethane to expose the SS wires. The wires were soldered onto copper traces connected to male header pins, and the fiber and header pins were epoxied onto a 3D-printed holder. The silica optical fiber was inserted into a steel ferrule (Thorlabs, SF230-10) with an ultra-violet curable epoxy (NOA 61, Norland Products), polished, and then inserted into the channel of the PC fiber and affixed with same optical epoxy. The ferrule was then glued into the 3D-printed holder used for implantation.

Supplementary Note 4

Electrical Stimulation Using an Implanted Fiber

To compare the dynamics of MEND-mediated stimulation to DBS via implanted electrodes, a group of mice (n=5) was implanted with custom fibers (Supplementary Note 3) comprising a 200 μm silica fiber and two 100 μm stainless steel electrodes within a polycarbonate body (Fig. S38). To generate $\Delta F/F_0$ GCaMP6s signals comparable to those observed with MEND-mediated stimulation, we applied sinusoidal currents ranging between 2-10 μA in amplitude with a frequency of 100 Hz, which are significantly lower than those used in therapeutic applications (50 - 5000 μA, 100-1000 Hz)[14-17].

Supplementary Figures

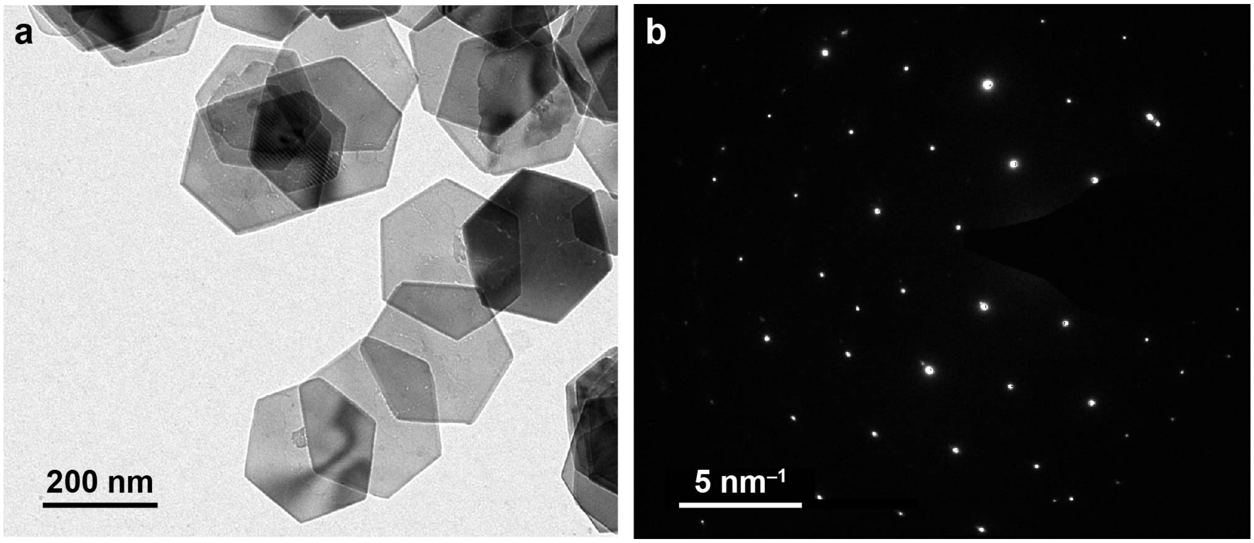

Supplementary Fig. 1 | Image of hematite nanodiscs. a, Transmission electron microscopy (TEM) image of an ensemble of hematite (Fe_2O_3) nanodiscs produced via a hydrothermal process prior to reduction. **b,** An electron diffraction pattern of an area on a single hematite nanodisc.

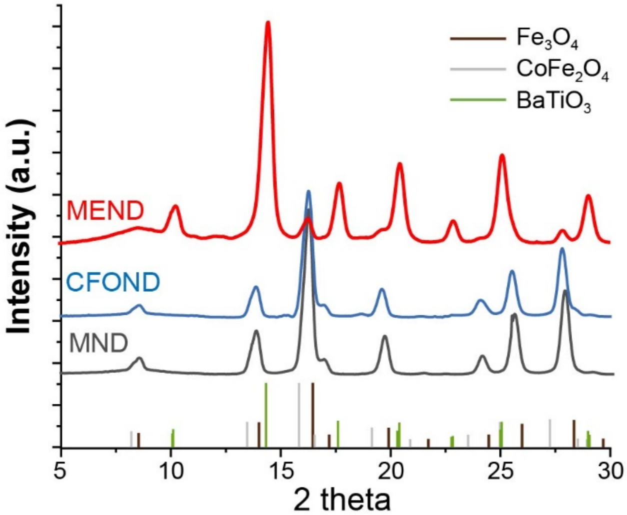

Supplementary Fig. 2| X-ray diffraction analysis. X-ray diffraction spectra of Fe_3O_4 magnetic nanodiscs (MNDs, grey), Fe_3O_4-$CoFe_2O_4$ core-shell nanodiscs (CFONDs, blue), and co-double shell magnetoelectric Fe_3O_4-$CoFe_2O_4$-$BaTiO_3$ nanodiscs (MENDs, red) shown together with the reference spectra for Fe_3O_4 (brown, cubic inverse spinel structure), $CoFe_2O_4$ (grey, cubic inverse spinel structure) and $BaTiO_3$ (green, tetragonal crystal system with perovskite structure).

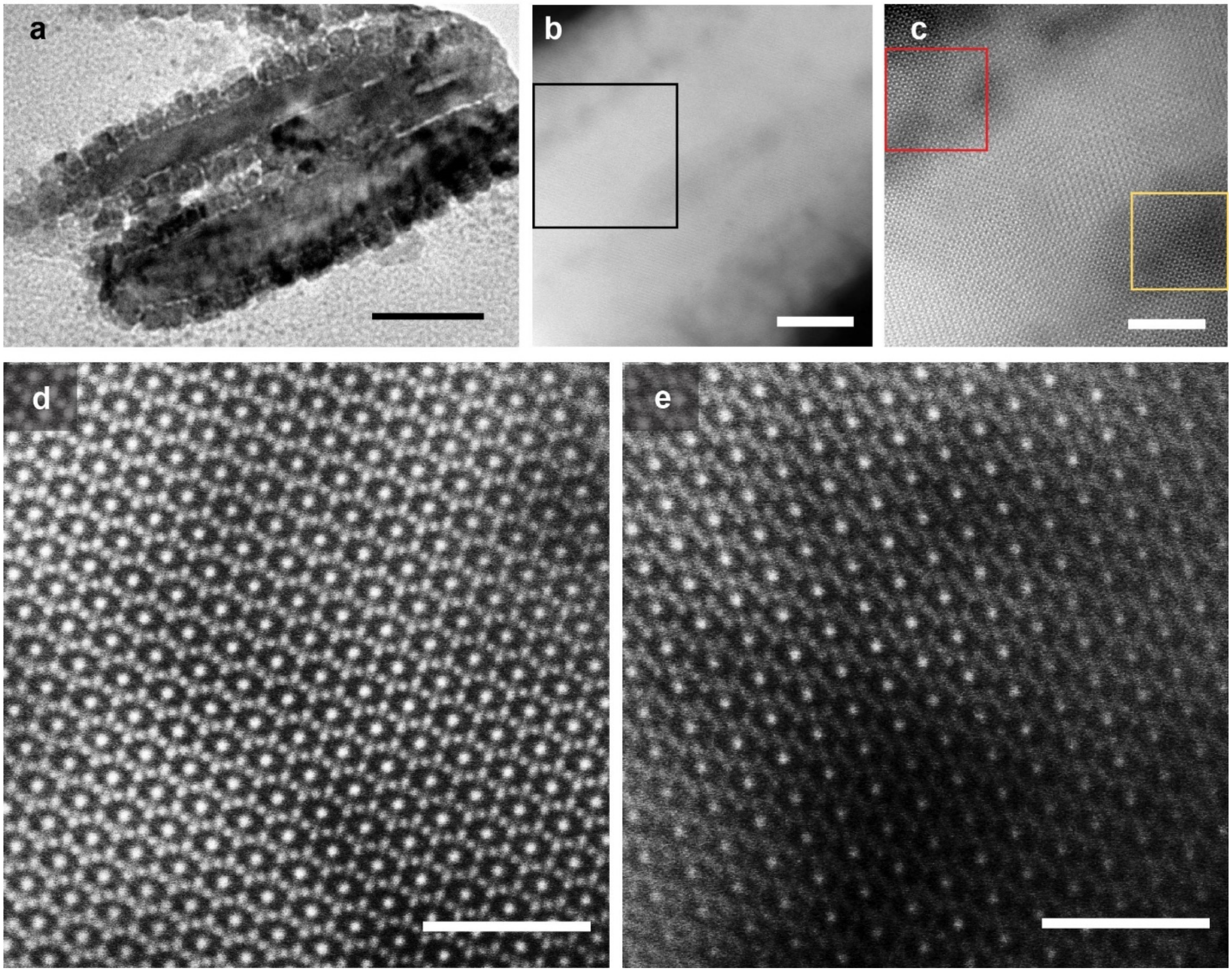

Supplementary Fig. 3| Images of cross-sectioned CFONDs. a, A cross-sectional TEM image of a core-shell Fe_3O_4-$CoFe_2O_4$ nanodisc (CFOND). Scale bar = 50 nm. **b-e,** Cross-sectional scanning TEM (STEM) images of a CFOND at different magnifications demonstrate the epitaxial interface between Fe_3O_4 and $CoFe_2O_4$ layers. **c** is the higher magnification image of the black rectangle in **b**. **d,e,** Higher magnification images of the areas marked with red and yellow rectangles in **c**. Scale bars are 10 nm (**b**), 5 nm (**c**), and 2 nm (**d** and **e**).

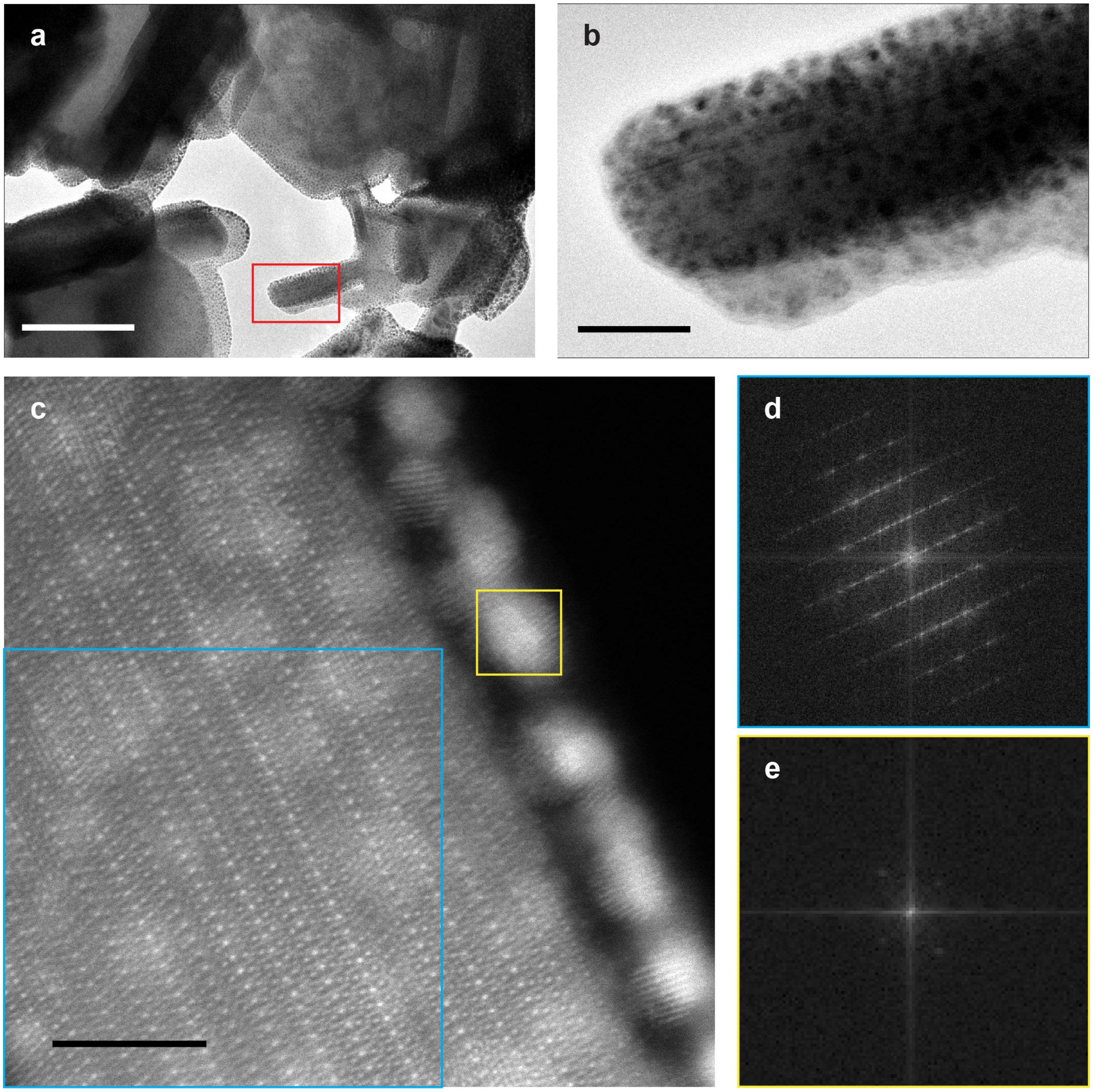

Supplementary Fig. 4| Cross-sectioned images of MENDs. a, Cross-sectional TEM images of a core-double shell Fe_3O_4-$CoFe_2O_4$-$BaTiO_3$ magnetoelectric nanodisc (MEND). **b,** A magnified view of the area marked by a red rectangle in (A). **c,** STEM image demonstrates the crystal structure of $BaTiO_3$, (yellow rectangle) which is distinct from that of Fe_3O_4 or $CoFe_2O_4$ (inverse spinel, cyan rectangle). **d,e,** Fast Fourier transform images of Fe_2O_4-$CoFe_2O_4$ layer (**d**, cyan rectangle in **c**) and $BaTiO_3$ layer (**e**, yellow rectangle in **c**) of the STEM image correspond in **c**. Scale bars are 100 nm (**a**), 20 nm (**b**), 5 nm (**c**).

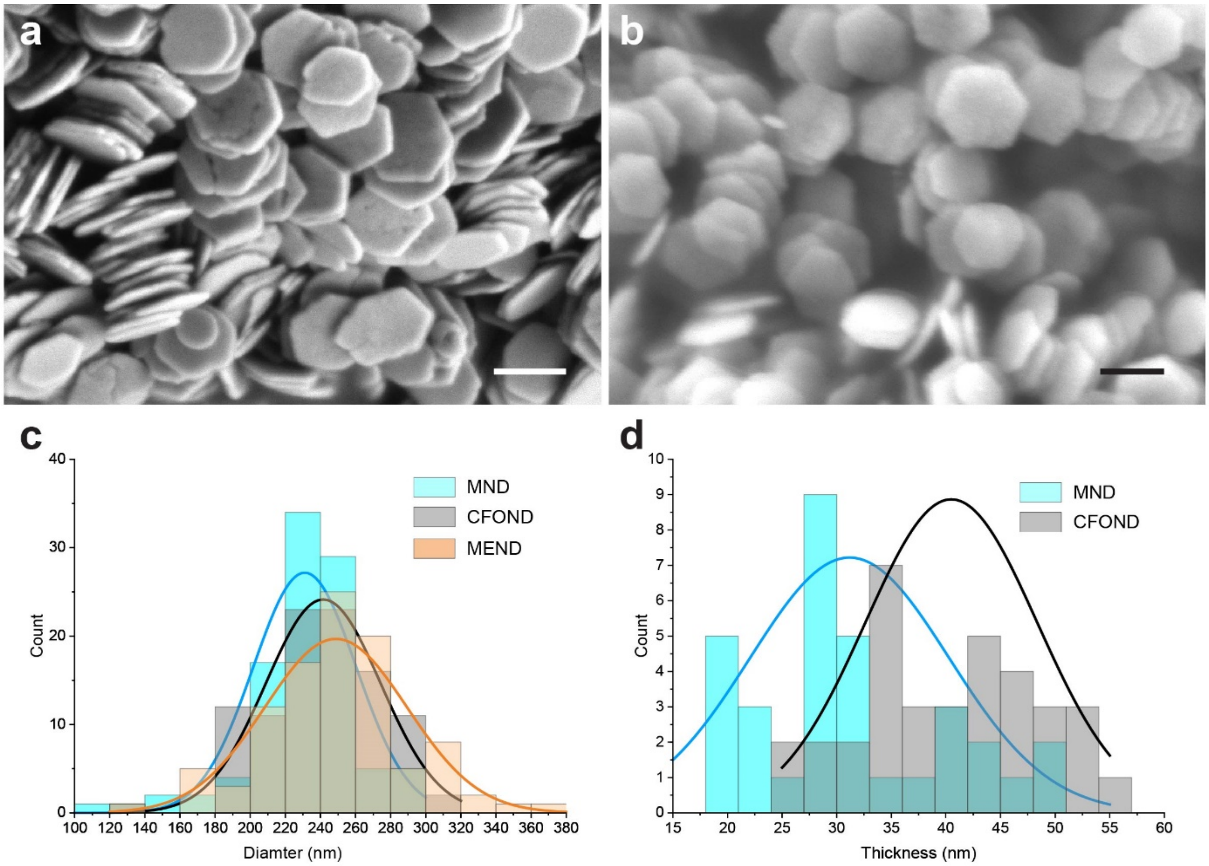

Supplementary Fig. 5 | Average nanoparticle dimensions. a, b, The SEM image of MNDs **(a)** and CFONDs **(b)**. Scale bar = 200 nm. **c,** Histograms of MNDs, CFONDs, and MENDs diameters and the corresponding Gaussian fits with peaks at 231, 240, and 250 nm, respectively. **d,** Histograms of MNDs and CFONDs thicknesses were plotted along with the Gaussian fits with peaks at 31.2 and 40.5 nm, respectively. The thickness of the $BaTiO_3$ layer was estimated from the difference in diameters between CFONDs and MENDs.

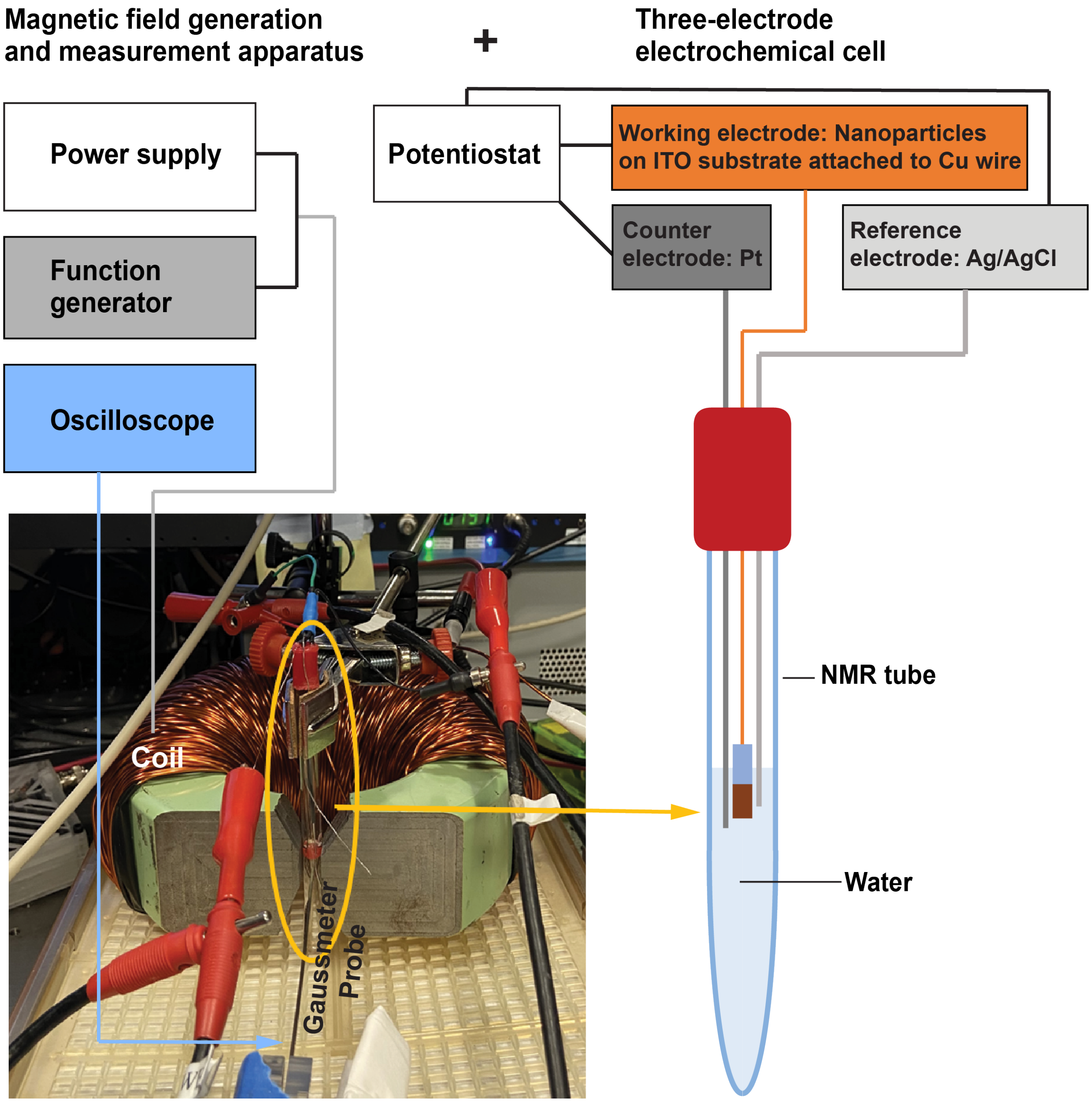

Supplementary Fig. 6| Magnetoelectric coefficient (α_{ME}) measurement apparatus. To generate the magnetic field, TEMCo 14 AWG copper magnet wire was wound around a horseshoe-shaped magnetic core with a 0.5-inch gap. The coil was connected to a power supply (CROWN DC-300A Series II) and signal generator (PICOSCOPE 2204A) to generate a compound magnetic field combining an offset magnetic field (OMF, 0 – 320 mT) with an alternating magnetic field (AMF, 0 – 1 kHz, 0 – 14 mT). A nuclear magnetic resonance (NMR) glass tube (8 mm diameter) was used as the electrochemical cell, which was placed into the 8 mm gap of the horseshoe coil. In the cell, the Ag/AgCl reference electrode, Pt counter electrode, and the working electrode were immersed in the Tyrode solution. The working electrode was comprised of a MEND layer on a conductive indium tin oxide substrate.

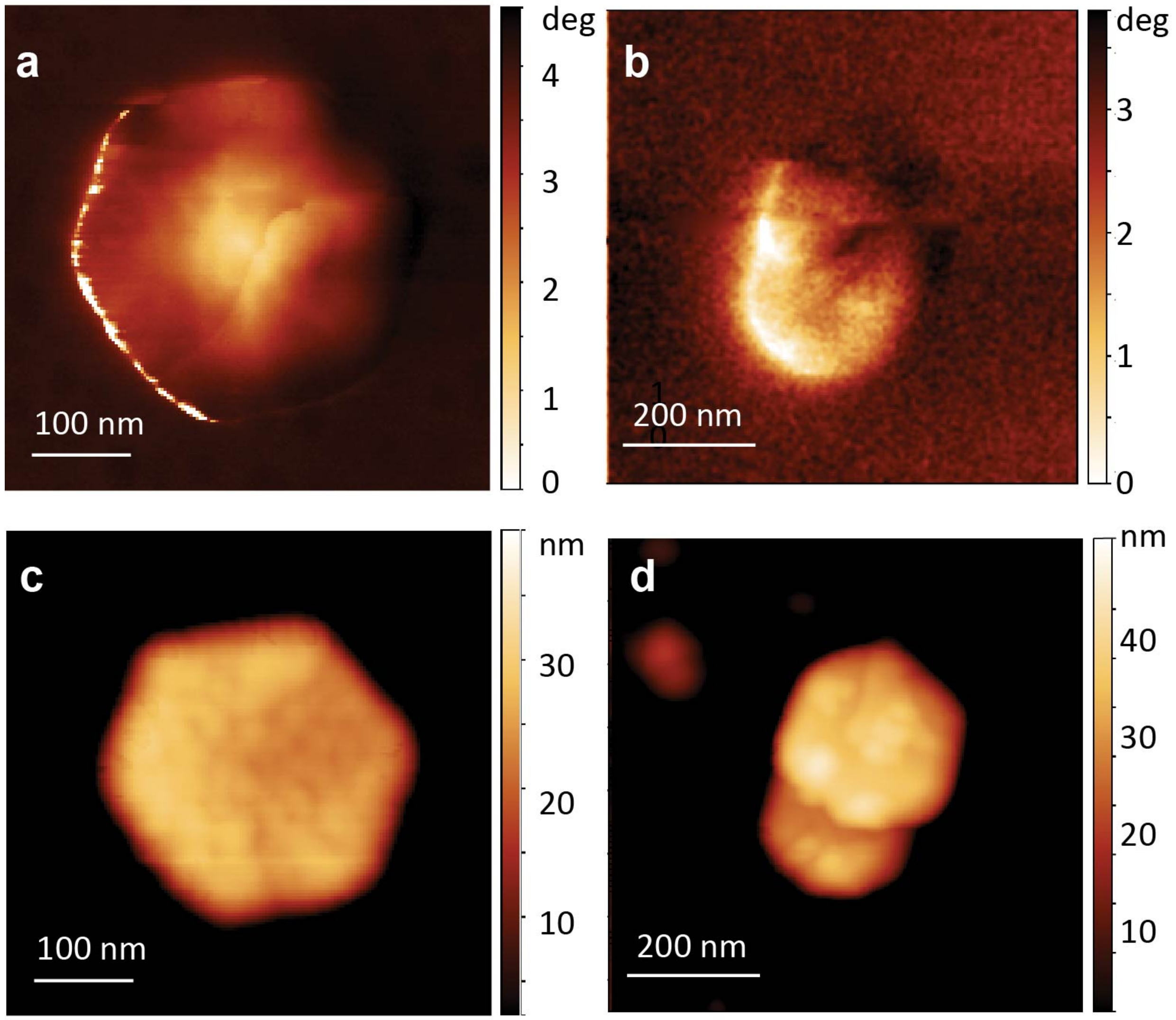

Supplementary Fig. 7| Remnant magnetization configuration of CFONDs. a, b, Magnetic force microscopy (MFM) images of isolated **(a)** and overlapping **(b)** CFONDs. Isolated CFONDs assume the magnetic vortex ground state magnetization, while overlapping CFONDs lose the vortex state in favor of in-plane magnetization. **c, d,** Atomic force microscopy (AFM) images of isolated **(c)** and overlapping CFONDs **(d)** corresponding to the MFM images in (a) and (b), respectively.

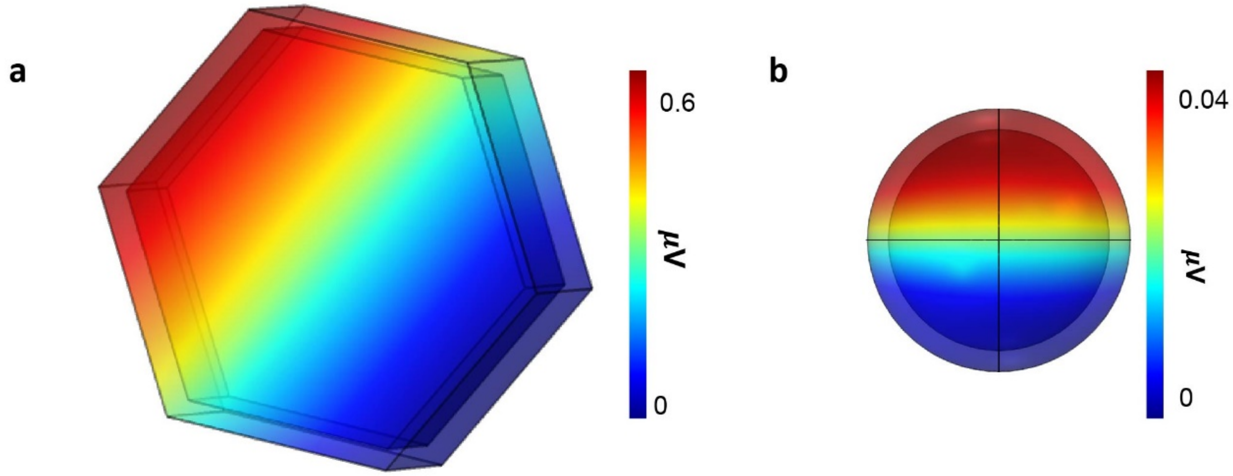

Supplementary Fig. 8| Finite element simulation (COMSOL Multiphysics) of the electric polarization in the piezoelectric $BaTiO_3$ shell based on the implementation of the RMS deformation shown in Figure 1f to the saturation magnetization parameter of the core. a, Hexagonal nanodisc consisting of a 100% Fe_3O_4 magnetostrictive core and a 5 nm piezoelectric $BaTiO_3$ shell. **b,** Isotropic nanoparticle consisting of a magnetostrictive core with a volumetric ratio 92% Fe_3O_4 and 8% $CoFe_2O_4$ and a 5 nm piezoelectric $BaTiO_3$ shell. The elasticity matrix, coupling matrix, and relative permittivity of $BaTiO_3$ shell has been adapted from prior work.[1]

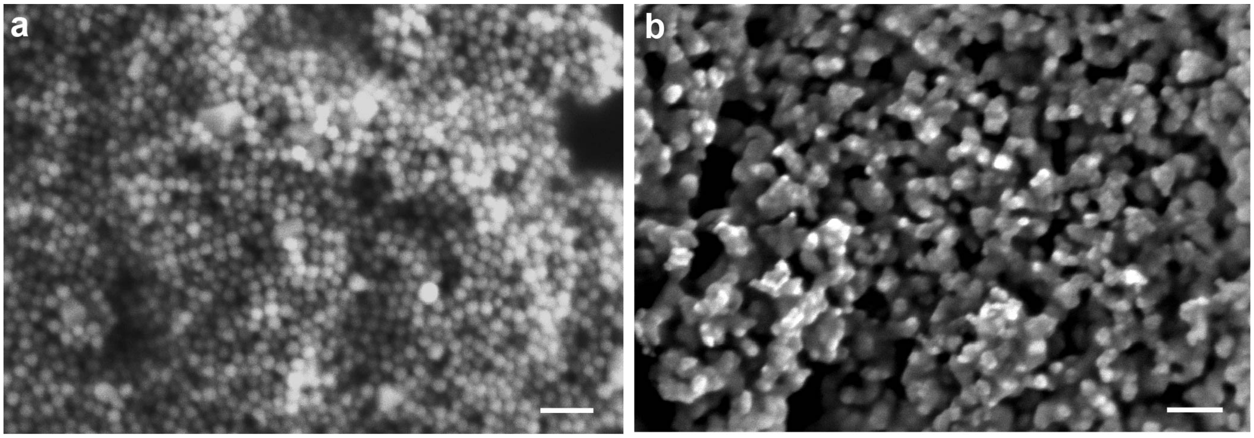

Supplementary Fig. 9| Images of isotropic nanoparticles. Scanning electron microscopy (SEM) images of isotropic $CoFe_2O_4$ nanoparticles with an average diameter of 25 nm **a,** and core-shell $CoFe_2O_4$-$BaTiO_3$ nanoparticles **b,** produced from cores in **a**. Scale bars = 100 nm.

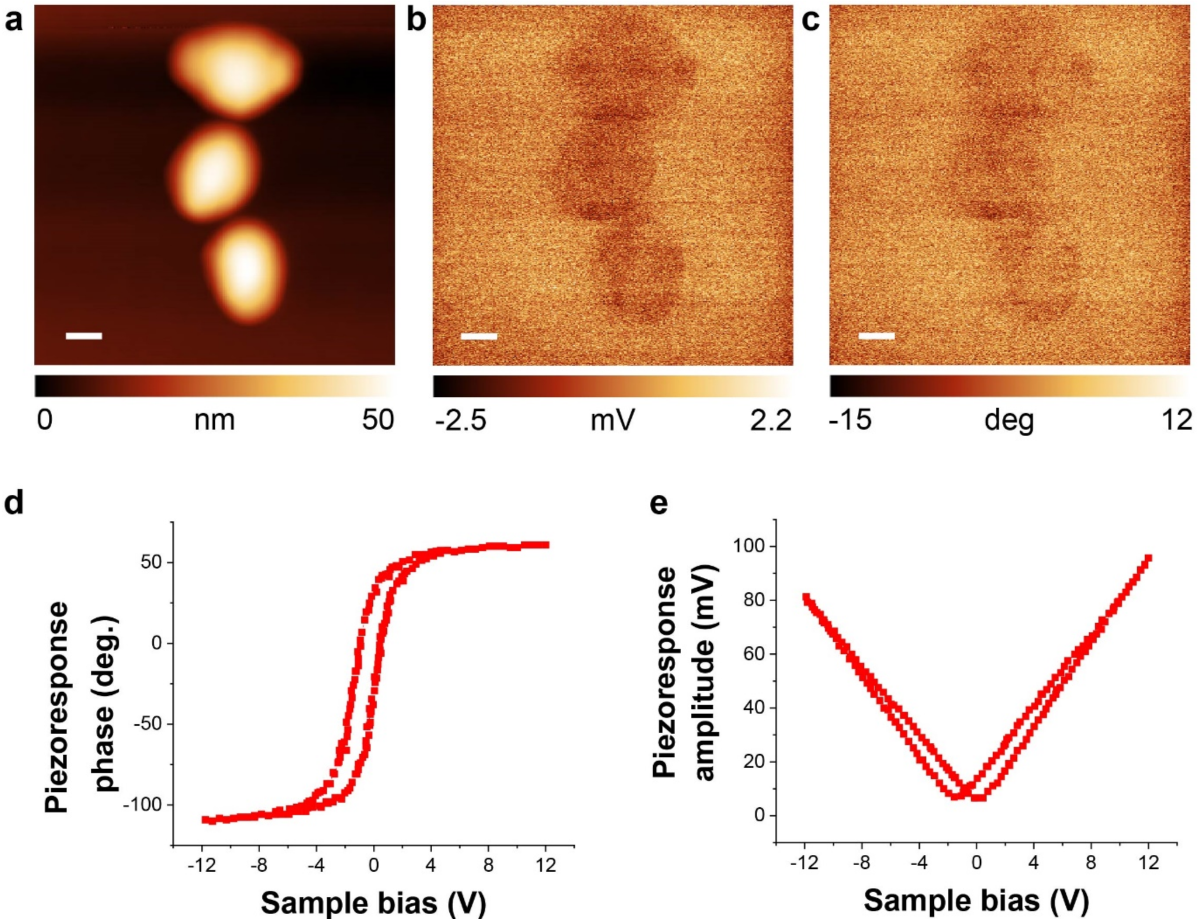

Supplementary Fig. 10 | Piezoresponse of MENDs. The images of **a,** topography, **b,** piezoresponse amplitude, and **c,** phase of MENDs observed via piezoresponse force microscopy. **a-c,** Scale bar= 100 nm **d,** Piezoresponse phase and **e,** amplitude curves.

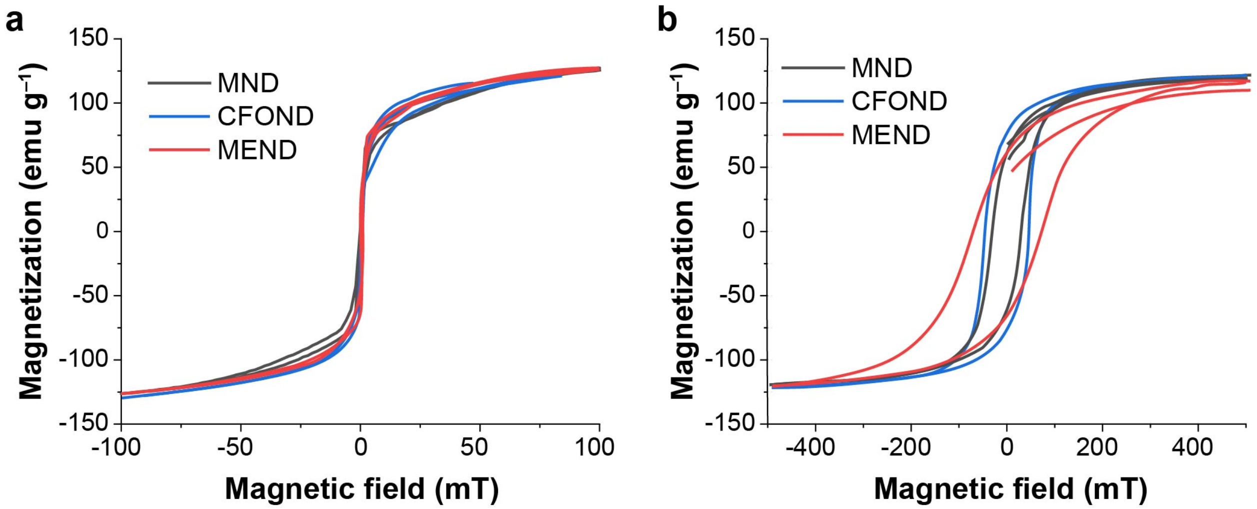

Supplementary Fig. 11| Vibrating sample magnetometry (VSM). VSM of MNDs, CFONDs, and MENDs dispersed in water **(a)** and in a dried pellet form **(b)**.

Table S4. Coercivity (H_C) and saturation magnetization (M_S) of MND, CFOND, and MEND

	**H_C (mT)**	**M_S (emu/g)**
**MND**	38.97±0.76	123.34±8.68
**CFOND**	37.27±4.70	113.42±8.97
**MEND**	66.00±7.37	116.64±4.78

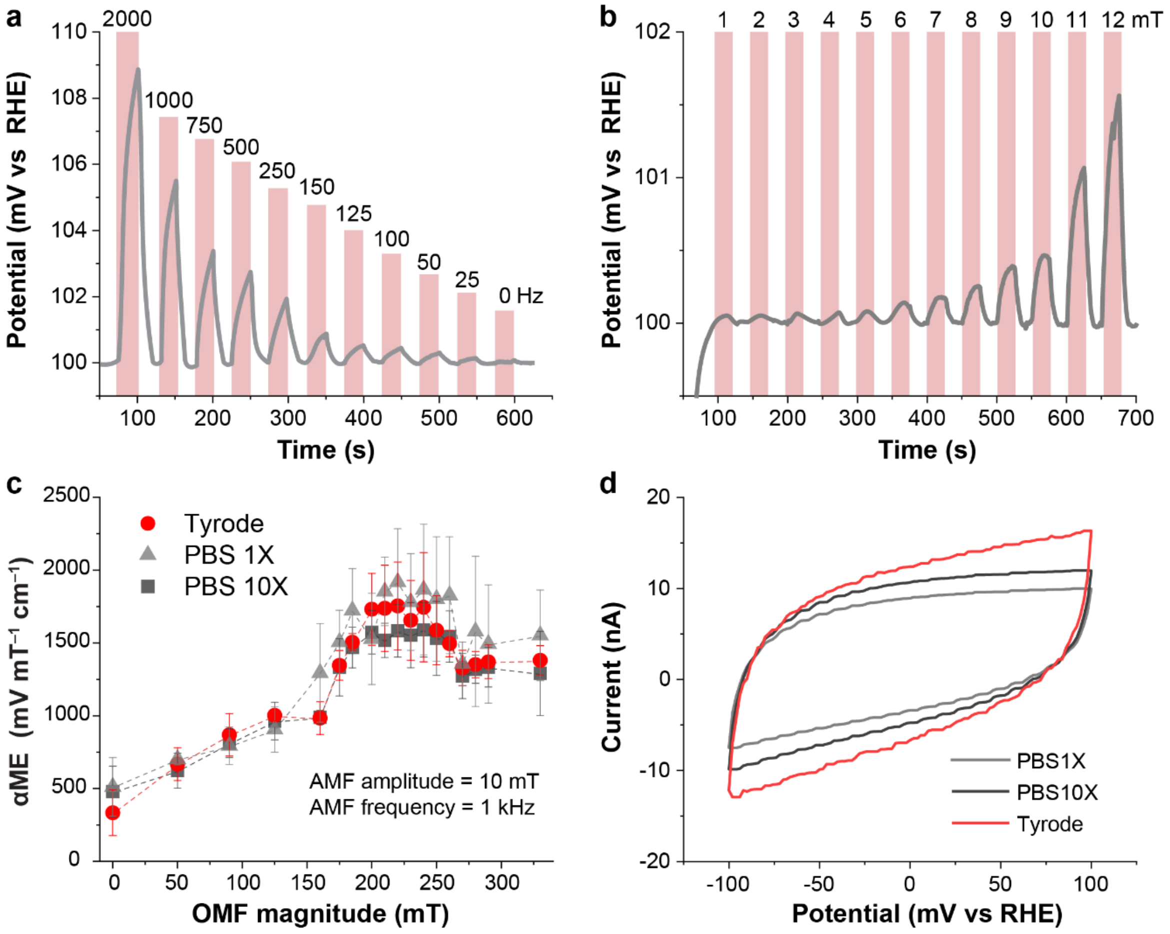

Supplementary Fig. 12| Measurement of magnetoelectric coefficients. a, Changes in potential corresponding to pulses of AC magnetic field with an amplitude of 10 mT and frequencies 25, 50, 75, 100, 125, 150, 250, 500, 1000, and 2000 Hz. **b,** Changes in potential corresponding to pulses of AC magnetic field with a frequency of 150 Hz and amplitudes 1, 2, 3, 4, 5, 6, 7, 8, 9, 10, 11, and 12 mT. Multiple repetitions (n=3) of these measurements were conducted to generate data displayed in **Fig. 1i, j**. The pink boxes indicate the time points when the magnetic field was applied. **c,** α_{ME} at an AMF with a frequency f_{AMF} =1 kHz and amplitude H_{AMF} = 10 mT measured at varying magnitudes of OMF for MENDs in Tyrode (red), PBS 1X (light grey), and PBS 10X (dark grey). The error bar indicates the standard deviation. Data are shown as mean +/- SD. **d,** The non-faradaic current in cyclic voltammetry (CV) at 25 mV/s scan rate, which implies the electric double layer capacitance within different electrolytes from the equation $I = dQ/dt = d(C_{dl}E)/dt = C_{dl}\nu$, where I is non-faradaic current, Q is charge, t is time, C_{dl} is electric-double layer capacitance, E is potential, and ν is the scan rate of CV.

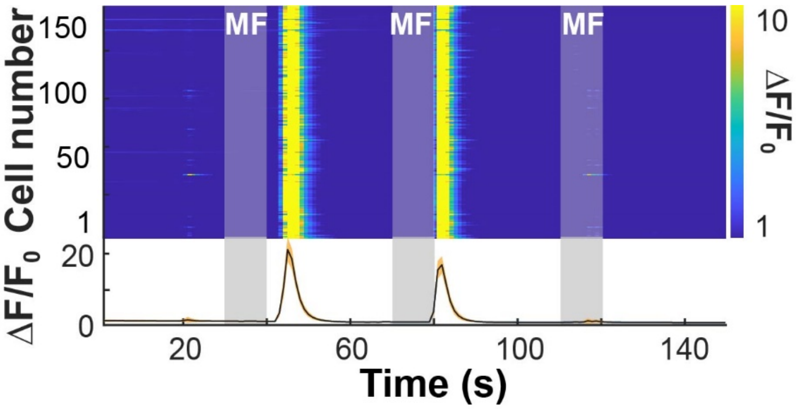

Supplementary Fig. 13| GCaMP6s fluorescence imaging at 1 kHz AMF frequency. Traces of GCaMP6s $\Delta F/F_0$ of individual (top) and average (bottom) hippocampal neurons decorated with MENDs in response to 10 mT AMF with frequencies 1000 Hz (OMF magnitude 220 mT). The grey bars indicate MF epochs.

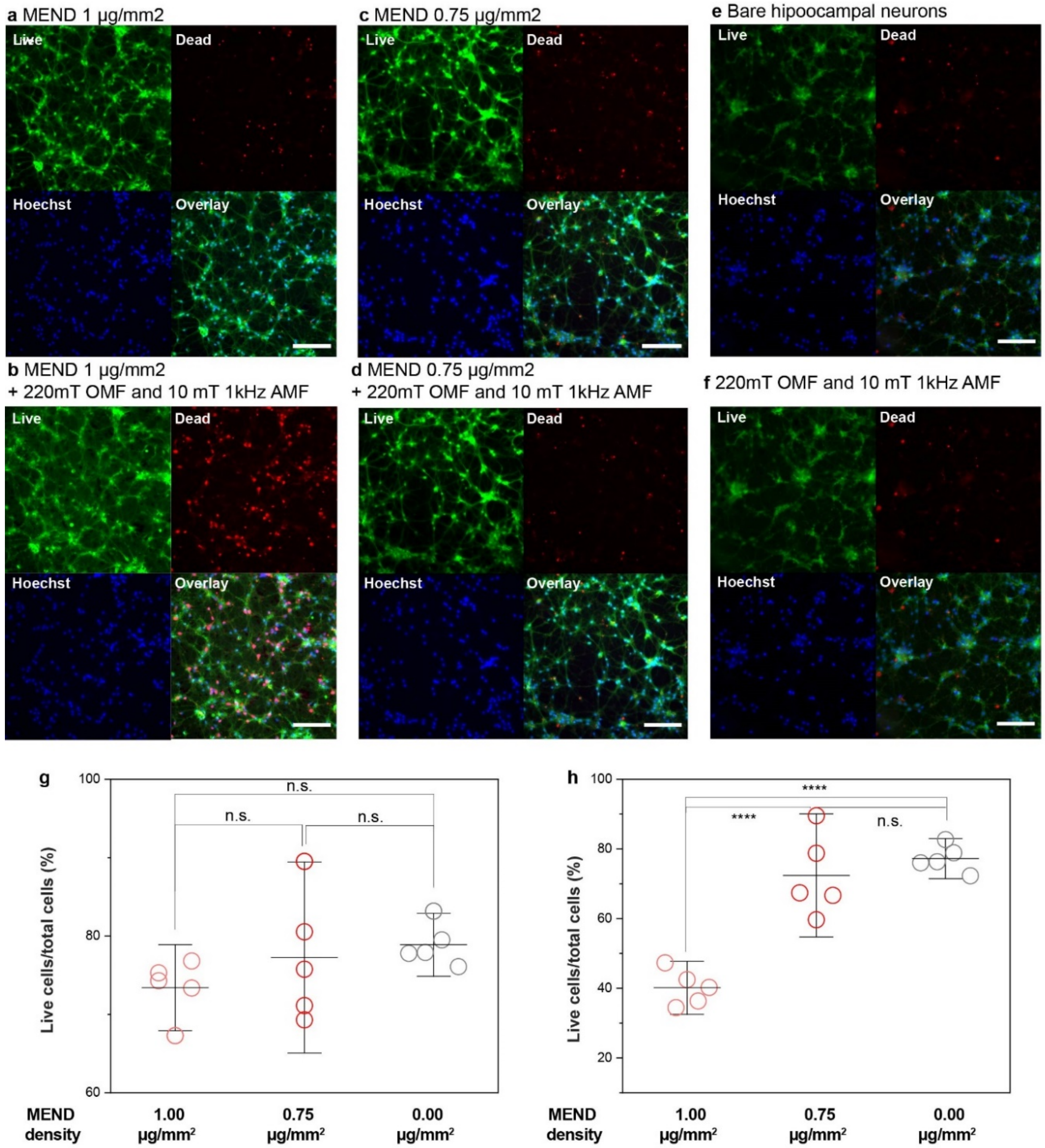

Supplementary Fig. 14| Quantification of cell viability. Live-dead assay of neurons decorated with **a,b**, 1 µg mm^{-2}, **c,d,** 0.75 µg mm^{-2}, and **e,f,** without MENDs (**a,c,e**) before and (**b,d,f**) after three cycles of MFs. Green – linve cell, Red – dead cells, Blue – nuclei, Scale bar = 150 µm. Live cell numbers normalized with the number of total cells **c,** before and **d,** after MF application on neurons decorated with MENDs at densities of 1 µg mm^{-2}, 0.75 µg mm^{-2}, and without MENDs. Statistical significance was tested via Kruskal-Wallis ANOVA and Tukey's multiple comparison tests (n = 5 plates per condition, P=0.515 (before) and 6.98×10^{-5} (after) for 1 vs 0.75 µg mm^{-2} ; P=0.278 (before) and 1.78×10^{-5} (after) for 0.75 vs 0 µg mm^{-2} , P=0.881 (before) and 0.598 (after) for 0.75 vs 0 µg mm^{-2} ; ****P ≤ 0.0001, n.s. P > 0.05). The center lines and error bars indicate the mean and standard deviation (SD), respectively.

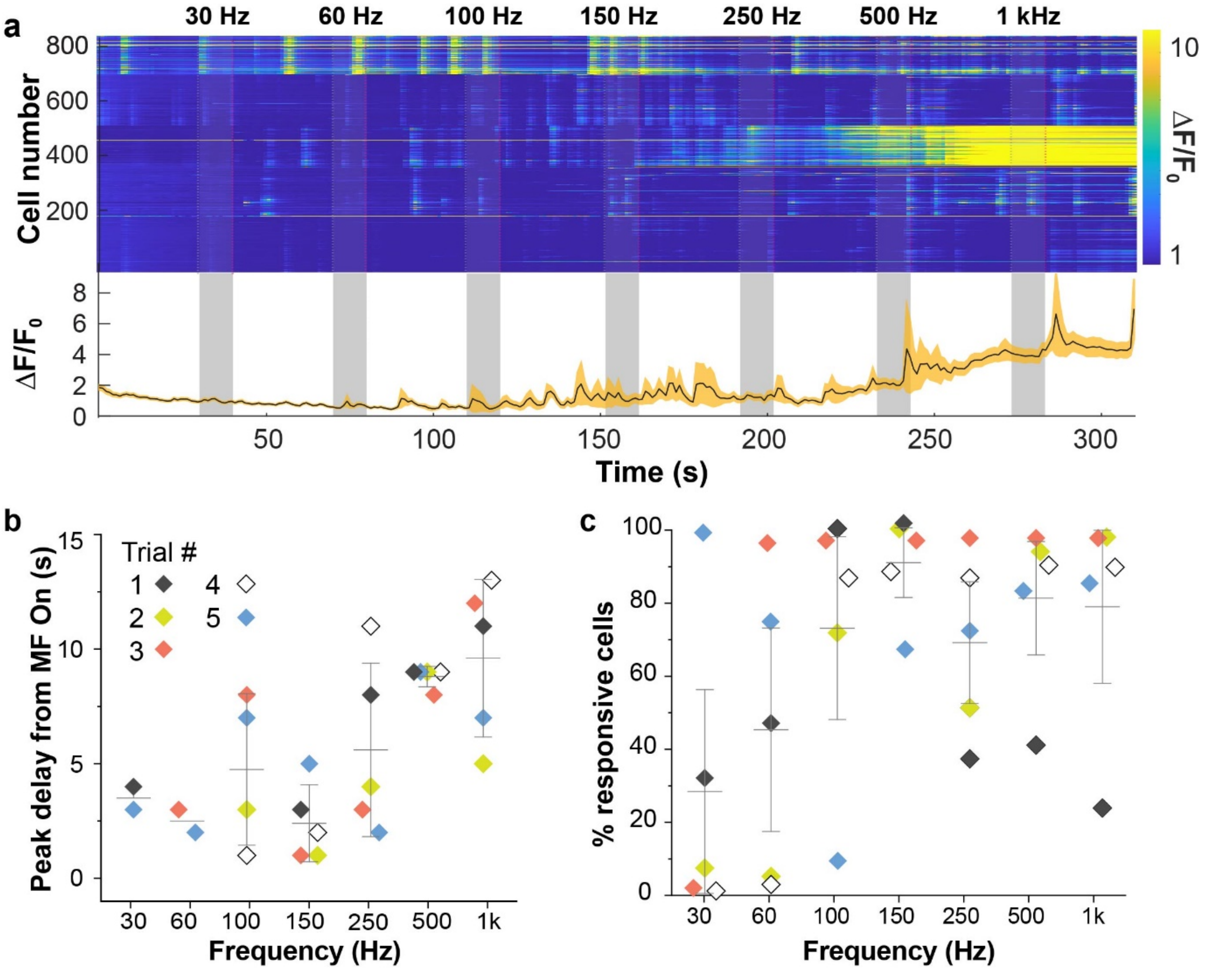

Supplementary Fig. 15| GCaMP6s fluorescence imaging at different AMF frequencies. a, Traces of GCaMP6s $\Delta F/F_0$ of individual (top) and average (bottom) of hippocampal neurons (n=5 culture plates) decorated with MENDs in response to 10 mT AMF with frequencies 30, 60, 100, 150, 250, 500, and 1000 Hz (OMF magnitude 220 mT). The grey bars indicate MF epochs. Bottom panel: The line and shaded area indicate the mean and standard error of the mean (s.e.m.), respectively. **b,** Latency of the peak of GCaMP6s fluorescence transient relative to the start of MF epoch. **c,** The extent of neuronal response to the MF application. The markers and error bars indicate individual data points and (SD), respectively.

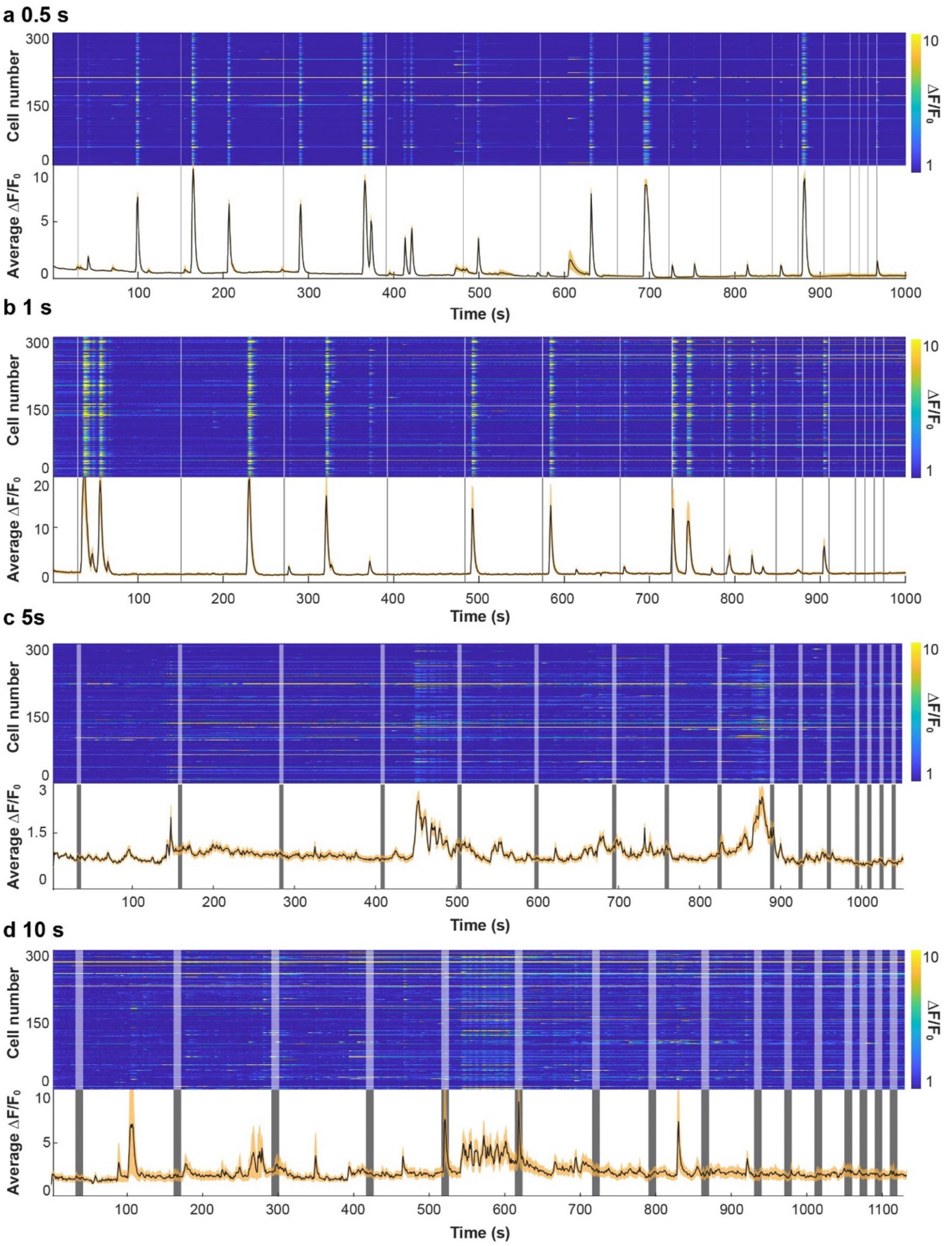

Supplementary Fig. 16| Population (top panels, 300 neurons from 3 plates) and average $\Delta F/F_0$ (bottom panels) of GCaMP6s signals for neurons decorated with MENDs and subjected to magnetic field epochs of different length a, 0.5 s; **b,** 1 s; **c,** 5 s; **d,** 10 s) at intervals of 120 s, 90 s, 60 s, 30 s, 10 s). Grey vertical boxes mark magnetic field epochs (220 mT OMF; 150 Hz, 10 mT AMF). . Bottom panels: The lines and shaded areas indicate the mean and s.e.m., respectively.

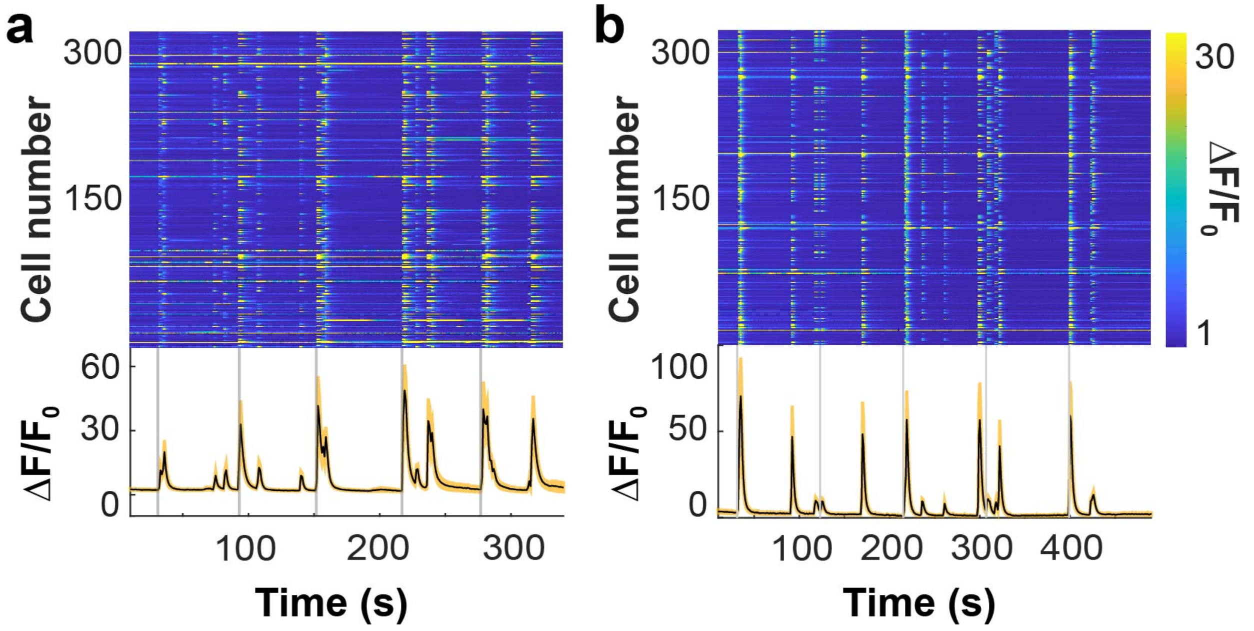

Supplementary Fig. 17| GCaMP6s fluorescence imaging of neurons with 60 s and 90 s intervals between MF epochs. GCaMP6s calcium (Ca^{2+}) imaging of 300 primary hippocampal neurons (n=3 culture plates) with **a,** 60s and **b,** 90s intervals between 2s MF (100 Hz, 10 mT AMF; 220 mT OMF) epochs. Bottom panels: The lines and shaded areas indicate the mean and s.e.m., [illegible]

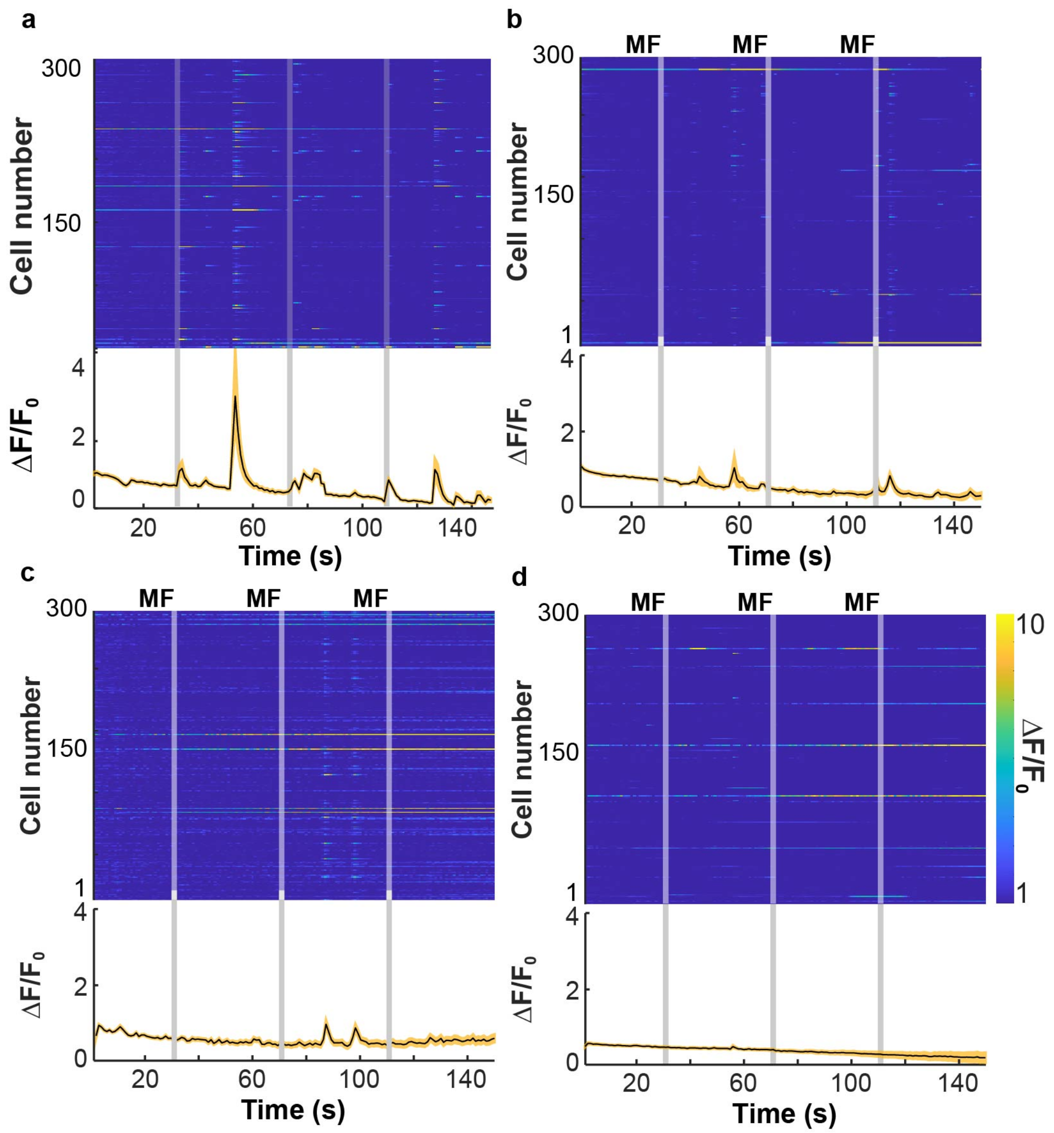

Supplementary Fig. 18| GCaMP6s calcium (Ca^{2+}) imaging of 300 primary hippocampal neurons (n=3 culture plates). With MEND densities **a,** 0.75 μg/mm^2 **b.** 0.5 μg/mm^2 **c,** 0.25 μg/mm^2 **d,** 0 μg/mm^2. Each row in the top panel represents a neuron. The color bar marks fluorescence intensity change ΔF normalized to baseline fluorescence F_0 (averaged between 10-30 s). Vertical grey boxes mark magnetic field epochs (MF, 2 s, 220 mT DC, 150 Hz and 10 mT AC). Bottom panels: The lines and shaded areas indicate the mean and s.e.m., respectively.

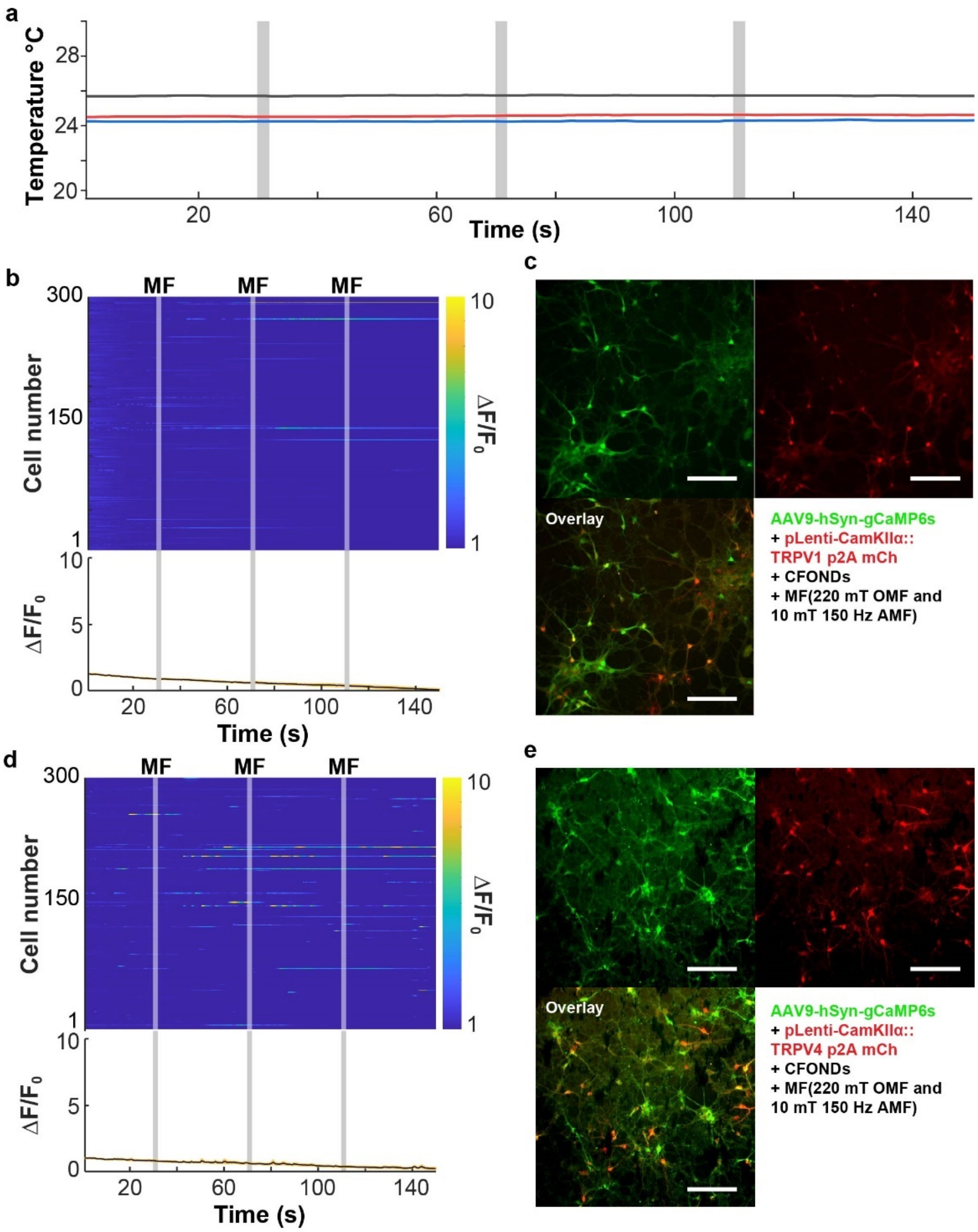

Supplementary Fig. 19| GCaMP6s fluorescence imaging of neurons expressing thermo- and mechano-receptors. The combined MF (220 mT OMF; 10 mT, 150 Hz AMF) did not evoke excitation in neurons decorated with CFONDs that constitute magnetic cores of MENDs (without the piezoelectric $BaTiO_3$ shell) even in the presence of virally delivered thermosensitive or mechanosensitive ion channels. **a,** Temperature traces showing no substantial changes during imaging; three lines (red, blue, black) represent 3 independent recordings. Vertical grey bars indicate combined MF application. **b, d** Fluorescence changes in 300 primary neurons (n=3 culture plates) expressing **(b)** heat sensitive cation channel TRPV1 (transient receptor potential vanilloid family member 1) or **(d)** mechanosensitive cation channel TRPV4. **c, e** Image of neurons expressing **(c)** TRPV1 or **(e)** TRPV4 (top right), GCaMP6s (top left), and their overlays (bottom left). Scale bars are 150 μm.

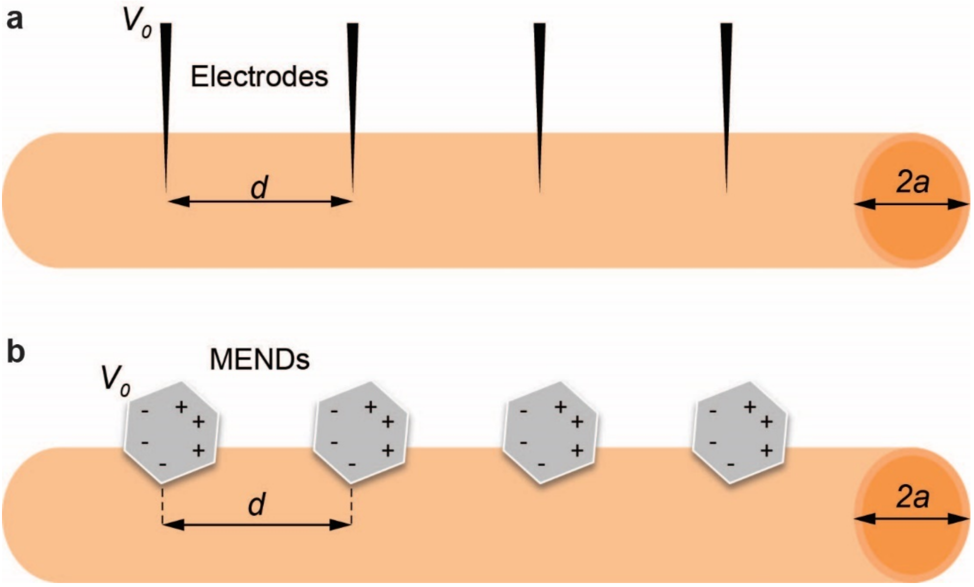

Supplementary Fig. 20| Schematic illustration of the model for the mechanistic study. a, An illustration of the classic cable model in the presence of multiple voltage sources. **b,** An analogous model using MENDs as voltage sources. Here d is the inter-electrode or inter-particle distance, a is the axon radius, and V_0 is the potential applied by an electrode or generated by an individual MEND.

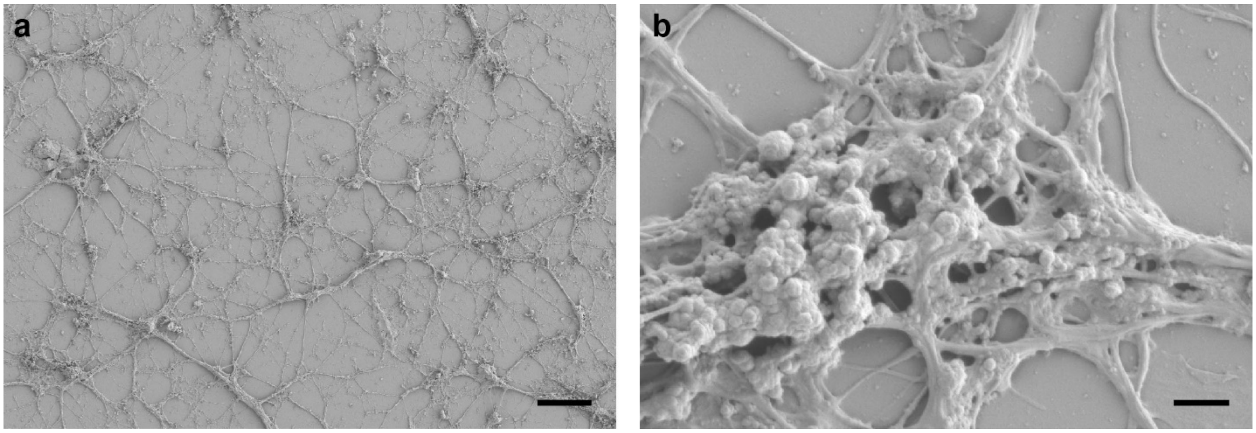

Supplementary Fig. 21| SEM images of neurons. SEM images of primary hippocampal neurons at 10 days in vitro without any nanoparticles at **a,** 200X (Scale bar = 50 μm) and **b,** 4000X magnification. (Scale bars = 2 μm). Since synaptic boutons in intact neurons exhibit dimensions comparable to MENDs, energy-dispersive X-ray spectroscopy mapping (Supplementary Fig. 22) was employed to further distinguish the particles from neuronal structures by their elemental contents.

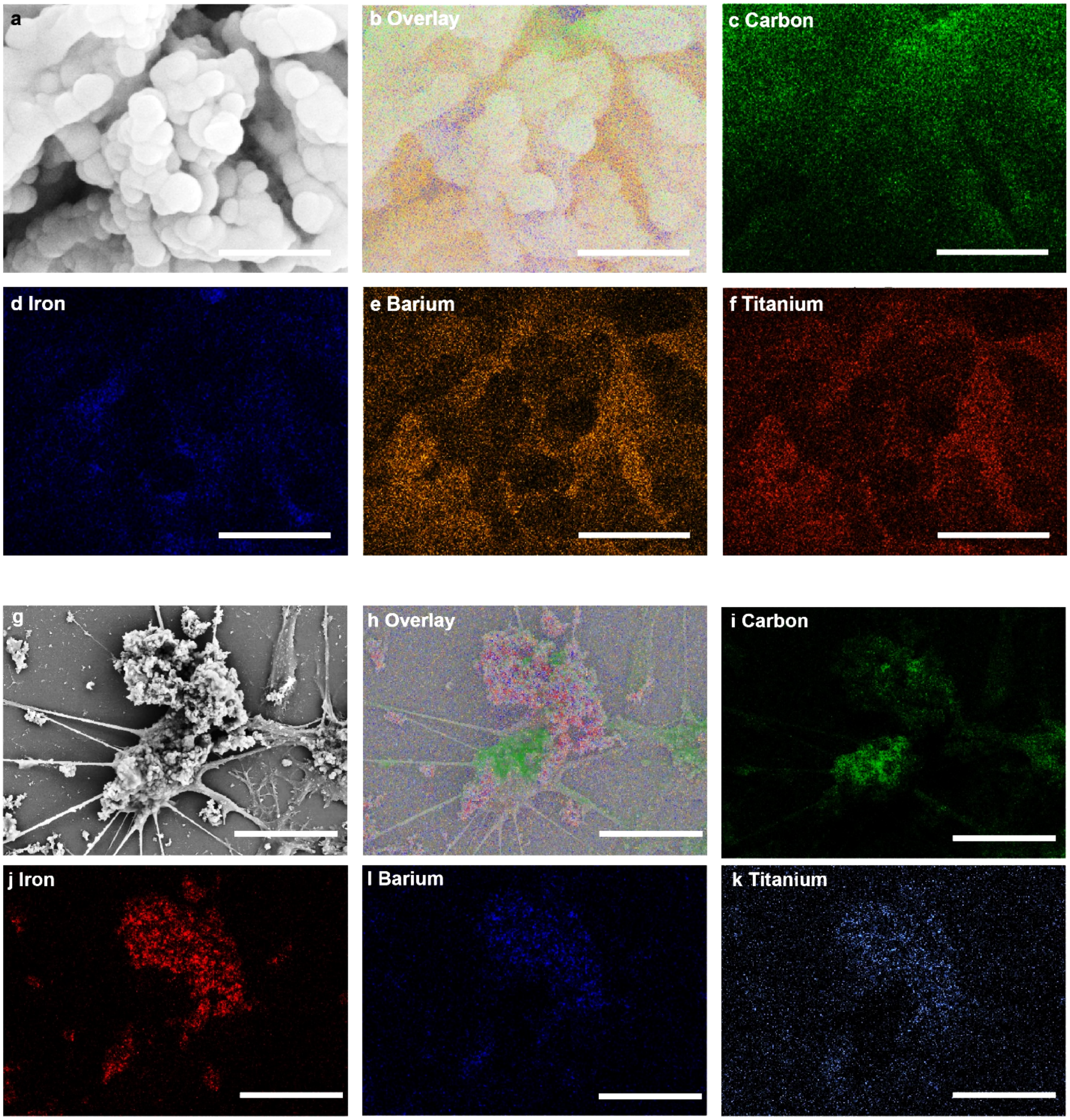

Supplementary Fig. 22| Images of neurons decorated with MENDs. SEM image of hippocampal neurons (10 days) after 1-hour incubation of MENDs in (**a-e**) higher magnification and (**g-l**) lower magnification. **b-f** and **h-l,** Energy-dispersive X-ray spectroscopy mapping on the image in **a**. **b,h,** Merged image of the carbon (C shown in **c,i**), iron (Fe shown in **d,j**), Barium (Ba shown in **e,l**), and titanium (Ti shown in **f,k**) atomic maps. (**a-f**) Scale bars = 500 nm. (**g-l**) Scale bars = 5 μm.

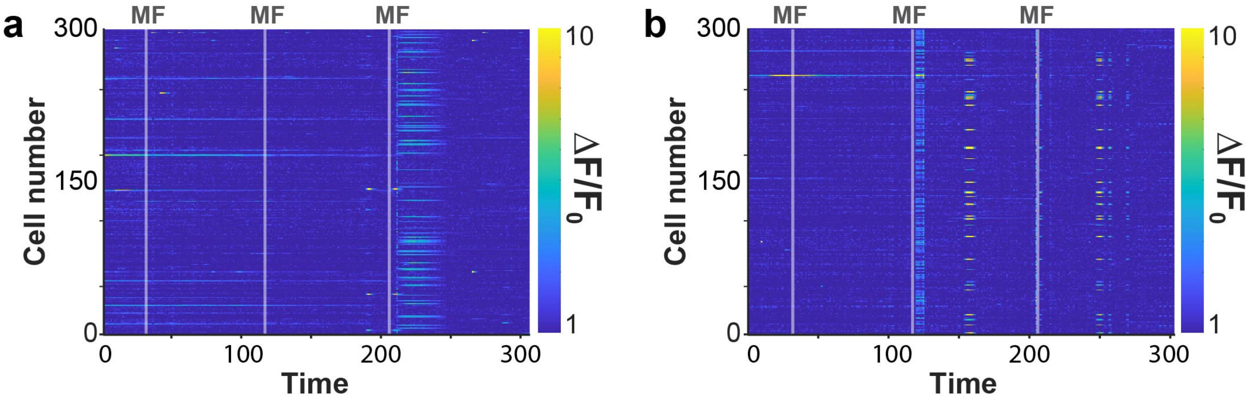

Supplementary Fig. 23| GCaMP6s imaging of neurons in the presence of drugs. a, b GCaMP6s $\Delta F/F_0$ in 300 neurons (n=3 plates) treated with drugs corresponding to the average $\Delta F/F_0$ plots shown in **Fig. 3g,h**. **a,** Neurons treated with tetrodotoxin (TTX, 1 μM). **b,** Neurons treated with 6-cyano-7-nitroquinoxaline-2,3-dione (CNQX, 20 μM) and (2R)-amino-5-phosphonovaleric acid (AP5, 100 μM) cocktail. Vertical grey bars mark MF epochs (2s; 220 mT OMF; 10 mT, 150 Hz AMF).

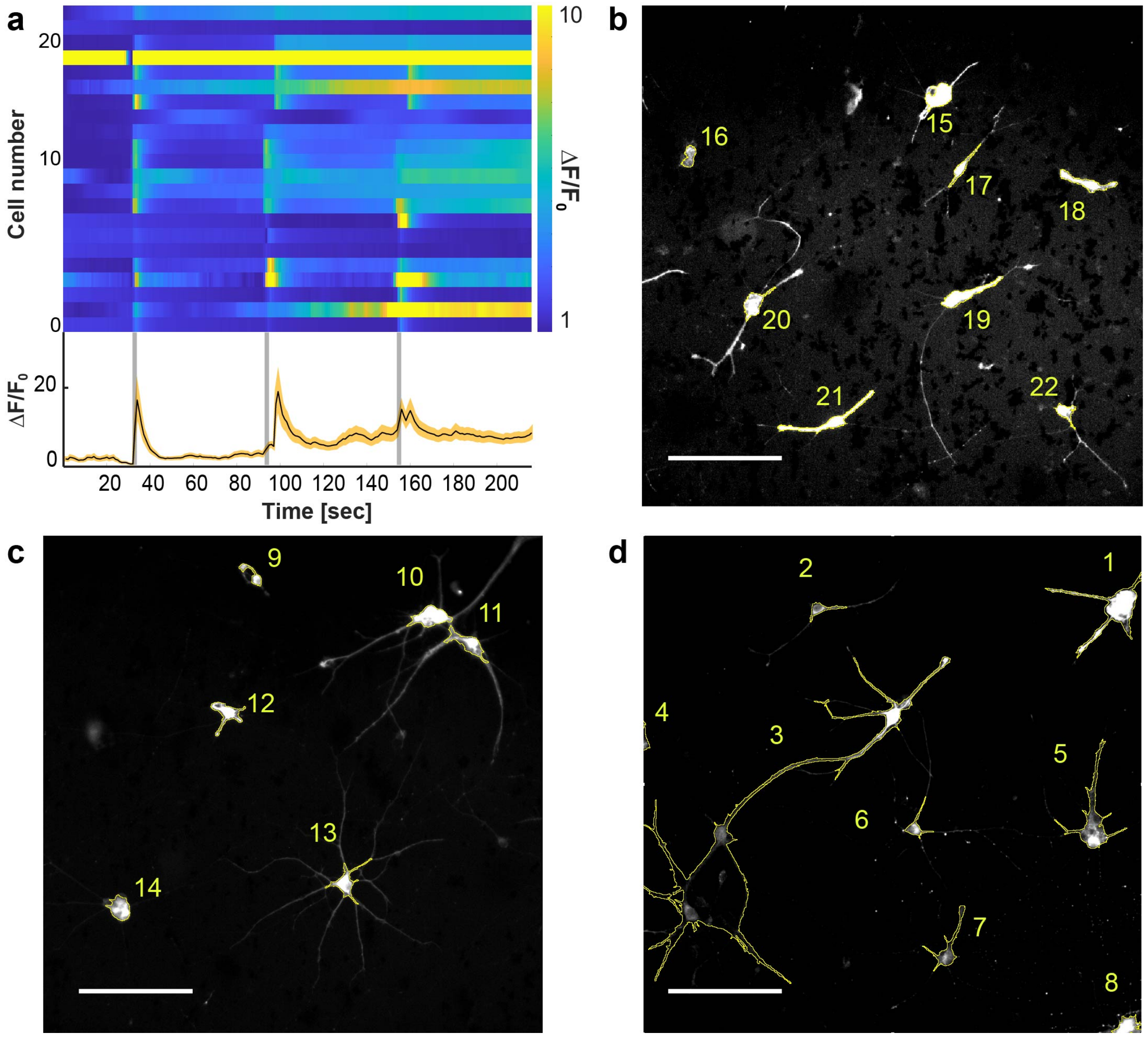

Supplementary Fig. 24| GCaMP6s imaging of neurons cultured at a density of 105 cell/mm^2. a, GCaMP6s $\Delta F/F_0$ from n=3 plates. Vertical grey bars MF field epochs (2s, 220 mT OMF; 10 mT, 100 Hz AMF). **Bottom panel: line** and shaded area indicate mean and s.e.m. **b-d,** Fluorescence images of the 3 plates used for analysis in (a). 22 viable neurons were identified. Scale bars are 150 μm.

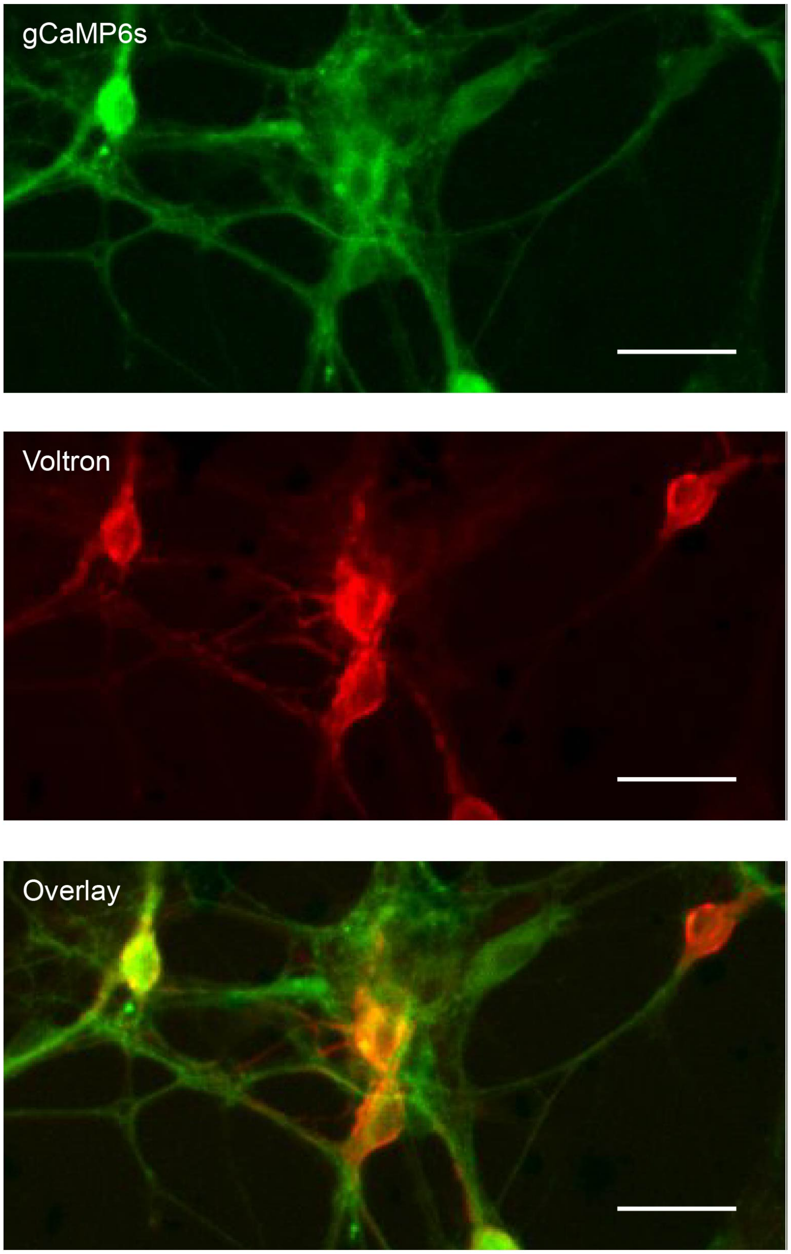

Supplementary Fig. 25| Fluorescent image of cultured neurons co-transfected with Voltron 2.0 and GCaMP6s. GCaMP6s cytoplasmic calcium (Ca^{2+}) indicator and membrane potential indicator Voltron 2.0 were co-expressed in cultured hippocampal neurons. Voltron 2.0 was conjugated to a synthetic dye Janelia Fluor 585 via HaloTag protein labeling system. Scale bars = 40 µm.

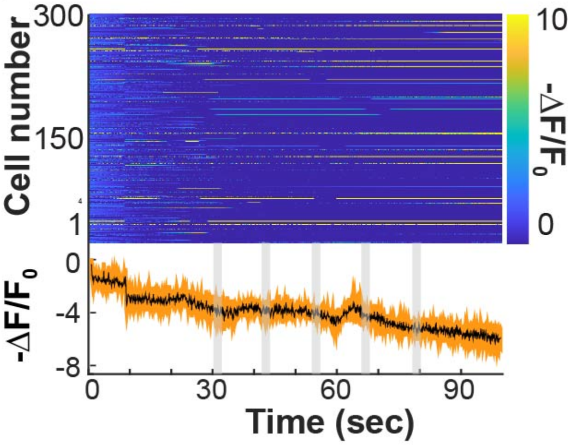

Supplementary Fig. 26| Optical recording of membrane potential fluctuation. The traces of negative relative fluorescence change (–ΔF/F_0) of Voltron 2.0-JF585 recorded in neurons without MENDs. Bottom panel: line and shaded area indicate mean and s.e.m.

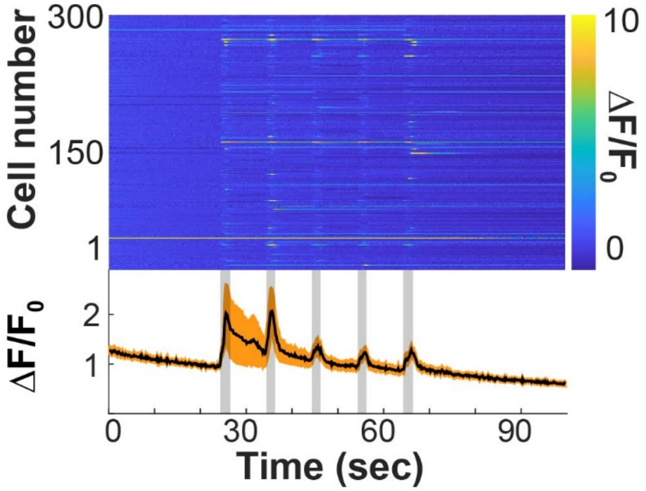

Supplementary Fig. 27| GCaMP6f fluorescence imaging of neurons. Imaging was performed on primary hippocampal neurons (n=3 culture plates) transduced with pAAV.Syn.GCaMP6f.WPRE.SV40 , MF (100 Hz 10 mT AMF and 220 mT OMF) was applied in 2 s epochs with 10 s intervals. Bottom panel: line and shaded area indicate mean and s.e.m.

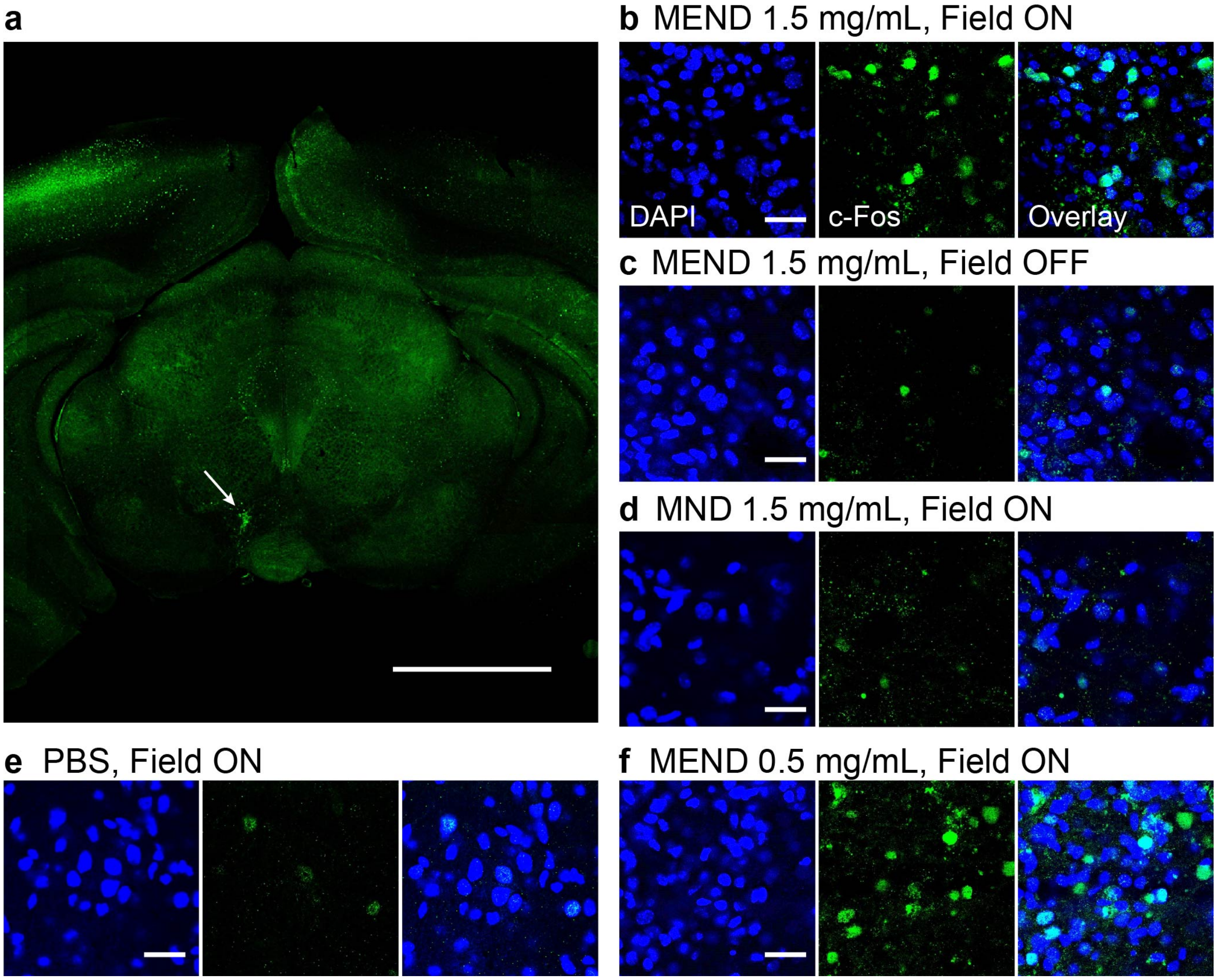

Supplementary Fig. 28| Examples of c-Fos expression in the ventral tegmental area (VTA). **A,** Confocal micrograph of c-Fos expression (green) visible across the entire brain slice, with distinct increase in the VTA on the side injected with MENDs (1.5 µL at 1.5 mg mL^{-1}, marked with an arrow) following exposure to magnetic field (220 mT DC, 150 Hz, 10 mT AC; three 2s epochs separated by 90s rest epochs). Scale bar = 3 mm. **b-f** Higher resolution (20X) confocal images of nuclear stain DAPI (blue), c-Fos (green), and their overlay in brain slices from mice in different experimental groups exposed to various conditions. Scale bars = 25 µm. **b,** A mouse injected with MENDs and exposed to magnetic field. **c,** A mouse injected with MENDs and not exposed to magnetic field. **d,** A mouse injected with control Fe_3O_4 nanodiscs (MNDs, 1.5 µL at 1.5 mg mL^{-1}) and exposed to magnetic field. **e,** A mouse injected with phosphate buffered saline (PBS, 1.5 µL) and exposed to magnetic field. **f,** A mouse injected with a low concentration of MENDs (1.5 µL at 0.5 mg mL^{-1}) and exposed to magnetic field.

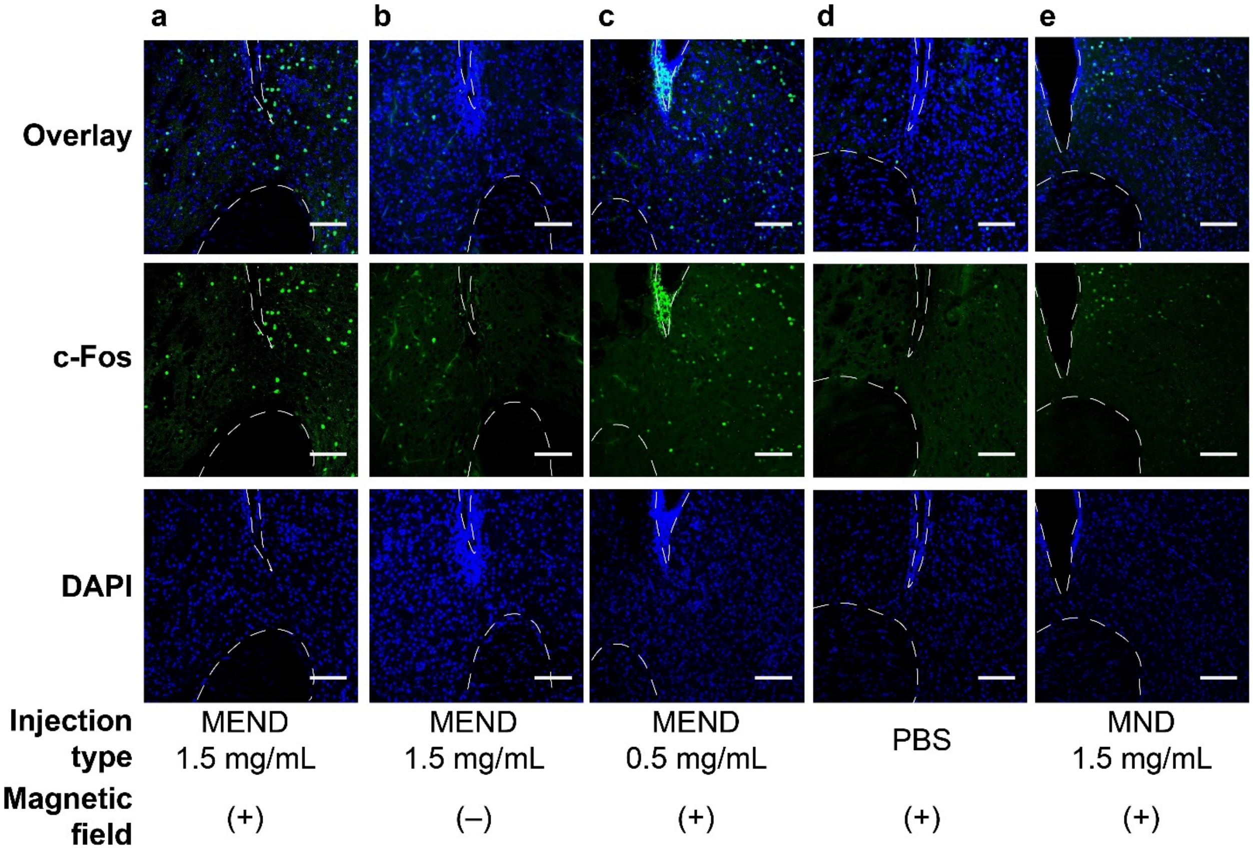

Supplementary Fig. 29| Confocal images of c-Fos expression in the nucleus accumbens (NAc) at different nanomaterial injection and magnetic field application conditions. a, A mouse injected with MENDs (1.0 mg mL^{-1}, 1.5 μL) and exposed to magnetic field (220 mT OMF; 150 Hz, 10 mT AMF, three 2 s epochs separated by 90 s rest epochs). **b,** A mouse injected with MENDs and not exposed to magnetic field. **c,** A mouse injected with a low concentration of MENDs (0.5 mg mL^{-1}, 1.5 μL) and exposed to magnetic field. **d,** A mouse injected with PBS (PBS, 1.5 μL) and exposed to magnetic field. **e,** A mouse injected with control MNDs (1.0 mg mL^{-1}, 1.5 μL) and exposed to magnetic field. c-Fos is marked with green, DAPI is marked with blue. Scale bars = 100 μm.

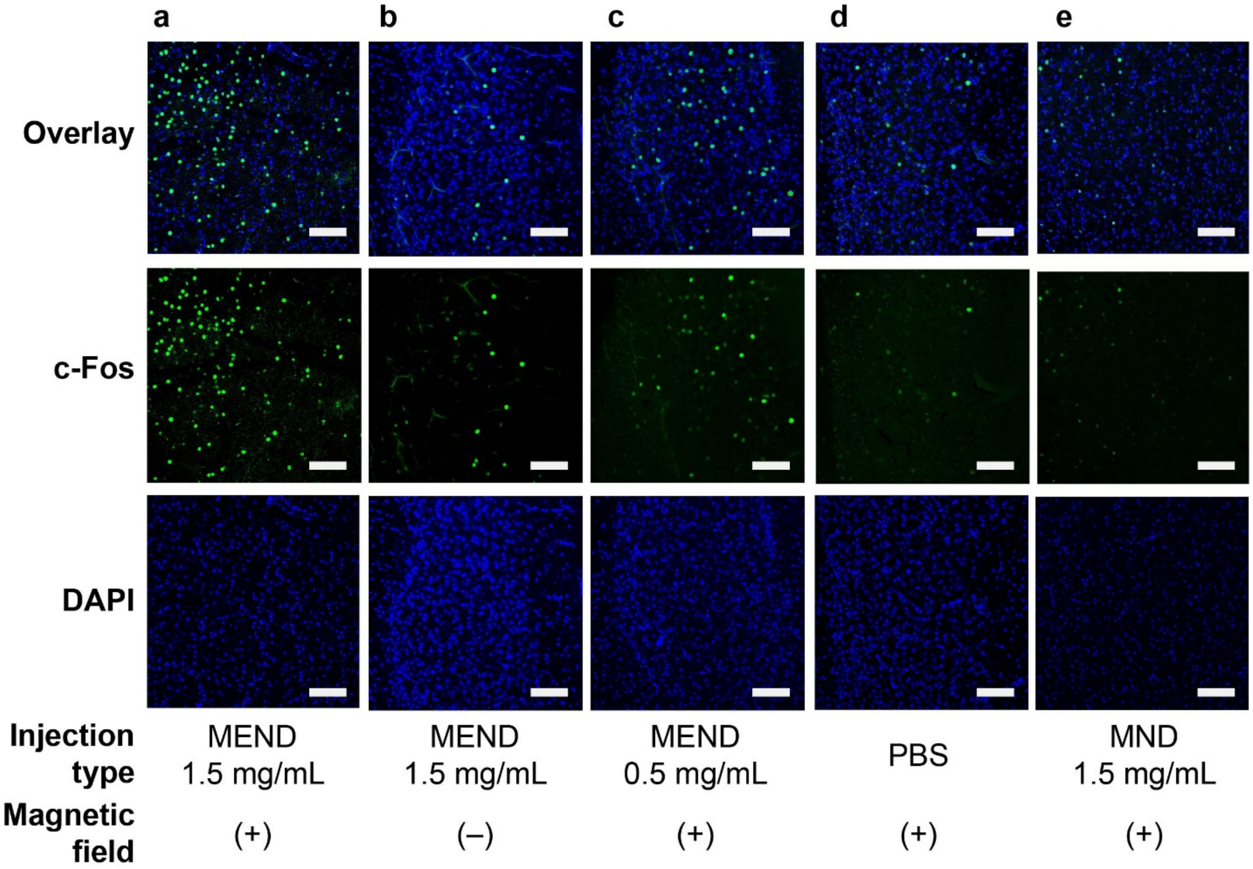

Supplementary Fig. 30| Confocal images of c-Fos expression in the medial prefrontal cortex (mPFC) at different nanomaterial injection and magnetic field application conditions. a, A mouse injected with MENDs (1.0 mg mL^{-1}, 1.5 µL) and exposed to magnetic field (220 mT OMF; 150 Hz, 10 mT AMF, three 2 s epochs separated by 90 s rest epochs). **b,** A mouse injected with MENDs and not exposed to magnetic field. **c,** A mouse injected with a low concentration of MENDs (0.5 mg/mL, 1.5 µL) and exposed to magnetic field. **d,** A mouse injected with PBS (PBS, 1.5 µL) and exposed to magnetic field. **e,** A mouse injected with control MNDs (1.0 mg mL^{-1}, 1.5 µL) and exposed to magnetic field. c-Fos is marked with green, DAPI is marked with blue. Scale bars = 100 µm.

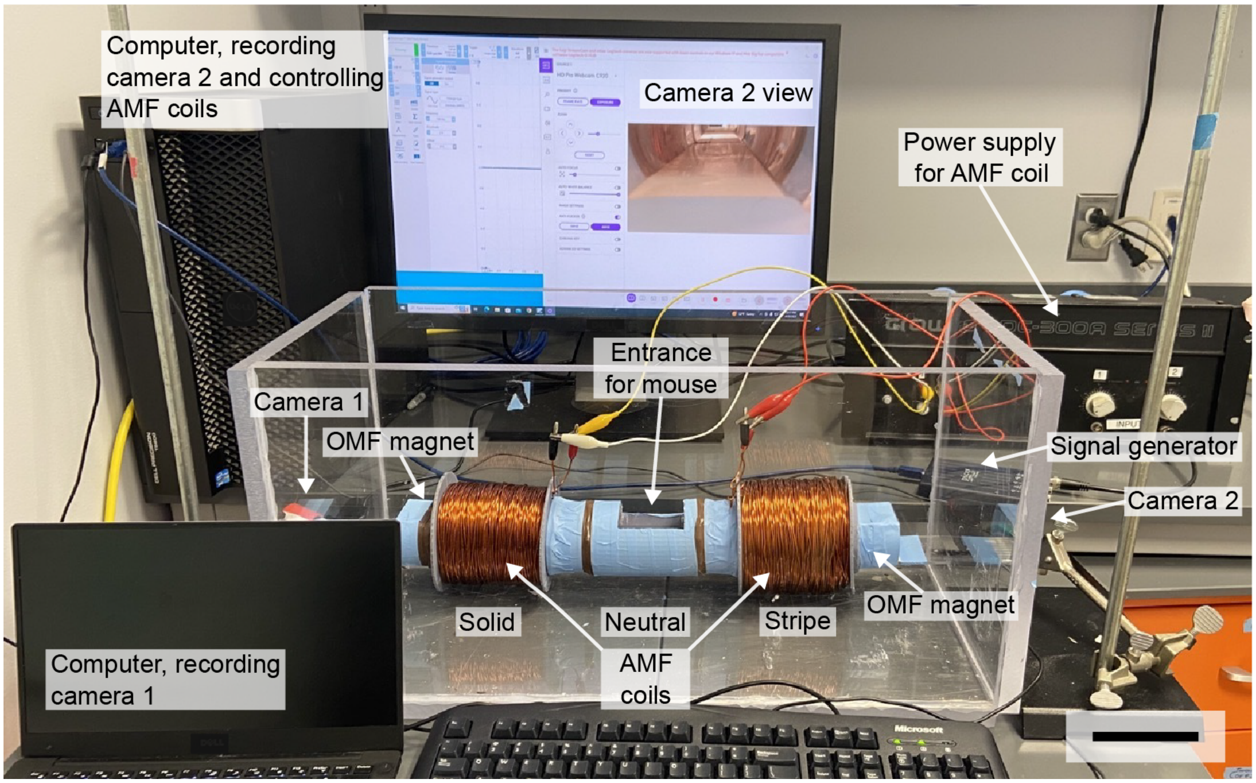

Supplementary Fig. 31| Setup for the place preference behavior assays. A photograph of the custom apparatus employed for place-preference assays enabling application of magnetic fields necessary for magnetoelectric neuromodulation. Scale bar is 10 cm.

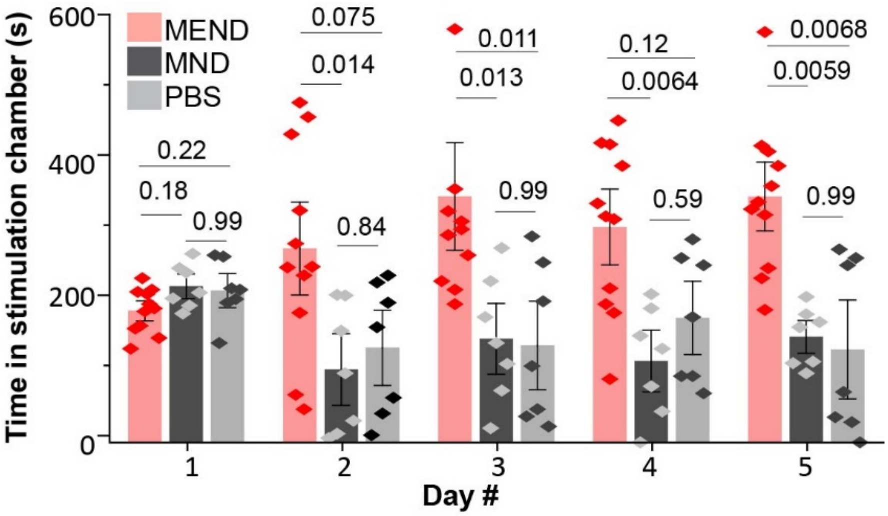

Supplementary Fig. 32| Summary of place-preference assays. Time spent by each subject in the stimulation chamber of the arena, out of a total test time of 600 s for each trial. Scale bars denote standard deviation. (n=11 for MEND group, n=7 for MND and PBS, Kruskal-Wallis test and Tukey's post-hoc comparison test. P-values are noted on the plot. The concentration of MENDs and MNDs was 1.5 mg mL^{-1}, all injections were 1.5 μL.

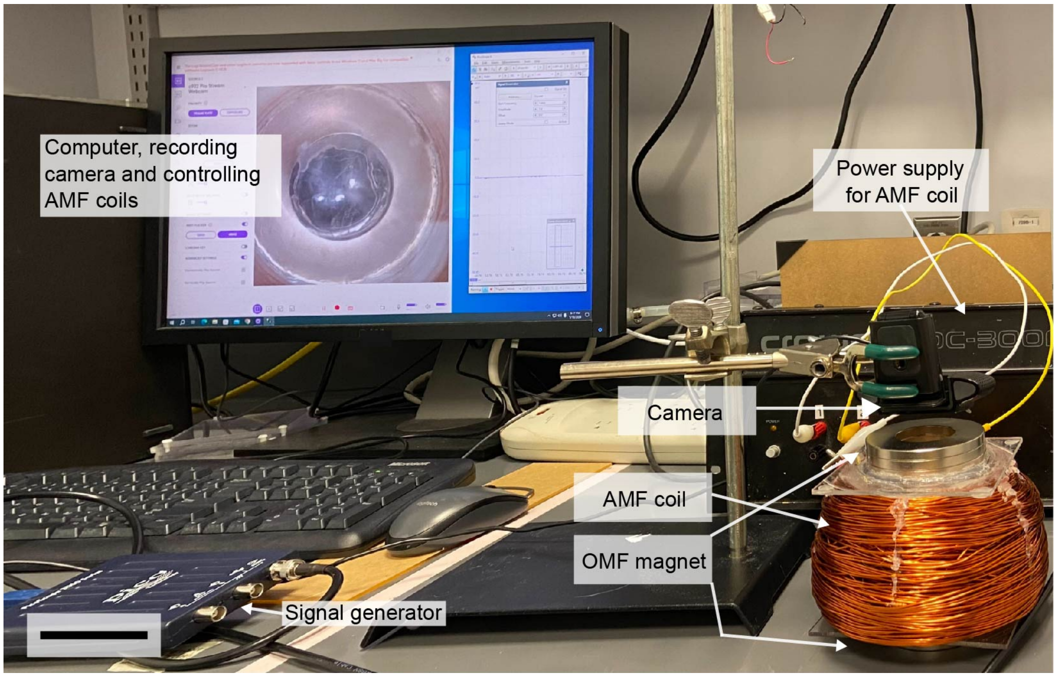

Supplementary Fig. 33| Setup for the rotation behavior assays. A photograph of the custom apparatus employed for rotation behavior inside cylinder enabling application of magnetic fields necessary for magnetoelectric neuromodulation. Scale bar is 7 cm.

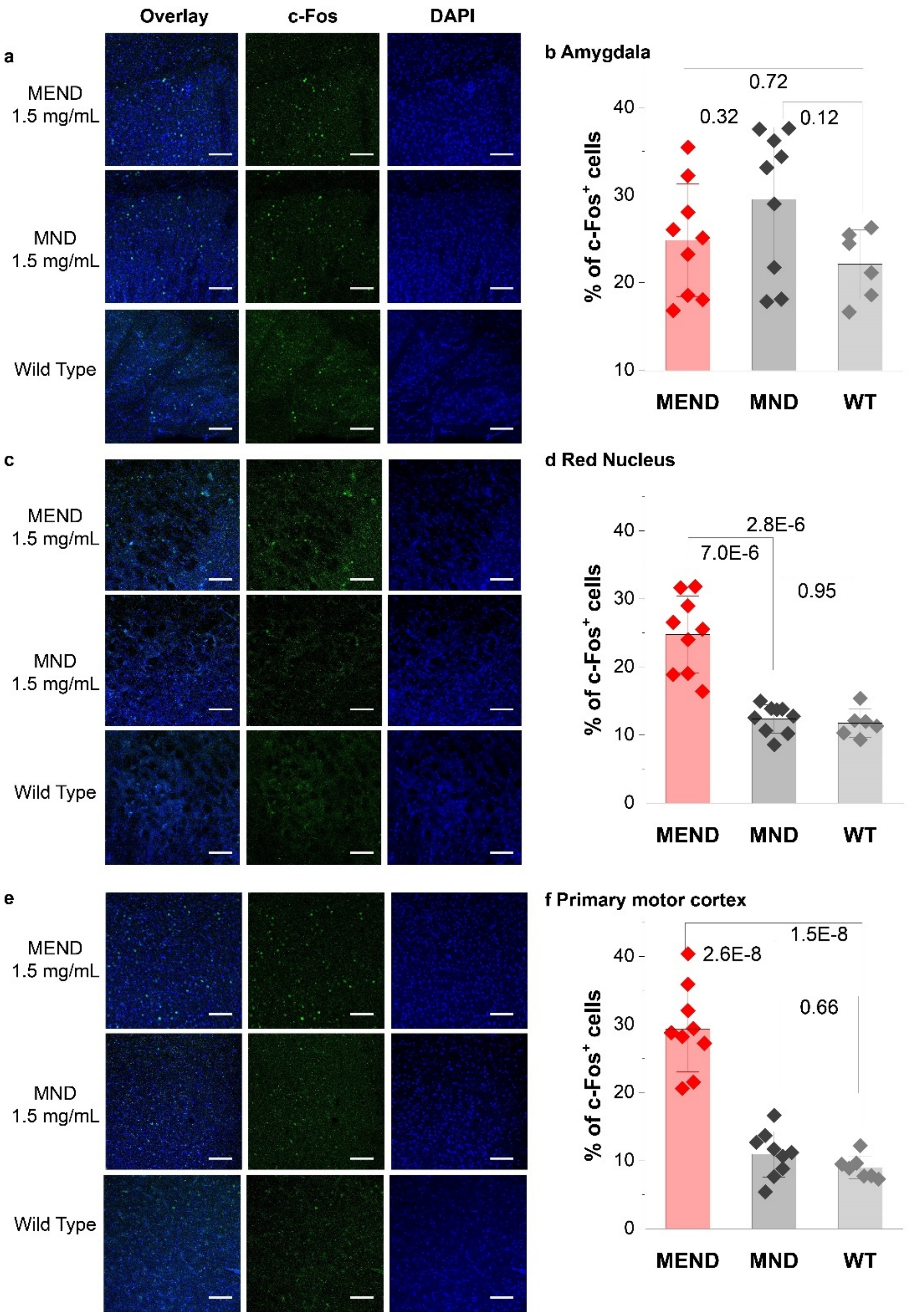

Supplementary Fig. 34| c-Fos expression in brain areas relevant to motor behavior. Representative images of DAPI, c-Fos, and their overly (**a,c,e**) and percentage of c-Fos positive cells (**b,d,f**) of **a,b** amygdala, **c,d** red nucleus, and **e,f** primary motor cortex of mice injected with MENDs (1.5 µL at 1.5 mg mL^{-1}) and MNDs (1.5 µL at 1.5 mg mL^{-1}) unilaterally in left STN of the mice. (n=9 in each group) and n=6 wild type mice. 90 min before sacrificing, 5 s MF epochs (10 mT 150 Hz AMF and 220 mT OMF) were applied with 25 s intervals for 3 min. **b,d,f,** Bars are mean and error bars are standard deviation. For statistical analysis, one-way ANOVA followed by Tukey's post-hoc comparison test was performed. P-values are noted on the plots.

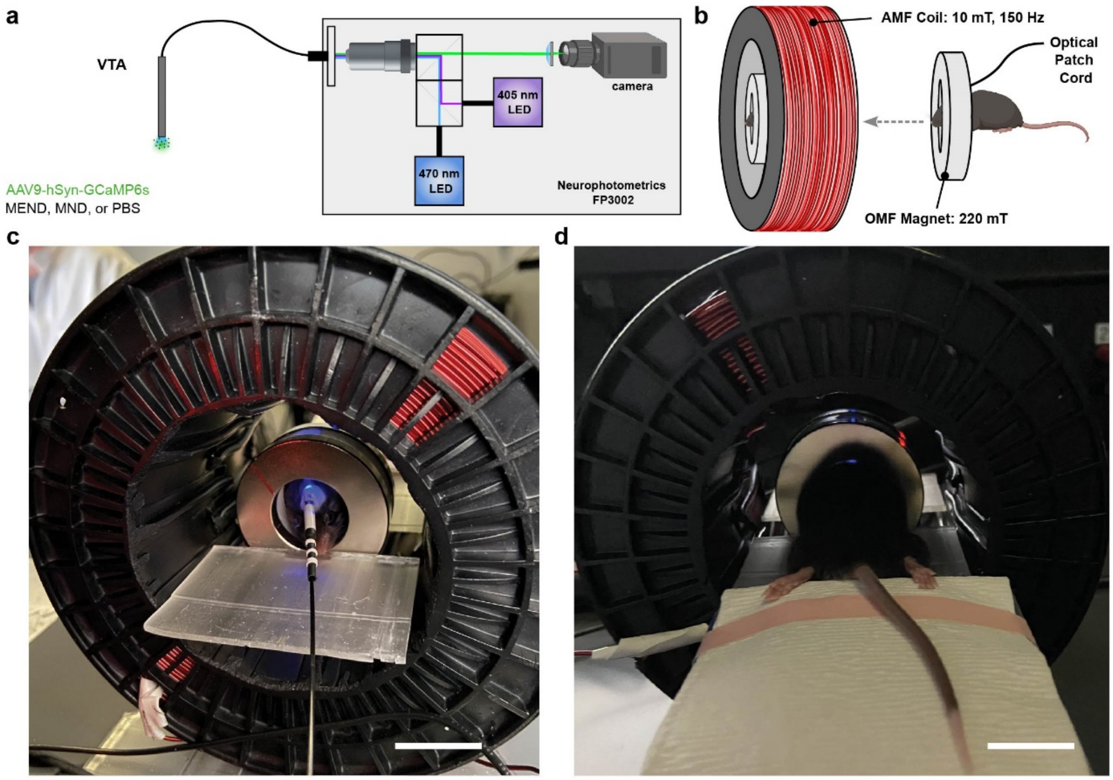

Supplementary Fig. 35| Assay for fiber photometry combined with MEND coil setup. a, An illustration of the fiber photometry system. **b,** An illustration of magnetic field generation system. **c, d,** Photographs of front and back views of the experimental setup used for fiber photometry recordings during magnetoelectric stimulation (220 mT OMF; 150 Hz, 10 mT AMF) mediated with MENDs and the corresponding control experiments. Scale bars = 2 cm.

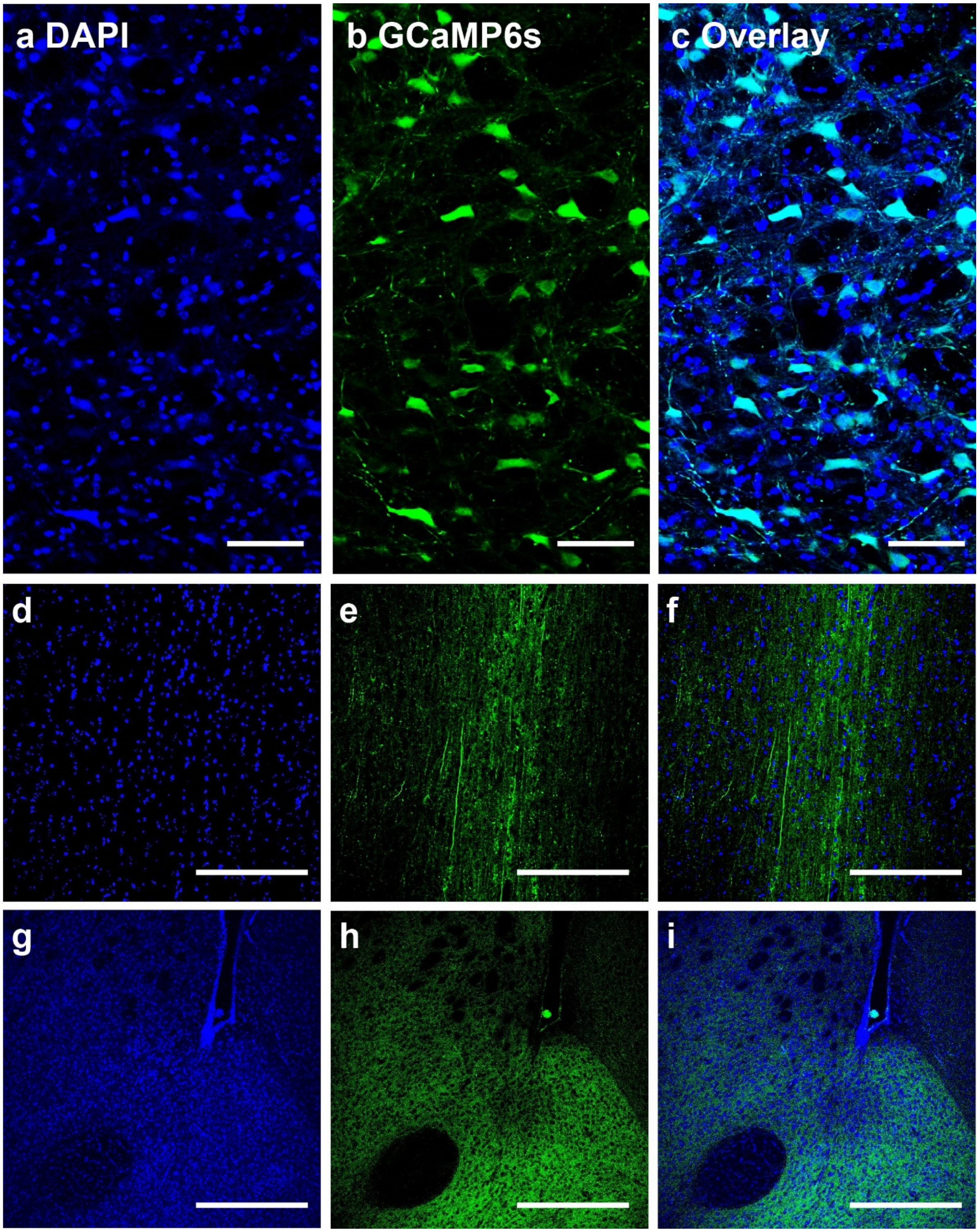

Supplementary Fig. 36| Expression of GCaMP6s on the dopaminergic circuit. a-i, Confocal images of GCaMP6s expression (green) and DAPI nuclear marker (blue) of **a-c,** the VTA (Scale bar: 50 μm) and **d-I,** the axons VTA projecting to mPFC (**d-f**) and NAc (**g-i**). **a, d, g,** DAPI-stained cells. **b,**the GCaMP6s-expressing neurons, **e** and **h,** show the GCaMP6s-expressing axons. **c, f, i,** Overlay of DAPI and GCaMP6s signals. Scale bars = 150 μm.

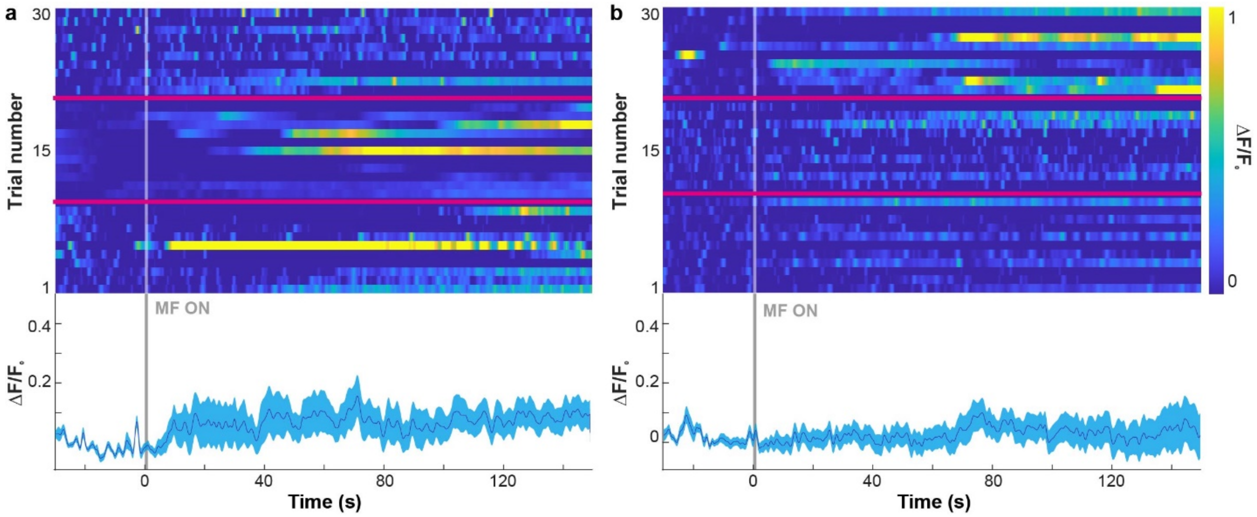

Supplementary Fig. 37| Fiber photometry of GCaMP6s fluorescence in vivo. a, b, Photometric recordings of average relative GCaMP6s fluorescence in the VTA of the mice injected with **a,** MND (1.5 mg mL^{-1}) and **b,** PBS. Bottom plots: lines and shading areas represent average and standard deviation (n=3 mice per condition; 10 trials per mouse). Vertical grey bars denote magnetic field epochs (2 s, 220 mT OMF; 150 Hz, 10 mT AMF).

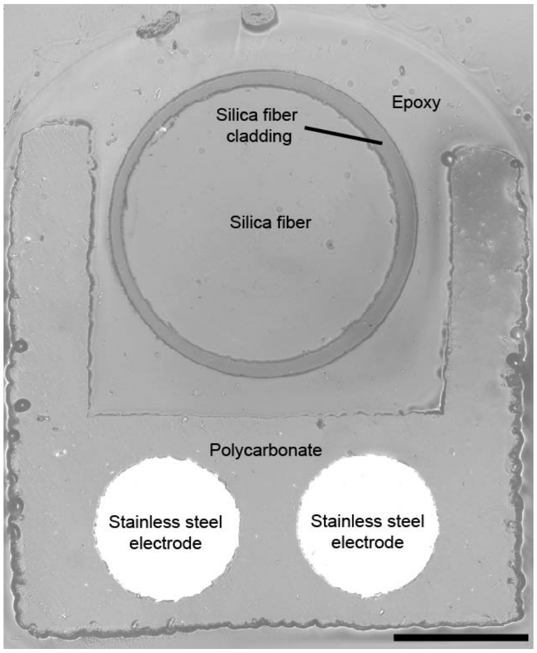

Supplementary Fig. 38| Cross section of the fiber used for simultaneous electrical stimulation and photometric recordings in vivo. Epoxy was used for fixing the silica fiber within the polycarbonate cladding. Scale bar = 100 μm

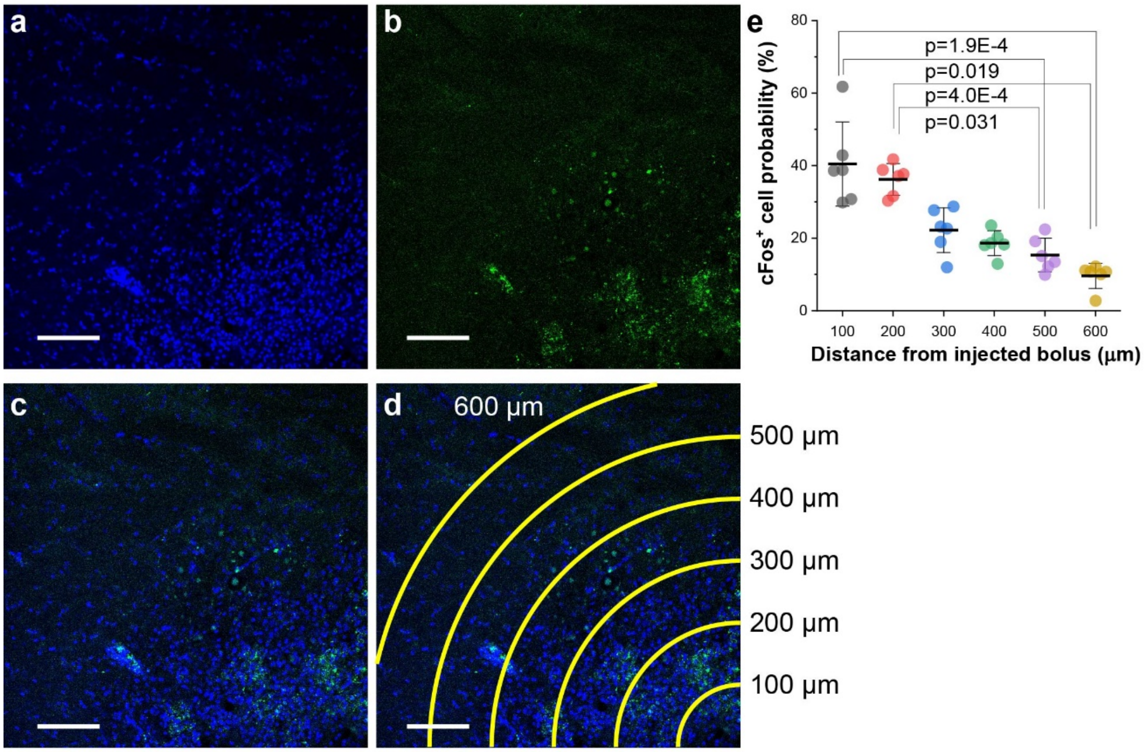

Supplementary Fig. 39| Spatial resolution of MEND-mediated brain stimulation. a-c, Confocal images of **a,** DAPI, **b,** c-Fos, and **c,** Overlay in brain slices of a mouse injected with MENDs (1.5 µL at 0.5 mg mL^{-1}) and exposed to magnetic field. Scale bars = 100 µm. **d,** Distance from the center of injected MEND bolus is indicated with yellow circles spaced by 100 µm. **e,** Percentage of c-Fos expressing cells per 100 µm section in **d**. Markers represent data points and error bars correspond to SD. For statistical analysis, Kruskal Wallis followed by Tukey's post-hoc comparison test was applied as the data for 600 µm rejects normality. P>0.05 is not indicated, while other p-values are noted on the plot.

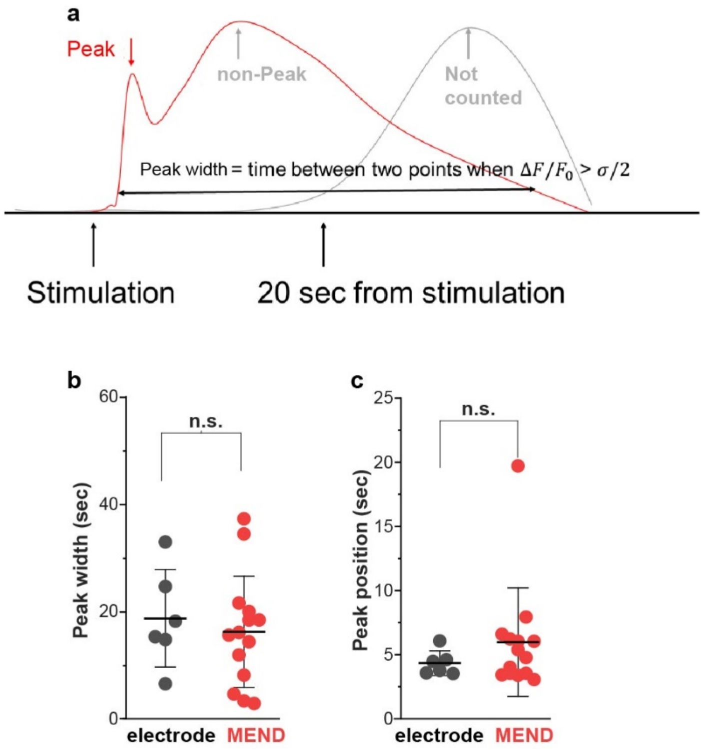

Supplementary Fig. 40 | Photometry Peak Analysis. a, The peaks were defined when the intensity $\Delta F/F_0 > 2\sigma$, where σ is the average of $\Delta F/F_0$ during15s prior to MF onset. Spiking probability is the fraction of trials exhibiting a peak of a GCaMP6s transient within 20 s from the MF onset. The peak width and position was measured only for the first maxima appearing within a 20 s window from MF onset. The peak position was defined as the time point corresponding to the maximum $\Delta F/F_0$, and the peak width is the time between the time points when the intensity becomes $\sigma/2$ before and after the maxima. **b,** The width and **c,** position of the peaks in the GCaMP6s $\Delta F/F_0$ traces from the mice stimulated via MENDs/MF (n=14) or electrodes (n=6), which was analyzed using the peak of the first transient definition described in **a**. The error bars and their center lines indicate the mean and SD. The statistical analysis, Mann-Whitney U test was performed because MEND data are not following the normality. For peak width, p=0.48, and for peak position, p=0.59

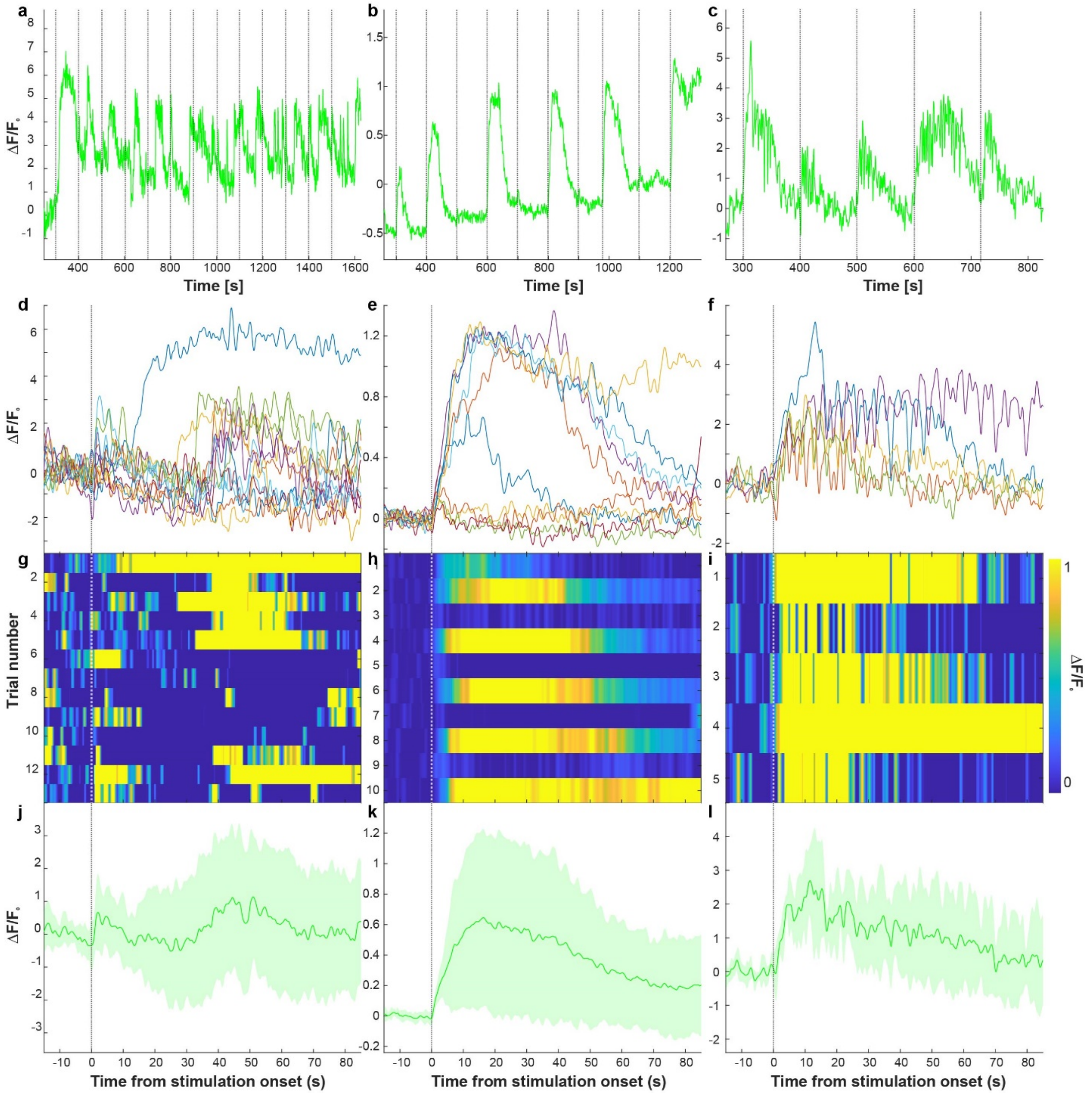

Supplementary Fig. 41 | Evidence of primary and secondary (slower and wider) transients in individual photometry trials during MEND-mediated simulation. a-c, Examples of GCaMP6s traces during repeated MF epochs in MEND-injected mice. **d-f,** GCaMP6s traces from (a-c) segmented from –15s to 85s for every MF epoch. **g-i,** Heat maps of the segmented traces from (d-f). **j-l,** GCaMP6s averages across the trials (lines) shown in (d-f), shown with the s.e.m. bands (shaded areas). **a,d,g,j** are from the second animal from the top in Fig. 5b. **b,e,h,k** are from the sixth animal from the top in Fig. 5b. **c,f,i,l** are from the second animal from the bottom in Fig. 5b. The dotted lines denote the MF onset.

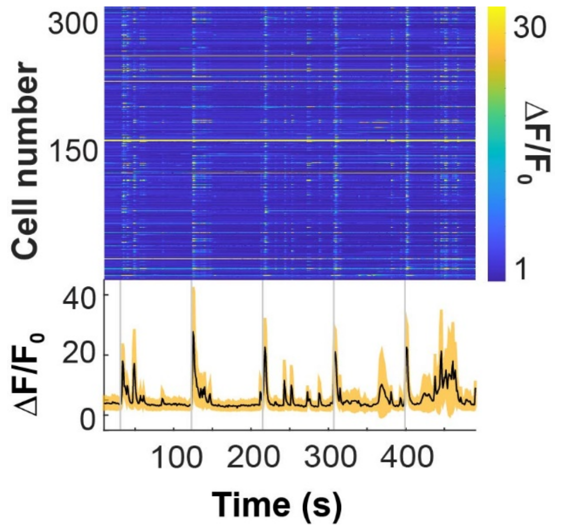

Supplementary Fig. 42| GCaMP6s fluorescence imaging of neurons cultured without FUDR. Imaging with 90 s intervals between 2 s MF (100 Hz 10 mT AMF and 220 mT OMF) epochs was performed on 300 primary hippocampal neurons (n=3 culture plates) cultured without FUDR treatment to prevent suppression of glial cells' growth. Bottom panel: line and shaded area represent mean and s.e.m.

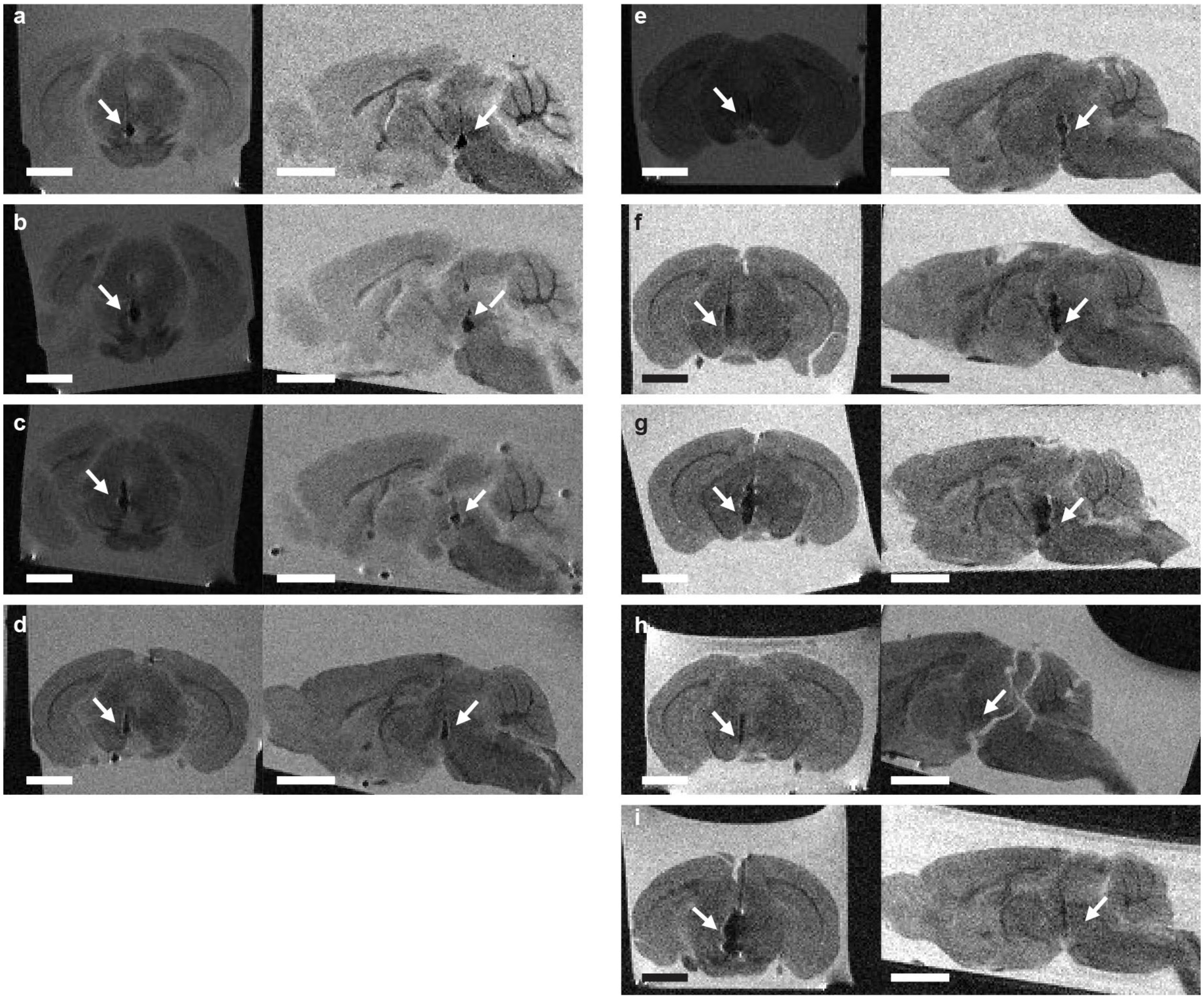

Supplementary Fig. 43| MRI images of mouse brains 2 weeks and 2 months following MEND injection in the VTA. MRI images of the brains at (**a-d**) 2 weeks and (**e-i**) 2 months following unilateral injections of MENDs into the left VTA. Left = coronal view, scale bars = 2 mm; right = sagittal view, scale bar = 3 mm. Arrows denote the injected MEND boluses.

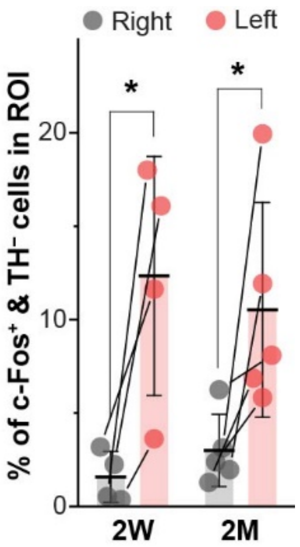

Supplementary Fig. 44| Percentage of c-Fos-expressing cells among the non-TH-expressing cells in the left and right VTA at 2 weeks and 2 months following unilateral MEND injections. Data (markers) are shown with mean (columns), and error bars correspond to SD. Paired t-test was performed as the data are normally distributed. For 2 weeks (2W), p=0.041, and for 2 months (2M), p=0.049.

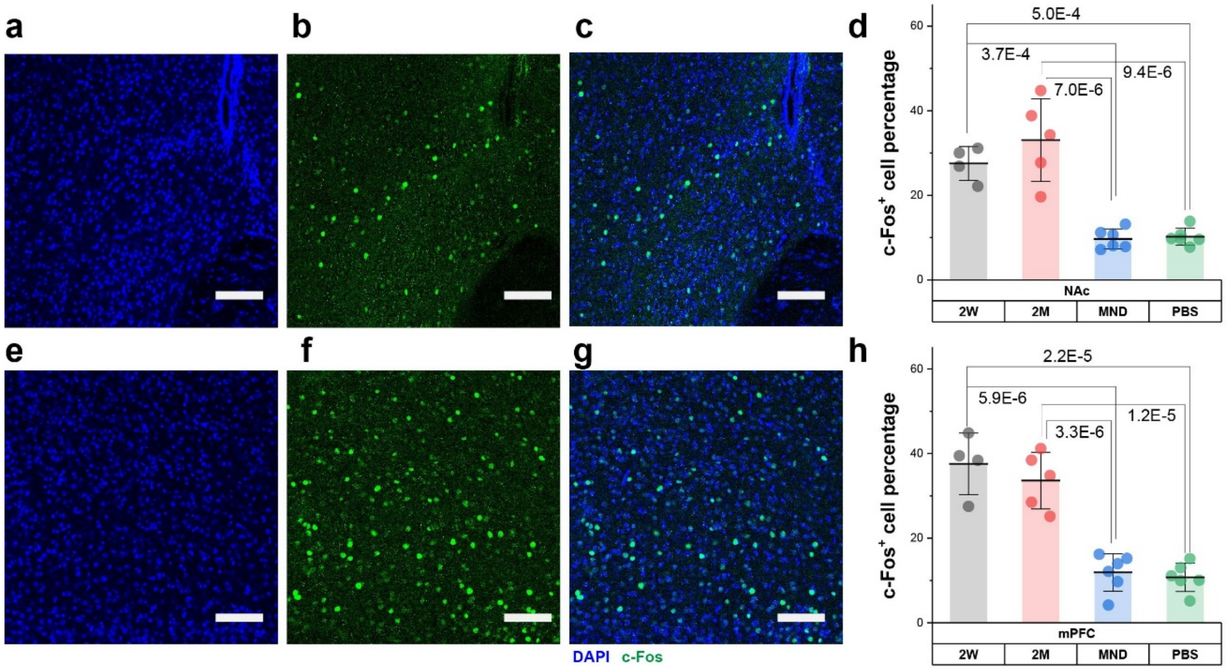

Supplementary Fig. 45| c-Fos imaging on NAc and mPFC of the mice stimulated via MF application 2 weeks (2W) and 2 months (2M) after the MEND injection surgery. MENDs (1.5 μL at 1.5 mg mL^{-1}) were injected to the mice unilaterally in left VTA of the mice. (n=4) **a-c,** Representative images of (**a**)DAPI, (**b**)c-Fos, and (**c**) their overly of NAc. **d,** Comparison of c-Fos positive cells percentage with the data taken from MND- and PBS-injected mice (Fig. S29). **e-g,** Representative images of (**e**)DAPI, (**f**)c-Fos, and (**g**) their overly of mPFC. **h,** Comparison of c-Fos positive cells percentage with the data taken from MND- and PBS-injected mice (Fig. S30). **d,h,** Data are shown with mean and SD. One-way ANOVA followed by Tukey's post-hoc comparison test was performed for statistical analysis. P-values are noted on plots.

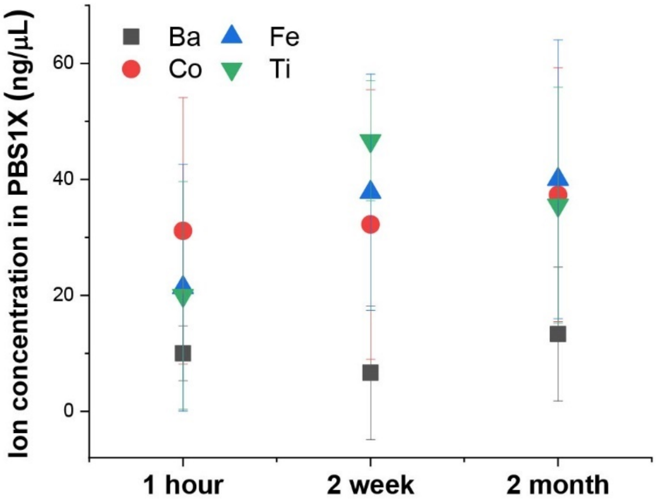

Supplementary Fig. 46| Stability of MENDs in saline solution. Inductively Coupled Plasma-Mass Spectrometry (ICP-MS) analysis of PBS in which MENDs were soaked for 1 hour, 2 weeks, and 2 months. Error bars indicate SD from three samples for each data point, and each data point indicates the mean value of the three samples.

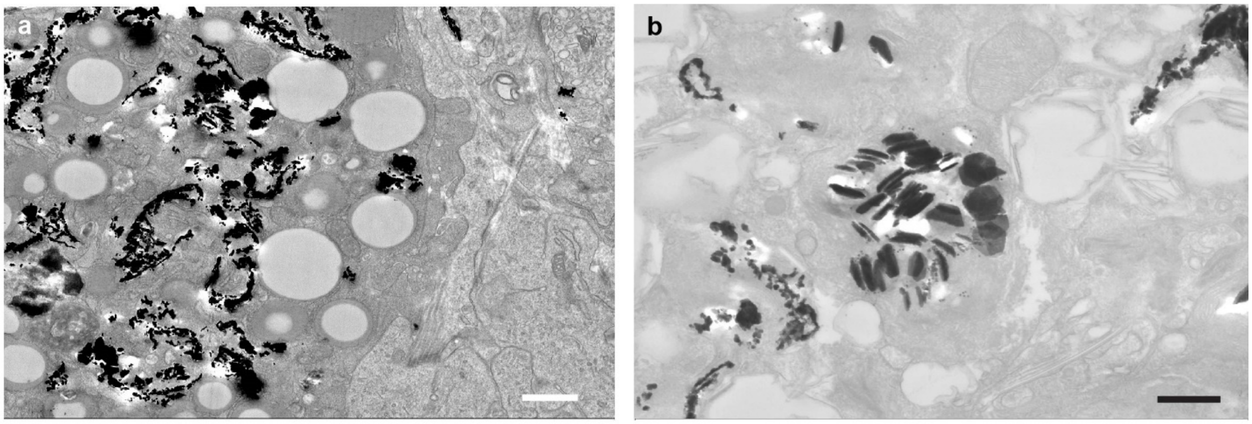

Supplementary Fig. 47| TEM imaging on tissue from the mouse VTA 2 weeks following MEND injection. The samples were prepared from mice sacrificed 2 weeks after MEND (1.5 μL at 1.5 mg mL^{-1}) injection into the VTA. MENDs exhibit darker contrast than the surrounding tissue. **a,** Endocytosed MENDs. **b,** The image acquired at a higher magnification to confirm that the contrast corresponds to MENDs. These images are consistent with prior observations of cellular uptake of nanomaterials injected into the brain.[12,18] Scale bar in **a** = 1 μm, and scale bar in **b** = 500 nm. MEND injection area was identified in coronal brain slices and extracted with a biopsy punch. Tissue punches were then stained in reduced osmium (1.25 wt% potassium ferrocyanide and 1 wt% osmium tetroxide buffered in 0.1 M sodium cacodylate) for 1 hour, and en bloc stained overnight in maleate buffered 2% uranyl acetate. Stained samples were dehydrated in a graded ethanol series, transitioned through propylene oxide, and infiltrated with EMbed 812 resin. Resin blocks were polymerized for 48 hours at 60°C. Ultrathin 60 nm sections were prepared using a Leica UC7 Ultramicrotome and mounted onto Formvar-Carbon coated copper slot TEM grids. Scanning TEM (STEM) imaging of the sections was performed on a Zeiss Crossbeam 540 at 30kV.

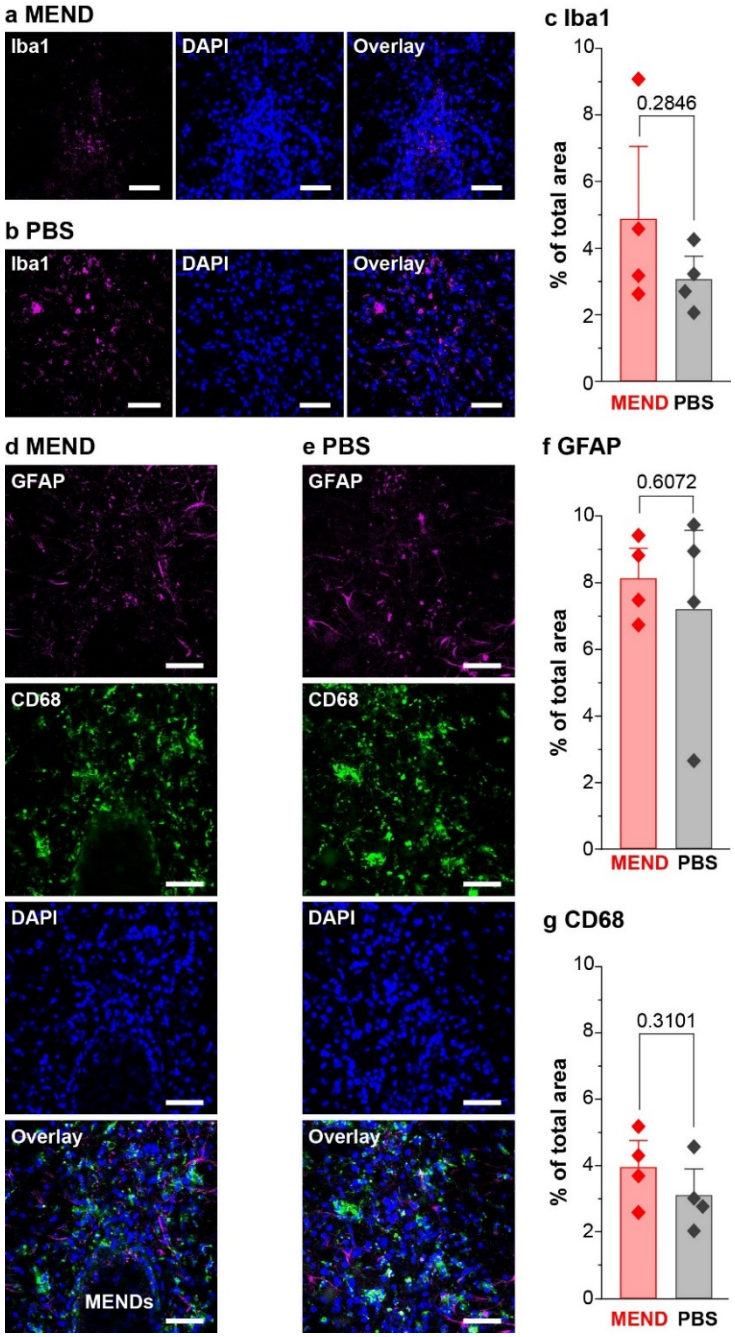

Supplementary Fig. 48| Biocompatibility assessment of MENDs (1.5 µL at 1.5 mg mL^{-1}) unilaterally injected into the VTA as compared to PBS injections contralaterally. a, b, d, e, Representative confocal images and **c, f, g,** average percentages of expression of **a-c,** Iba1, **d,f,** GFAP (**d,f**), and **e, g,** CD68 in the brains of mice 2 weeks following surgery for unilateral injection with MENDs and contralateral injection with PBS. 90 min before the perfusion, the animals were applied to the magnetic field (220 mT DC and 150 Hz, 10 mT AC; three 2 s epochs separated by 90 s rest epochs). Scale bars = 50 µm. Two-sample t-test was performed (n=4 mice per group, P values are noted on plots in panels **c, f,** and **g**). The error bars and their center line indicate the mean and SD.

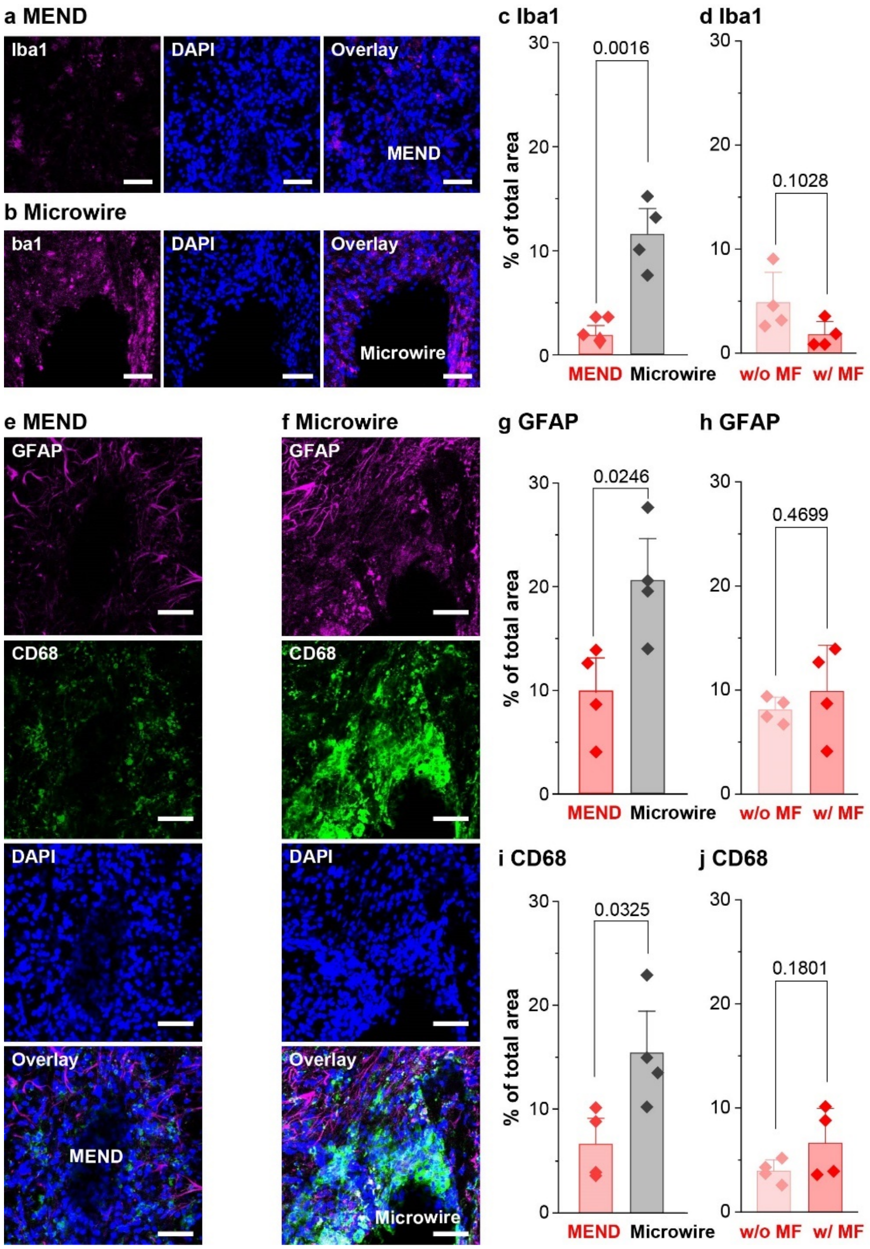

Supplementary Fig. 49| Biocompatibility assessment of MENDs (1.5 µL at 1.5 mg mL^{-1}) unilaterally injected into the VTA as compared to a 300 µm stainless steel microwire implanted contralaterally. Representative confocal images (**a, e, f**) and average percentages (**c, d, g-j**) of areal coverage of expression across conditions of Iba1 (**a-c**), GFAP (**e-g**), and CD68 (**e, f, i**) in the brain of mice 2 weeks following surgery for unilateral MENDs injection and contralateral implantation of 300 µm stainless steel microwire. (**d, h, j**) The comparison of the three markers in the MEND-injected brains 2 weeks after the surgery with (data copied from panel c, g, i) and without (data copied from Fig. S47) the magnetic field application. Scale bars = 50 µm. A two-sample t-test was performed (n=4 mice per group, P-values are noted on plots in panels c, d, g-j). The error bars and their center line indicate the mean and SD.

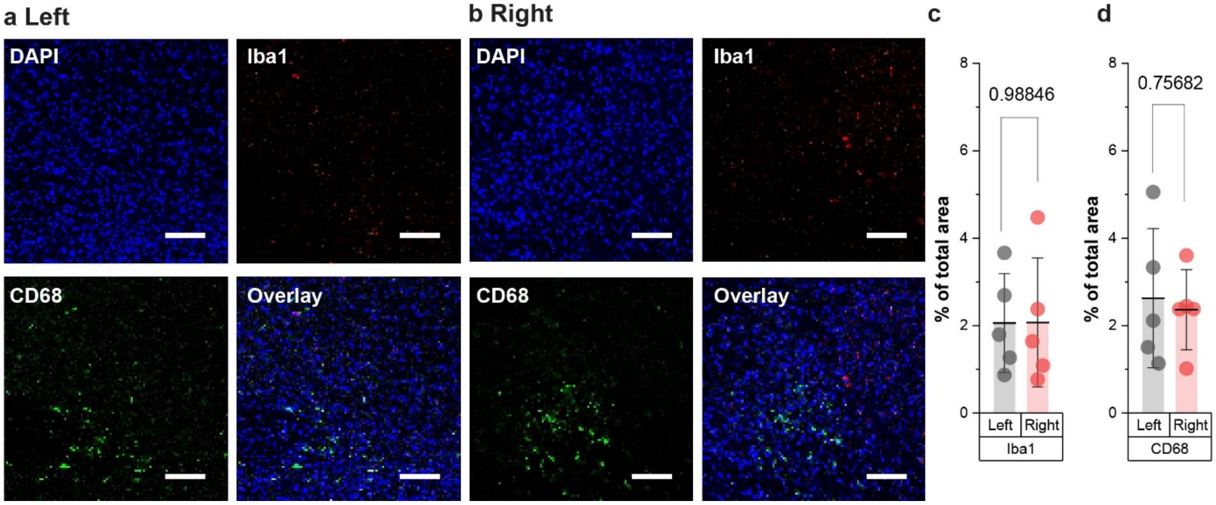

Supplementary Fig. 50| Biocompatibility assessment of MENDs (1.5 µL at 1.5 mg mL^{-1}) unilaterally injected into the left VTA as compared to the right VTA. Representative confocal images (**a, b**) and average percentages (**c, d**) of expression across conditions of Iba1 CD68 in the brain of mice 2 months following surgery for unilateral MENDs injection. Scale bars = 50 µm. A two-sample t-test was performed (n=5 mice per group, P values are noted on plots in panels c and d). The error bars indicate SD. As all data follow a normal distribution, paired t-test was performed.

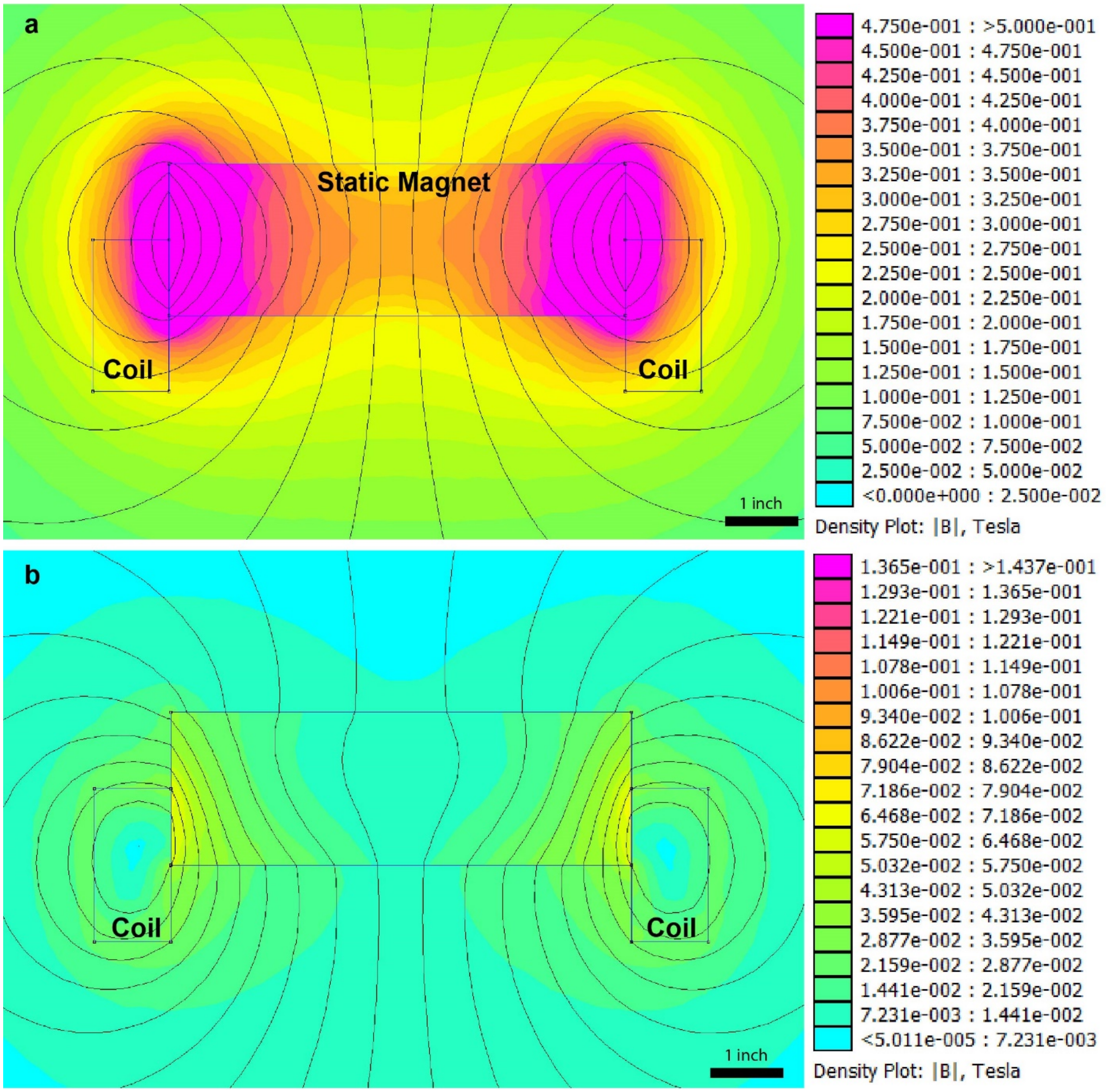

Supplementary Fig. 51| Finite Element Method Magnetics simulation of helmet for applications in translational models. a, MF from a 6” diameter, 2” height NdFeB n52 permanent magnet combined with a AMF coil (3” inner diameter) comprising 1000 turns of 18AWG wire conducting 3 A current. **b,** AMF coil without the permanent magnet generates 10 mT at 100 Hz.

Captions for Supplementary Videos

Supporting Video 1. GCaMP6s fluorescence changes (10X speed) in primary hippocampal neurons decorated with MENDs in response to 10s pulses of combined offset magnetic field (OMF) 220 mT and alternating magnetic field (AMF) 10 mT, 150 Hz.

Supporting Videos 2. GCaMP6s fluorescence changes (real time) in primary hippocampal neurons decorated with MENDs in response to 10s magnetic field epochs with the variation in the AMF frequency, while maintaining its amplitude (10 mT) and the magnitude of OMF (220 mT).

Supporting Video 3. GCaMP6s fluorescence changes (real time) in primary hippocampal neurons decorated with MENDs in response to 2 s epochs of combined 220 mT OMF and 10 mT, 150 Hz AMF. Separation between stimulation epochs was 120s, 90s, 60s, 30s, and 10s, and each stimulation sequence was repeated three times.

Supporting Video 4. GCaMP6s fluorescence changes (real time) in primary hippocampal neurons decorated with MENDs in response to 2 s epochs of 220 mT OMF and 10mT, 150 Hz AMF in the presence of 1 μM tetrodotoxin.

Supporting Video 5. GCaMP6s fluorescence changes (real time) in primary hippocampal neurons decorated with MENDs in response to 2 s epochs of 220 mT OMF and 10mT, 150 Hz AMF in the presence of 20 μM 6-cyano-7-nitroquinoxaline-2,3-dione (CNQX) and 100 μM (2R)-amino-5-phosphonovaleric acid (AP5).

Supporting Video 6. Representative videos of a place preference assay with mice injected with MENDs and MNDs (1.5 μL at 1.5 mg mL^{-1} unilaterally in the left VTA) recorded from the side of the arena opposite to the stimulation chamber (220 mT OMF and 10mT, 150 Hz AMF)

Supporting Video 7. Representative 3-min videos of the cylinder test for a naïve mouse and a mouse unilaterally injected with MENDs in the left STN (1.5 μL at 1.5 mg mL^{-1}) during MF stimulation (5s epochs, 25 s intervals, 220 mT OMF and 10mT, 150 Hz AMF).

Supporting references

1 Mishra, B. N. & Chatterjee, S. Study of Piezoelectric Properties of Barium Titanate for Energy Harvesting System Using COMSOL.

2 Eisenberg, R. S. & Johnson, E. A. Three-dimensional electrical field problems in physiology. *Progress in biophysics and molecular biology* **20**, 1-65 (1970).

3 Geisler, C. D. & Goldberg, J. M. A stochastic model of the repetitive activity of neurons. *Biophysical journal* **6**, 53-69 (1966).

4 Jack, J. J. B., Noble, D. & Tsien, R. W. Electric current flow in excitable cells. (1975).

5 Neudorfer, C. *et al.* Kilohertz-frequency stimulation of the nervous system: A review of underlying mechanisms. *Brain stimulation* **14**, 513-530 (2021).

6 Lodish, H. *et al.* Molecular cell biology 4th edition. *National Center for Biotechnology Information, Bookshelf* **9** (2000).

7 Moyes, C. D. & Schulte, P. M. *Animal Physiology*. (Benjamin Cummings San Francisco, CA, 2005).

8 Skaar, J.-E. W., Stasik, A. J., Hagen, E., Ness, T. V. & Einevoll, G. T. Estimation of neural network model parameters from local field potentials (LFPs). *PLoS computational biology* **16**, e1007725 (2020).

9 Kaech, S. & Banker, G. Culturing hippocampal neurons. *Nature protocols* **1**, 2406-2415 (2006).

10 Hu, W. *et al.* Distinct contributions of Nav1. 6 and Nav1. 2 in action potential initiation and backpropagation. *Nature neuroscience* **12**, 996-1002 (2009).

11 Beaulieu-Laroche, L. *et al.* Enhanced dendritic compartmentalization in human cortical neurons. *Cell* **175**, 643-651. e614 (2018).

12 D'Agata, F. *et al.* Magnetic nanoparticles in the central nervous system: targeting principles, applications and safety issues. *Molecules* **23**, 9 (2017).

13 Cheikh, Z. B. *et al.* Hydrogen doped BaTiO3 films as solid-state electrolyte for micro-supercapacitor applications. *Journal of Alloys and Compounds* **721**, 276-284 (2017).

14 Sinclair, N. C. *et al.* Deep brain stimulation for Parkinson's disease modulates high-frequency evoked and spontaneous neural activity. *Neurobiology of disease* **130**, 104522 (2019).

15 Couto, J. & Grill, W. M. Kilohertz frequency deep brain stimulation is ineffective at regularizing the firing of model thalamic neurons. *Frontiers in computational neuroscience* **10**, 22 (2016).

16 Benabid, A. L. *et al.* Long-term suppression of tremor by chronic stimulation of the ventral intermediate thalamic nucleus. *The Lancet* **337**, 403-406 (1991).

17 Papale, A. E. & Hooks, B. M. Circuit changes in motor cortex during motor skill learning. *Neuroscience* **368**, 283-297 (2018).

18 Cupaioli, F. A., Zucca, F. A., Boraschi, D. & Zecca, L. Engineered nanoparticles. How brain friendly is this new guest? *Progress in neurobiology* **119**, 20-38 (2014).

19 Coey, J. M. *Magnetism and magnetic materials*. (Cambridge university press, 2010).

20 Kemp, S. J., Ferguson, R. M., Khandhar, A. P. & Krishnan, K. M. Monodisperse magnetite nanoparticles with nearly ideal saturation magnetization. *RSC advances* **6**, 77452-77464 (2016).

21 Leite, G. C. *et al.* Exchange coupling behavior in bimagnetic CoFe2O4/CoFe2 nanocomposite. *Journal of Magnetism and Magnetic Materials* **324**, 2711-2716 (2012).

22 Jain, A. *et al.* Commentary: The Materials Project: A materials genome approach to accelerating materials innovation. *APL materials* **1**, 011002 (2013).

23 Lavorato, G., Winkler, E., Rivas-Murias, B. & Rivadulla, F. Thickness dependence of exchange coupling in epitaxial Fe 3 O 4/CoFe 2 O 4 soft/hard magnetic bilayers. *Physical Review B* **94**, 054405 (2016).

24 Rondinone, A. J., Samia, A. C. & Zhang, Z. J. Characterizing the magnetic anisotropy constant of spinel cobalt ferrite nanoparticles. *Applied Physics Letters* **76**, 3624-3626 (2000).

25 O'handley, R. C. *Modern magnetic materials: principles and applications*. (Wiley, 2000).

26 Lüthi, B. Magnetoacoustic birefringence in magnetite and nickel. *Applied Physics Letters* **8**, 107-109 (1966).
