# Supplementary material for: Magnetoelectric nanodiscs enable wireless transgene-free neuromodulation
Source: Nat Nanotechnol. 2024 Oct 11;20(1):121–31. doi: 10.1038/s41565-024-01798-9 (PMC11750723; doi:10.1038/s41565-024-01798-9)
Supplement: Supplementary file 2 — Reporting Summary [file 41565_2024_1798_MOESM2_ESM.pdf]

nature research

Corresponding author(s): Polina Anikeeva

Last updated by author(s): Aug 27, 2024

Reporting Summary

Nature Research wishes to improve the reproducibility of the work that we publish. This form provides structure for consistency and transparency in reporting. For further information on Nature Research policies, see our Editorial Policies and the Editorial Policy Checklist.

Statistics

For all statistical analyses, confirm that the following items are present in the figure legend, table legend, main text, or Methods section.

n/a	Confirmed	
☐	☒	The exact sample size (*n*) for each experimental group/condition, given as a discrete number and unit of measurement
☐	☒	A statement on whether measurements were taken from distinct samples or whether the same sample was measured repeatedly
☐	☒	The statistical test(s) used AND whether they are one- or two-sided<br>*Only common tests should be described solely by name; describe more complex techniques in the Methods section.*
☐	☒	A description of all covariates tested
☐	☒	A description of any assumptions or corrections, such as tests of normality and adjustment for multiple comparisons
☐	☒	A full description of the statistical parameters including central tendency (e.g. means) or other basic estimates (e.g. regression coefficient) AND variation (e.g. standard deviation) or associated estimates of uncertainty (e.g. confidence intervals)
☐	☒	For null hypothesis testing, the test statistic (e.g. *F*, *t*, *r*) with confidence intervals, effect sizes, degrees of freedom and *P* value noted<br>*Give P values as exact values whenever suitable.*
☒	☐	For Bayesian analysis, information on the choice of priors and Markov chain Monte Carlo settings
☒	☐	For hierarchical and complex designs, identification of the appropriate level for tests and full reporting of outcomes
☒	☐	Estimates of effect sizes (e.g. Cohen's *d*, Pearson's *r*), indicating how they were calculated

Our web collection on statistics for biologists contains articles on many of the points above.

Software and code

Policy information about availability of computer code

Data collection	COMSOL Multiphysics (version 5.6) was used for modeling electric polarization in piezoelectric shell formed on the surface of magnetostriction core. Mummax3 was used for modeling magnetostriction. MATLAB (R2023b) software was used for neuronal simulation. cellSence was used for imaging with inverted microscope.
Data analysis	FIJI (ImageJ 1.53g) was used to analyze immune response from injected particles by quantifying the fluorescence area. MATLAB (R2023b) was used for analyzing c-Fos expressing level in the cells around injected particles. For calcium and voltage imaging, FIJI (ImageJ 1.53g) was used to extract quantified fluorescence traces of individual cell in the video, and MATLAB (R2023b) was used to analyze the fluorescence traces. Plots were generated using OriginPro 2019. OriginPro 2019 or MATLAB (R2023b) software was used to assess the statistical significance of all comparison studies. Graphical illustration and figures were produced using Adobe Illustrator 2020, Power Point, and BioRender.

For manuscripts utilizing custom algorithms or software that are central to the research but not yet described in published literature, software must be made available to editors and reviewers. We strongly encourage code deposition in a community repository (e.g. GitHub). See the Nature Research guidelines for submitting code & software for further information.

Data

Policy information about availability of data

All manuscripts must include a data availability statement. This statement should provide the following information, where applicable:

- Accession codes, unique identifiers, or web links for publicly available datasets
- A list of figures that have associated raw data
- A description of any restrictions on data availability

All data and code accompanying the manuscript are available on Figshare.

Field-specific reporting

Please select the one below that is the best fit for your research. If you are not sure, read the appropriate sections before making your selection.

- [x] Life sciences
- [ ] Behavioural & social sciences
- [ ] Ecological, evolutionary & environmental sciences

For a reference copy of the document with all sections, see nature.com/documents/nr-reporting-summary-flat.pdf

Life sciences study design

All studies must disclose on these points even when the disclosure is negative.

Sample size	Group sizes were chosen based on previous research conducted in the same brain circuits
Data exclusions	For place preference behavioral studies, mice that showed more than 70% preference to any chamber on the pre-test day were excluded from subsequent analysis. Mice that showed more than 500 s (total test time is 600 s) preference to a chamber during pre-test or stayed 0 s at the stimulated chamber on Day 2 were eliminated from the subsequent analyses.
Replication	The number of replicates for each experiment are reported in the main manuscript and methods. Wherever applicable, all conditions were tested across multiple trials and results reported as mean-performance along with standard deviation or standard error of the mean.
Randomization	Injection of particles and control solutions was randomly assigned to experimental groups. Animals for behavior studies were randomly assigned to treatment groups.
Blinding	Whenever possible, the data was analyzed by an independent researcher blinded to experimental conditions. For behavioral studies, blinding was not possible since the experimenter manually controlled the stimulation pattern by turning on the electromagnet. However, analysis of all animal behavior videos was performed by an independent experimenter who was blind to conditions and subject identity.

Reporting for specific materials, systems and methods

We require information from authors about some types of materials, experimental systems and methods used in many studies. Here, indicate whether each material, system or method listed is relevant to your study. If you are not sure if a list item applies to your research, read the appropriate section before selecting a response.

Materials & experimental systems

n/a	Involved in the study	
	x	Antibodies
x		Eukaryotic cell lines
x		Palaeontology and archaeology
	x	Animals and other organisms
x		Human research participants
x		Clinical data
x		Dual use research of concern

Methods

n/a	Involved in the study	
x		ChIP-seq
x		Flow cytometry
	x	MRI-based neuroimaging

Antibodies

Antibodies used	Primary Antibody: Iba1:Goat anti-Iba1, ab107159 Abcam, 1:500 dilution; GFAP: Goat anti-GFAP, ab53554 Abcam, 1:1000 dilution. Secondary antibody: Donkey anti-Goat Alexa Fluor 488, A11055, 1:1000, Thermofischer
Validation	All antibodies are commercially available and validated in several studies. Complete information is available in form of data sheets on manufacturer's website.

Animals and other organisms

Policy information about studies involving animals; ARRIVE guidelines recommended for reporting animal research

Laboratory animals	Wild type mice (C57BL/6) aged 6–8 weeks (Jackson Laboratory, Strain #:000664) were used for this study. Approximately equal number of male and female mice were used. When mice were injected with nanoparticles or control solutions, they were group housed before surgery and single housed after surgery. When mice were implanted with the firbers, they were single housed after surgery. The cages were maintained at 22 C, 12 h light/dark cycle, and 50% humidity with ad libitum access to food and water
Wild animals	The study did not involve use of wild animals
Field-collected samples	The study did not involve samples collected from the field.

Ethics oversight	All animal procedures involving brain implantation were approved by the MIT Committee on Animal Care and carried out in accordance with the National Institutes of Health Guide for the Care and Use of Laboratory Animals.

Note that full information on the approval of the study protocol must also be provided in the manuscript.

Magnetic resonance imaging

Experimental design

Design type	Ex-vivo
Design specifications	Both axial and coronal datasets were obtained with the geometric parameters of 196x196 matrix, field of view (FOV) =18mmx18mm, interleaved slice thickness of 0.3mm with no gap. Number of slices were adjusted to make sure the entire sample was covered. Each scan took 5 min and 26 seconds. Number of subject was ex-vivo brains from 9 mice.
Behavioral performance measures	N/A

Acquisition

Imaging type(s)	Structural
Field strength	7T
Sequence & imaging parameters	T2 Weighted images were obtained using the TurboRARE protocol with TR/TE=3400/35 ms, echo spacing=11.667ms, number of averages=4 and RARE factor of 8
Area of acquisition	A whole brain scan was used

Diffusion MRI ☐ Used ☒ Not used

Preprocessing

Preprocessing software	N/A
Normalization	N/A
Normalization template	N/A
Noise and artifact removal	N/A
Volume censoring	N/A

Statistical modeling & inference

Model type and settings	N/A
Effect(s) tested	N/A

Specify type of analysis: ☒ Whole brain ☐ ROI-based ☐ Both

Statistic type for inference (See Eklund et al. 2016)	N/A
Correction	N/A

Models & analysis

n/a	Involved in the study
☒	☐ Functional and/or effective connectivity
☒	☐ Graph analysis
☒	☐ Multivariate modeling or predictive analysis
